# Cobalt co-catalysis for cross-electrophile coupling: diarylmethanes from benzyl mesylates and aryl halides†
†Electronic supplementary information (ESI) available. See DOI: 10.1039/c4sc03106g



**DOI:** 10.1039/c4sc03106g

**Published:** 2014-11-10

**Authors:** Laura K. G. Ackerman, Lukiana L. Anka-Lufford, Marina Naodovic, Daniel J. Weix

**Affiliations:** a Department of Chemistry , University of Rochester , Rochester , NY 14627-0216 , USA . Email: daniel.weix@rochester.edu

## Abstract

The nickel-catalyzed cross-coupling of aryl halides with alkyl radicals derived from alkyl halides has recently been extended to couplings with carbon radicals generated by a co-catalyst. In this study, a new co-catalyst, cobalt phthalocyanine (Co(Pc)), is introduced and demonstrated to be effective for coupling substrates not prone to homolysis. This is because Co(Pc) reacts with electrophiles by an S_N_2 mechanism instead of by the electron-transfer or halogen abstraction mechanisms previously explored. Studies demonstrating the orthogonal reactivity of (bpy)Ni and Co(Pc), applying this selectivity to the coupling of benzyl mesylates with aryl halides, and the adaptation of these conditions to the less reactive benzyl phosphate ester and an enantioconvergent reaction are presented.

## Abstract 2

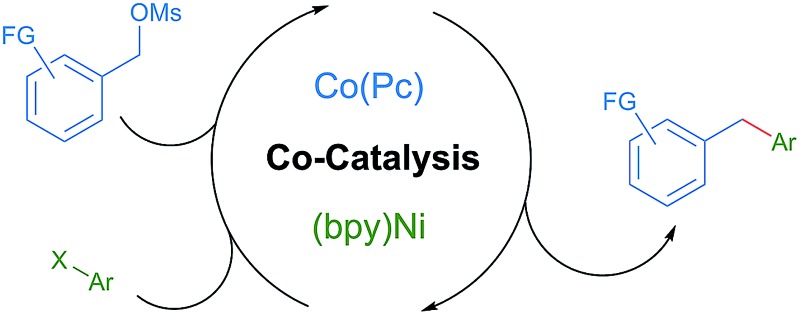
Cobalt phthalocyanine mediated generation of benzylic radicals from benzylic sulfonate esters enables the selective nickel-catalyzed synthesis of diarylmethanes.

## Introduction

**Fig. 1:**
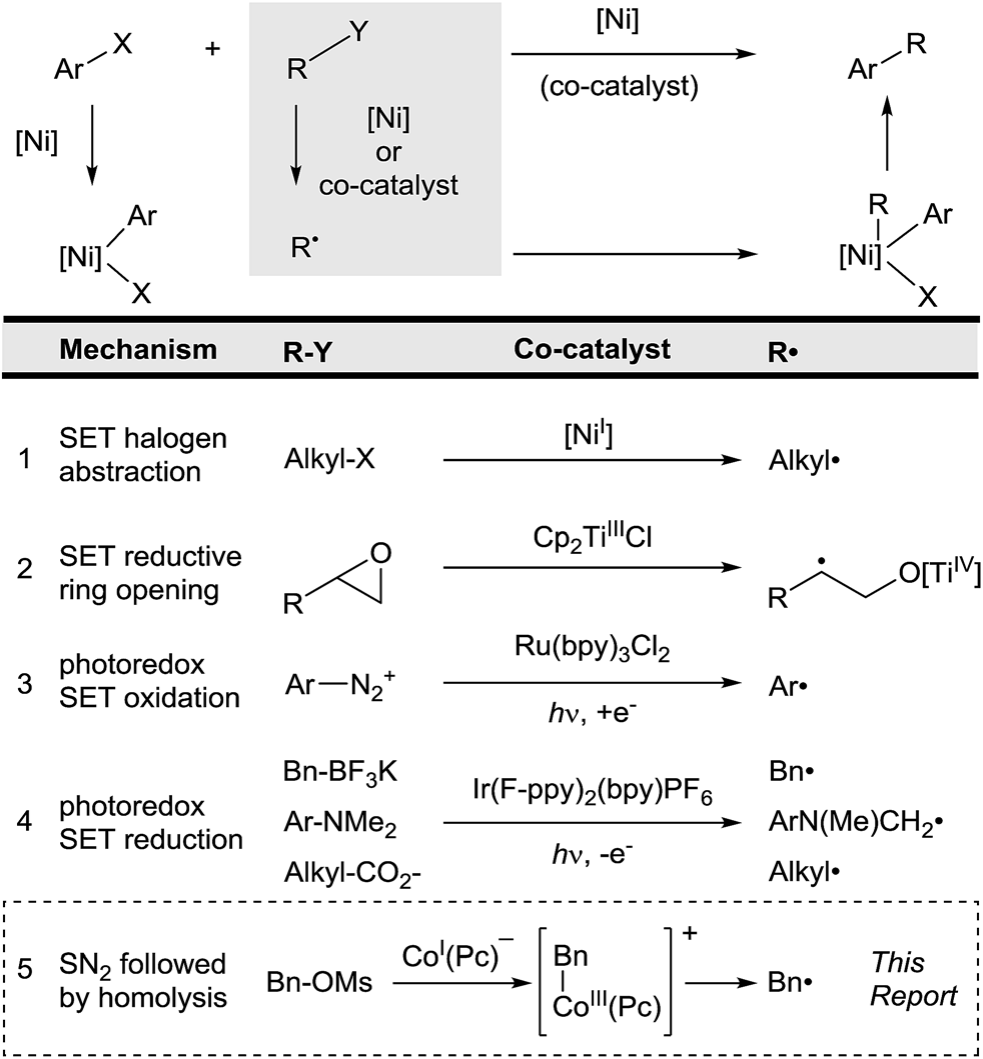
Comparison of radical co-generation methods in cross-coupling. An electrophile (Ar–X) reacts to form an arylmetal intermediate and the other substrate (R–Y) reacts to form a radical (R˙).

Cross-coupling relies upon the selective, ordered activation of two different substrates. For the coupling of nucleophiles with electrophiles, a single catalyst reacts with the electrophile by oxidative addition and the nucleophile by transmetalation, resulting in high cross-selectivities. Cross-electrophile coupling,^1^ the union of two different electrophiles, achieves selectivity by different mechanisms. Specifically, we have recently shown that in nickel-catalyzed reactions electrophiles can be differentiated by heterolysis and homolysis (Fig. 1, entry 1).^2^ Selectivity arises because (**L**)Ni^0^ reacts with aryl halides faster than alkyl halides, but (**L**)Ni^I^ forms alkyl radicals faster than aryl radicals.^3^

The key to successful cross-electrophile coupling is selective radical generation from R–Y (Fig. 1).^4^ In order to expand the types of substrates that can be coupled with aryl halides by this electrophile + radical mechanism, alternative methods of generating radicals must be developed. A key advance was that radical generation and coupling can be accomplished by two different catalysts (Fig. 1). Our group^5^ and the groups of Sanford,^6^ Molander,^7^ and MacMillan and Doyle^6*b*^ have independently shown that a variety of co-catalysts can allow coupling of otherwise unreactive substrates under mild conditions (Fig. 1, entries 2–4).

All of the methods reported to date convert the substrate into a radical by single-electron oxidation or reduction (Fig. 1). As such, substrates must be easily oxidized or reduced. *The development of co-catalysts that form radicals by different mechanisms would enable further expansion of substrate scope in this arylation strategy*.

We report here that cobalt phthalocyanine (Co(Pc)) is an excellent, non-photochemical co-catalyst for radical generation that is compatible with nickel catalysis (Fig. 1, entry 5).^8^ Co(Pc) differs from previously described co-catalysts because it generates radicals after 2-electron nucleophilic substitution^9^ rather than single-electron transfer. This gives Co(Pc) different selectivity than previous approaches. For example, alkylsulfonate and alkylphosphate esters are unreactive towards single-electron transfer due to the strength of the C–O bond, but react rapidly by nucleophilic substitution.

## Results and discussion

To demonstrate the potential of Co(Pc), we applied this co-catalyst to the synthesis of diarylmethanes from two abundant electrophiles, a benzyl alcohol derivative and an aryl halide. Although a variety of approaches to diarylmethanes have been developed,^10^ their prominence in medicinal chemistry^11^ has justified continued attention. The majority of recent approaches involve the cross-coupling of benzylmetal reagents^7,12^ or arylmetal reagents,^13^ but the need to pre-form each organometallic reagent can be limiting. A major advance was the development of methods where organozinc reagents were generated and coupled concurrently, but 2–4 equiv. of the benzyl halide was still required for high yields and no examples with more abundant benzyl alcohol derivatives^14^ were reported.^12d–f,13e^ Gosmini reported one example of the coupling of benzyl chloride with ethyl 4-bromobenzoate under conditions that might not involve an organozinc intermediate,^15^ but the scope of this method has not been explored. Finally, Reisman reported the coupling of secondary benzylic chlorides with vinyl bromides, but the use of aryl halides or benzyl alcohols was not reported.^16^ Compared to these known methods, our new approach avoids pre-formed nucleophiles, starts from benzyl alcohols instead of the less abundant benzyl halides, and does not use a large excess of one coupling partner.

**Scheme 1:**
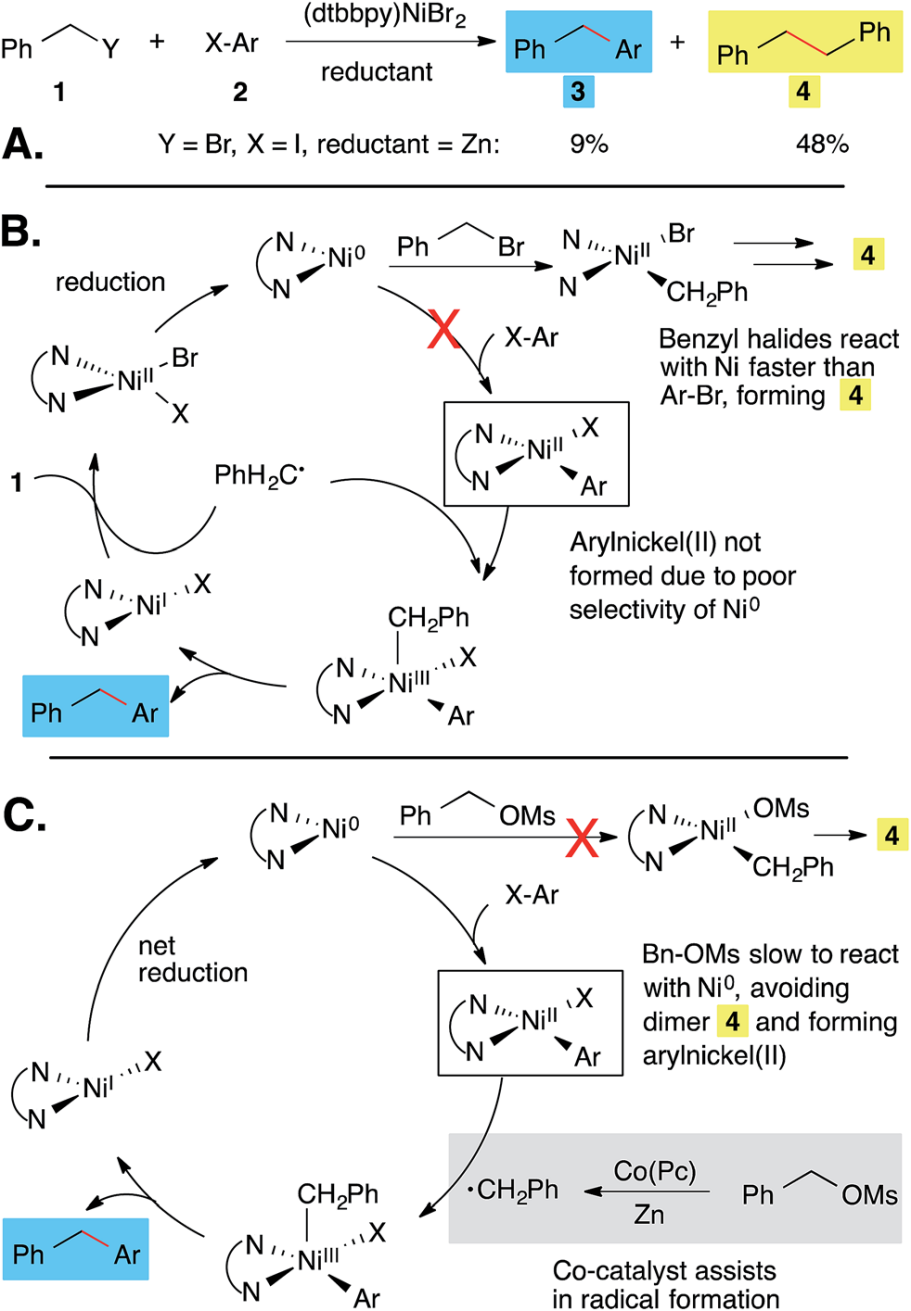
Diarylmethanes from cross-electrophile coupling: problem (A and B) and mechanism-based solution (C).^18^ Ligand = 4,4′-di-*tert*-butyl-2,2′-bipyridine.

The application of our reported conditions^1^ to the coupling of aryl halides with benzyl bromide resulted primarily in the formation of bibenzyl (Scheme 1A). This is due to the fact that benzyl bromide reacts with (**L**)Ni faster than aryl halides (Scheme 1B). For example, benzyl bromide is converted to bibenzyl and toluene in only 60 min (ESI Table S1†).

In order to restore selectivity, we sought to take advantage of the low reactivity of bipyridine-ligated nickel catalysts with alkyl sulfonate esters in order to prevent formation of benzylnickel and favor formation of arylnickel (Scheme 1C). Benzyl mesylates can be conveniently generated *in situ* from abundant benzyl alcohols. Although this approach prevented bibenzyl formation (Table S1†), it did not improve the yield of diarylmethane because nickel is slow to form a benzyl radical from a benzyl mesylate (Table 1, entry 1). The addition of Co(Pc) as a co-catalyst could generate the missing benzyl radical (Scheme 1C), separating the oxidative addition and radical generation steps.^17^

**Table 1:** Cross-electrophile coupling of Bn-OH with Ar–X^*a*^

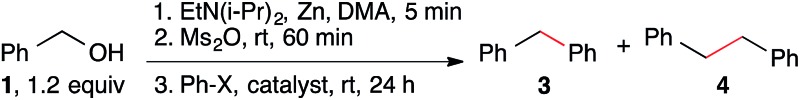				
Entry	X	Catalyst^*b*^	Yield **3** ^*c*^ (A%)	**3** : **4**
1	Br	[Ni] only	3	1 : 1
2	Br	Co(Pc) only	0	ND : 3
3	Br	[Ni], Co(Pc)	73	1 : ND
4	Br	[Ni], CoCl_2_	4	4 : 1
5	Br	[Ni], NaI (25 mol%)	25	1 : 1
6	Br	[Ni], Co(Pc), Mn instead of Zn	64	4 : 1
7	Br	NiBr_2_·3H_2_O, Co(Pc)	1	1 : 6
8	Br	No [Ni] and no Co(Pc)	0	0 : 1
9^*d*^ ^,^ ^*e*^	I	[Ni] only	71	7 : 1
10^*e*^	I	Co(Pc) only	0	ND : 1
11^*d*^ ^,^ ^*e*^	I	[Ni], Co(Pc)	83	17 : 1
12	I	[Ni], Co(Pc)	83	83 : 1
13	I	[Ni], NaI (25 mol%)	64	8 : 1
14	I	[Ni], Co(Pc), Mn instead of Zn	42	1.4 : 1

^*a*^Reactions run at 0.25 M in DMA. BnOMs was formed *in situ* from BnOH, Ms_2_O (1.44 equiv.), and EtN(i-Pr)_2_ (1.6 equiv.). See ESI.

^*b*^[Ni] = 7 mol% NiBr_2_·3H_2_O and 5 mol% dtbbpy.^19^ Co(Pc) = 1 mol% cobalt phthalocyanine.

^*c*^Yields and ratios reported as GC area%.

^*d*^Reaction run at 60 °C.

^*e*^Reaction complete within 1 h.

Significantly, this optimized nickel and cobalt system allows for the coupling of benzyl mesylates with aryl bromides, which occurs in low yield in the absence of Co(Pc) (Table 1, entries 1–3).^20^ Furthermore, it appears that both (dtbbpy)NiBr_2_ and Co(Pc) are required for high selectivity as neither catalyst is effective without a ligand (entries 4 and 7). An alternative co-catalyst, sodium iodide,^13j,21^ was not as effective as Co(Pc) (entry 5 and Table S1†). The different products formed arise from different mechanisms of co-catalysis: Co(Pc) selectively forms benzyl radicals from BnOMs while NaI converts BnOMs to BnI which reacts similarly to benzyl bromide (Scheme 1B).

For convenience, the aryl halide, ligand, Co(Pc), and zinc could be added with the alcohol, Ms_2_O, and EtN(i-Pr)_2_ or after mesylate formation was complete with little difference in yield. However, if nickel was added at the beginning, rapid reduction or dimerization of the starting materials resulted.^20^

The nickel and cobalt co-catalytic strategy of synthesizing diarylmethanes was also successful when aryl iodides were employed as coupling partners. When benzyl mesylate was reacted with iodobenzene under the optimized reaction conditions, the diphenylmethane product was formed in good yield. Although product was formed in the absence of Co(Pc), the co-catalytic method improved yield and selectivity (Table 1, entries 9–12). Added NaI depressed the yield only slightly (entry 13 *vs.* entry 9), suggesting that PhI competes with *in situ* formed BnI more effectively than PhBr for oxidative addition to nickel (Scheme 1B).

**Scheme 2:**
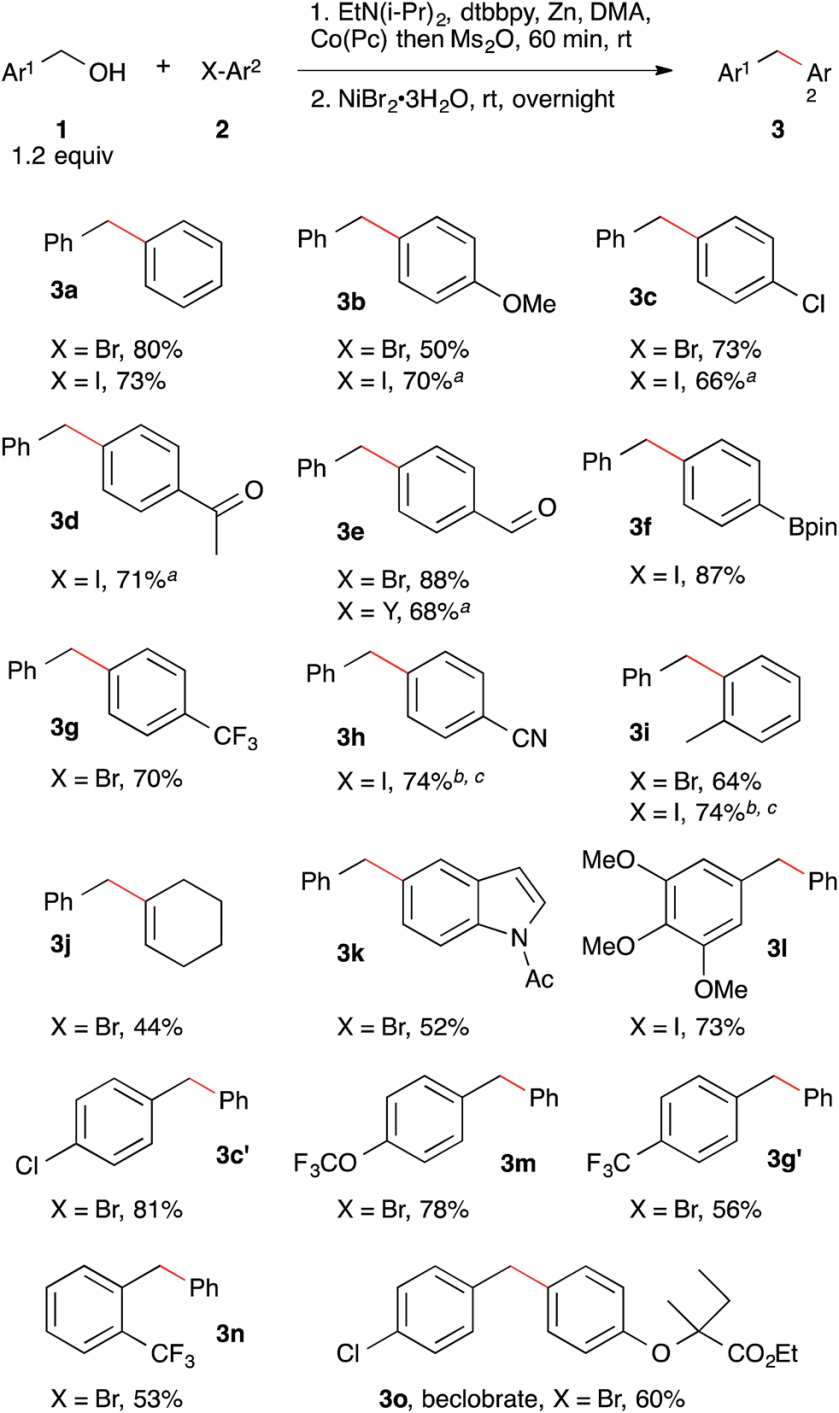
Nickel and cobalt co-catalyzed arylation of benzyl mesylates. Reactions performed as in Table 2, footnote *a* with 7 mol% NiBr_2_·3H_2_O, 5 mol% dtbbpy, and 1 mol% Co(Pc). Yield is isolated yield of purified product. See ESI† for details on reaction selectivity. ND = not detected. ^*a*^ No Co(Pc) was added to this reaction. ^*b*^ 3 mol% Co(Pc) was added to this reaction. ^*c*^ Reaction was run at 60 °C.

Application of these conditions to a variety of different aryl bromides, aryl iodides, and benzyl alcohols demonstrated the generality of the method (Scheme 2). The reaction tolerates functional groups, such as aldehyde (**3e**) and methyl ketone (**3d**), which are susceptible to the nucleophilic and basic character of some organometallic reagents. Additionally, a boronic acid ester was coupled without chemoselectivity problems, demonstrating complementarity with existing cross-coupling methods (**3f**).^13^

The coupling of aryl bromides and aryl iodides with benzyl mesylate provided comparable yields. In examples where the selectivity over bibenzyl was high but the yield was low, hydrodehalogenation of the arene was responsible for diminished yields.^20^ The aryl bromide conditions could be applied without further optimization to a vinyl bromide with reasonable success (**3j**). Both electron-rich and electron-poor benzyl alcohols couple effectively and steric hindrance on the benzyl alcohol did not result in lower yields (**3l**, **3c′**, **3m**, **3g′**, **3n**).^22^ Finally, we were able to apply this approach to the synthesis of beclobrate (**3o**), a diarylmethane compound that can be used to alter lipid levels in humans.^23^

The reactivity and selectivity of the reaction can also be rationally optimized. For example, the coupling of benzyl diethyl phosphate ester^24^ was sluggish under our standard conditions and starting materials were not consumed (Table 2). Presumably, this was due to the reduced leaving group ability of OP(O)(OEt)_2_ compared to OMs.^25^ Increasing the amount of Co(Pc) from 1 mol% to 6 mol% as well as increasing the temperature from rt to 80 °C allowed the reaction to proceed in high yield to form diphenylmethane (**3a′**).^22^ Notably, we observe no hydrodehalogenation under these conditions, perhaps because there is no acid (EtN(i-Pr)_2_·HOMs) present. We anticipate that a similar strategy could be employed to couple other less reactive electrophiles.

**Table 2:** Reaction with benzyl diethyl phosphate, a less reactive electrophile^*a*^

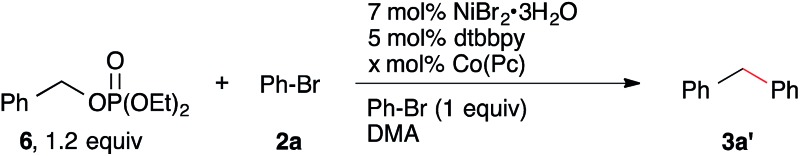			
Entry	Co(Pc) (mol %)	*T* (°C)	Yield^*b*^
1	3	rt	ND^*c*^
2	3	40	ND^*c*^
3	1	80	3^*c*^ ^,^ ^*d*^
4	3	80	36^*c*^ ^,^ ^*d*^
5	6	80	(70)^*d*^

^*a*^Reactions run at 0.25 M in DMA using pre-formed **6** for 15–22 h. See ESI.

^*b*^Yield of **3a′** from GC area% data. Yields in parentheses are isolated yields of purified product.

^*c*^Significant amounts of starting materials remain.

^*d*^Only byproduct observed by GC analysis was small amounts of benzene from hydrodehalogenation of Ph-Br.

**Scheme 3:**
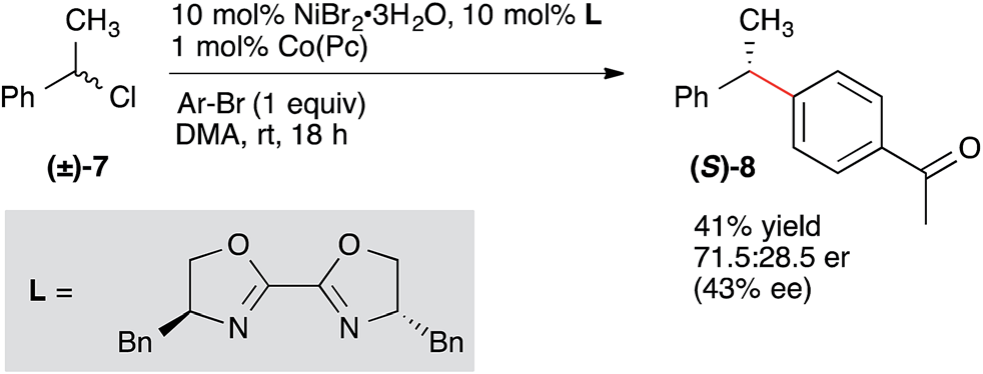
Preliminary enantioconvergent coupling of a secondary benzyl chloride with an aryl bromide. Configuration assigned by comparison of the optical rotation to those reported in the literature.

The likely intermediacy of a benzyl radical suggested to us that an enantioconvergent coupling of racemic secondary benzylic electrophile with an aryl bromide could be achieved.^26,27^ Although yields were low with secondary benzylic alcohol derivatives, α-chloroethylbenzene (**7**) coupled efficiently under slightly modified standard conditions to form diarylethane **8**(ref. 28) in 41% isolated yield and 43% ee (Scheme 3).^29^ This yield and enantioselectivity compares favorably to the results of Molander in the coupling of a racemic secondary benzylboron reagent with an aryl halide using the same chiral ligand (52% yield, 50% ee), suggesting a similar enantiodetermining step (radical capture by a chiral nickel complex).^7^ Together with the related highly enantioselective coupling of vinyl halides by Reisman,^16^ these results suggest highly enantioselective reactions to form diarylmethanes will soon be realized. Future studies will be aimed at improving the enantioselectivity and yield of these and related reactions.

## Conclusions

In conclusion, Co(Pc) is a new co-catalyst for radical generation that is compatible with nickel-catalysis offering a different selectivity than other established co-catalysts while maintaining high functional-group compatibility. The first synthesis of diarylmethanes from benzyl mesylates and aryl iodides or bromides is possible because (1) [Ni^0^] reacts with Ar–X faster than with BnOMs, preventing bibenzyl formation and (2) Co(Pc) reacts with benzyl mesylate faster than with Ar–X, selectively forming benzyl radicals. We propose that these benzyl radicals react with (L)Ni^II^(Ar)X to form product.^2,4,6,7^ These results demonstrate the potential of Co(Pc) as a nucleophilic co-catalyst for radical cross-coupling.
