# Correction: Cobalt co-catalysis for cross-electrophile coupling: diarylmethanes from benzyl mesylates and aryl halides

**DOI:** 10.1039/c5sc90021b

**Published:** 2015-04-24

**Authors:** Laura K. G. Ackerman, Lukiana L. Anka-Lufford, Marina Naodovic, Daniel J. Weix

**Affiliations:** a Department of Chemistry , University of Rochester , Rochester , NY 14627-0216 , USA . Email: daniel.weix@rochester.edu

## Abstract

Correction for ‘Cobalt co-catalysis for cross-electrophile coupling: diarylmethanes from benzyl mesylates and aryl halides’ by Laura K. G. Ackerman *et al.*, *Chem. Sci.*, 2015, **6**, 1115–1119.



## 

Figure 1 in our original article contained an error. The words oxidation and reduction were exchanged on entries 3 and 4. The corrected figure appears below.

**Fig. 1:**
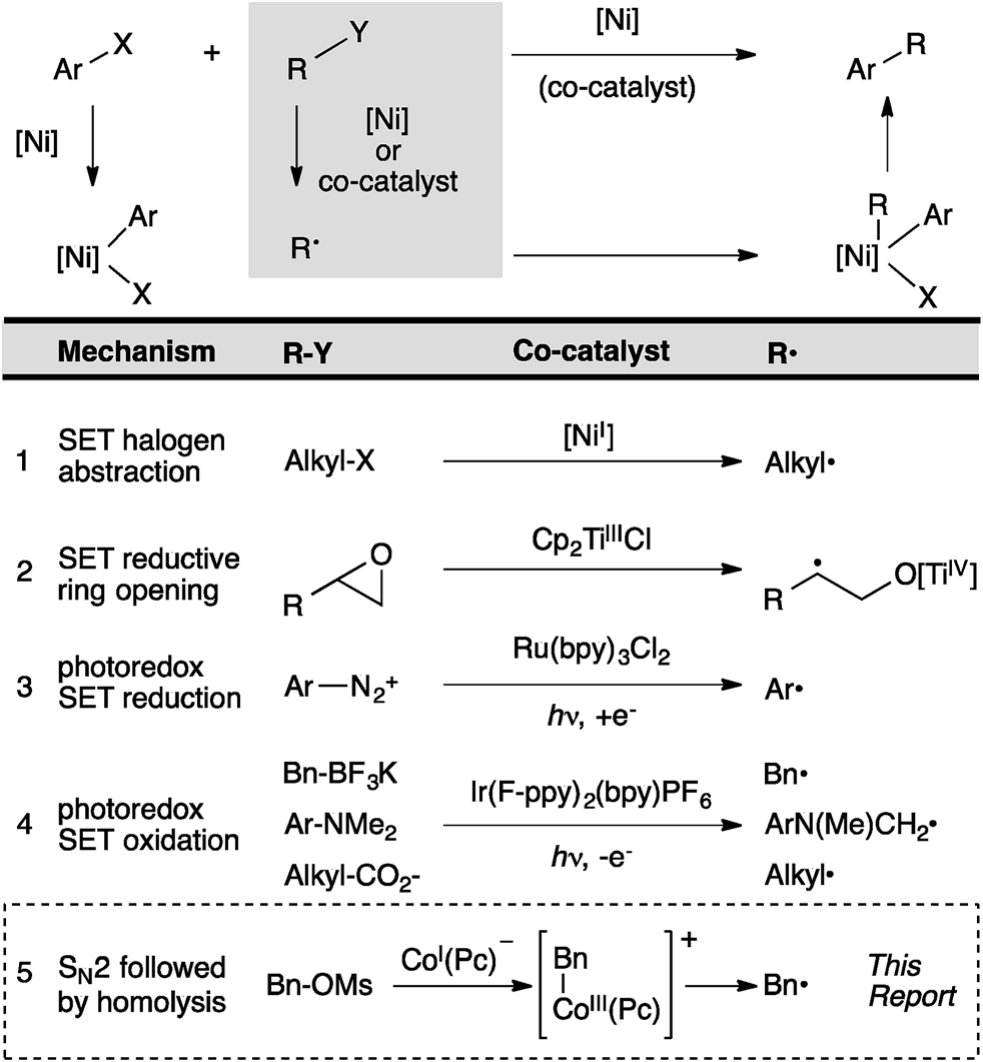
Comparison of radical co-generation methods in cross-coupling. An electrophile (Ar–X) reacts to form an arylmetal intermediate and the other substrate (R–Y) reacts to form a radical (R˙).

The Royal Society of Chemistry apologises for these errors and any consequent inconvenience to authors and readers.

