# Further correction: Cobalt co-catalysis for cross-electrophile coupling: diarylmethanes from benzyl mesylates and aryl halides

**DOI:** 10.1039/c6sc90082h

**Published:** 2016-12-16

**Authors:** Laura K. G. Ackerman, Lukiana L. Anka-Lufford, Marina Naodovic, Daniel J. Weix

**Affiliations:** a Department of Chemistry , University of Rochester , Rochester , NY 14627-0216 , USA . Email: daniel.weix@rochester.edu

## Abstract

Further correction for ‘Cobalt co-catalysis for cross-electrophile coupling: diarylmethanes from benzyl mesylates and aryl halides’ by Laura K. G. Ackerman *et al.*, *Chem. Sci.*, 2015, **6**, 1115–1119.



## 

A revised version of the Acknowledgements section, in which the acknowledgement to Sylvia Chen has been removed, is included below.

The Royal Society of Chemistry apologises for these errors and any consequent inconvenience to authors and readers.

