# Supplementary material for: Cobalt co-catalysis for cross-electrophile coupling: diarylmethanes from benzyl mesylates and aryl halides
Source: Chem Sci. 2014 Nov 10;6(2):1115–9. doi: 10.1039/c4sc03106g (PMC4323094; doi:10.1039/c4sc03106g)
Supplement: Supplementary file 1 [file SC-006-C4SC03106G-s001.pdf]

Electronic Supplementary Material (ESI) for Chemical Science.
This journal is © The Royal Society of Chemistry 2014

Cobalt co-catalysis for cross-electrophile coupling: diarylmethanes from benzyl mesylates and aryl halides

Laura K.G. Ackerman, Lukiana L. Anka-Lufford,
Marina Naodovic and Daniel J. Weix*.

Department of Chemistry, University of Rochester, Rochester, NY, USA 14627-0216.

SUPPORTING INFORMATION

Table of Contents

I. General Information S3
- A. Reagents S5
- B. Benchtop Techniques S5
- C. Analytical Techniques S5

II. Procedures S6
- A. Coupling of Aryl Halides with Benzyl Mesylates S6
- B. Coupling of Aryl Halides with Benzyl-X S6
- C. Monitoring Reaction by GC S6
- D. Purification of Compounds S7

III. Supplementary Table and Charts S7
- A. Reactivity of BnBr and BnOMs with Ni and Co **Table S1** S7
- B. Overview of Optimization **Figure S1** S8
- C. Optimization of the Reaction of Benzyl alcohol with Iodobenzene S9
  1. Nickel Precursors **Chart S1** S10
  2. Bases for *in situ* Formation of Benzyl Mesylate **Chart S2** S11
  3. Temperature **Chart S3** S12
  4. Reducing Agents **Chart S4** S13
- D. Optimization of the Reaction of Benzyl alcohol with Bromobenzene S14
  1. Co(Pc) Loading **Chart S5** S14
  2. $NiBr_2$•$3H_2O$ Loading (constant ligand loading) **Chart S6** S15
  3. Reducing Agents **Chart S7** S16
  4. Temperature **Chart S8** S17
  5. Stirring time for *in situ* Benzyl Mesylate Formation **Chart S9** S18
  6. Solvent **Chart S10** S19

IV. Characterization Data S20

V. Copies of NMR Spectra S26

I. General Information

A. Reagents

Nickel Sources: nickel(II) bromide trihydrate ($NiBr_2$•$3H_2O$, >98%), nickel(II) chloride ethylene glycol dimethyl ether ($NiCl_2$•dme), nickel(II) acetylacetonate ($Ni(acac)_2$), and nickel(II) iodide pentahydrate (NiI_2•$5H_2O$) were purchased from sigma Aldrich, stored in the glovebox, and used on the benchtop without exclusion of moisture or air. *Note: During periods of high humidity, it was noticeable that $NiBr_2$•$3H_2O$ picked up water when exposed to air for long periods of time. This "wet" $NiBr_2$•$3H_2O$ turned a light green color and should be avoided in conducting the reactions.*

Cobalt Sources: cobalt(II) chloride ($CoCl_2$, Aldrich) and cobalt(II) phthalocyanine (Co(Pc), Fluka, >97%) were used as received.

Ligands: 4,4'-di-*tert*-butyl-2,2'-dipyridine (dtbbpy), 2-acetylpyridine , N,N,N',N'-tetramethylethane-1,2-diamine (TMEDA), 2-(2-pyridyl)benzimidazole, bathophenanthroline (bathophen), 4,4',4''-tri-*tert*-butyl-2,2':6',2''-terpyridine (dtbtpy), tricyclohexylphosphine (PCy_3), 1,2-bis(diphenylphosphino)ethane (dppe) and 2,2′-Bis[(4S)-4-benzyl-2-oxazoline] were purchased from Sigma-Aldrich or Alfa Aesar and used as received. Ligands were handled on the benchtop without exclusion to moisture or air.

Reducing Agents: zinc dust (<10 μm, $\geq$98%) and manganese powder (-325 mesh, $\geq$99%) were purchased from Sigma Aldrich and stored in the glovebox. Zinc powder (median 6-9 micron, 97.5%) and manganese powder (-140 + 325 mesh, 99.6%) purchased from Alfa Aesar also gave high yields of cross coupled products. Reducing agents taken out of the glovebox were used on the benchtop and kept in the desiccator when not in use. *Zinc dust that was exposed to air and moisture for prolonged periods of time (weeks to months) resulted in diminished yields*.

Bases: triethylamine (Et_3N), *N,N*-diisopropylethylamine ($(i\text{-Pr})_2EtN$), pyrrolidine, and *N*-methylmorpholine were purchased from either Sigma Aldrich or Alfa Aesar and used as received. After storing on the benchtop for a longer period of time, the amine reagents were purified by distillation from calcium hydride (reagent grade, 95%) and stored over potassium hydroxide. Potassium carbonate (K_2CO_3, J. T. Baker) and tripotassium phosphate (K_3PO_4, Sigma Aldrich) were used as received.

Ms_2O: methanesulfonic anhydride was purchased from Alfa Aesar and was stored in a desiccator over $CaSO_4$. The anhydride was handled on the benchtop and care was taken to minimize exposure to air. Anhydride that was exposed to ambient moisture provided consistently good results for up to ten days.

Aryl halides: Aryl iodides, such as iodobenzene, 4-iodoanisole, 4-iodoacetophenone, 4-iodobenzaldehyde, 4-iodo-1-chlorobenzene, 4-iodobenzonitrile, and 2-iodotoluene were purchased from commercial suppliers and used as received. Likewise, aryl bromides such as bromobenzene, 4-bromoanisole, 4-bromobenzaldehyde, and 4-bromobenzotrifluride were also purchased. All other aryl iodides and bromides such as 4-iodophenylboronic acid pinacol ester[1], cyclohexenyl bromide[2] and N-acetyl-5-bromoindole[3] substrates were prepared according to the reported procedure. Liquid aryl halides, when visibly discolored from their typical colorless appearance, were purified either by distillation or by filtration through a short (2.0 cm) plug of basic aluminum oxide in a Pasteur pipette.

---

[1] Aquino, M.; Guerrero, M. D.; Bruno, I.; Terencio, M. C.; Paya, M.; Riccio, R. *Biorg. Med. Chem.* **2008**, *16*, 9056.
[2] Zhan, F.; Liang, G. *Angew. Chem. Int. Ed.* **2013**, *52*, 1266-1269.
[3] Phipps, R.J.; Brimster, N. P.; Gaunt, M J. *J. Am. Chem. Soc.* **2008,** 130, 8172.

Ethyl 2-(4-bromophenoxy)butanoate. In a round-bottomed flask fitted with a stir bar, at room temperature, and under a nitrogen atmosphere, 4-bromophenol (2.0 g, 12 mmol) in 12 mL of *N,N*-dimethylformamide (DMF) was added dropwise to sodium hydride (0.3 g, 13 mmol) in 12 mL of DMF. After mixing for 45 minutes, ethyl 2-bromobutanoate (2.3 g, 12 mmol) in 12 mL of DMF was added dropwise, and was followed with the addition of sodium iodide (2.1 g, 14 mmol). The reaction mixture was then brought to reflux (80 °C bath temperature) for 3 h. The reaction mixture was allowed to cool to room temperature, diluted with H_2O (20 mL), and extracted with EtOAc (3 × 20 mL). The combined organic layers were washed with H_2O (2 × 10 mL) and then the organic layer was dried over Na_2SO_4, the drying agent was removed by filtration, and mixture was concentrated under reduced pressure. The crude material was purified by silica gel chromatography eluting with 1:1 hexanes/EtOAc to yield (1.9 g, 56% yield) of a colorless oil. ^{1}H-NMR (500 MHz; $CDCl_3$): δ 7.39 (d, *J* = 9.0 Hz, 2H), 6.79 (d, *J* = 9.0 Hz, 2H), 4.53 (t, *J* = 6.2 Hz, 1H), 4.24 (q, *J* = 7.1 Hz, 2H), 2.01 (quintet, *J* = 7.1 Hz, 2H), 1.27 (t, *J* = 7.1 Hz, 4H), 1.10 (t, *J* = 7.4 Hz, 3H). ^{13}C NMR (126 MHz; $CDCl_3$): δ 172.0, 157.8, 133.1, 117.7, 114.5, 78.7, 62.0, 26.8, 14.9, 10.3. GC-MS (EI+) *m/z* (% rel. int., ion): GC-MS (EI+) *m/z* (% rel. int., ion): 287.90 (35.85, M^+), 171.90 (100.00, M^+-$C_6H_{11}O_2$).

Ethyl 2-(4-bromophenoxy)-2-methylbutanoate. To a well stirred solution of LDA [prepared from n-butyllithium (1.00 mL of 2.25 M solution in hexanes, 2.25 mmol) and diisopropylamine (0.32 mL, 2.3 mmol)] at -78 °C under nitrogen atmosphere, a solution of ethyl 2-(4-bromophenoxy)butanoate (500 mg, 1.74 mmol) in dry THF (6 mL) was added dropwise. The reaction was then stirred for 4 h, during which time the reaction was allowed to slowly warm to 0 °C. The reaction was then recooled to -78 °C and a solution of methyl iodide (0.15 mL, 2.4 mmol) in dry THF (6 mL) was added dropwise. The resulting mixture was allowed to slowly warm to rt overnight. When judged complete by GC analysis, the reaction mixture was quenched with saturated NH_4Cl_{aq} and extracted with ether (3 × 15 mL). The combined ether layers washed with water (2 × 5 mL) and brine (2 × 5 mL), then dried over Na_2SO_4. After removal of the drying agent by filtration, the solvent was removed under reduced pressure. The crude material was purified by silica gel chromatography (1:1 hexanes/EtOAc) to yield (350 mg, 66% yield) of a colorless oil. ^{1}H-NMR (500 MHz; $CDCl_3$): δ 7.36-7.34 (m, 2H), 6.77-6.75 (m, 2H), 4.26 (q, *J* = 7.1 Hz, 2H), 2.05-1.94 (m, 2H), 1.51 (s, 3H), 1.28 (t, *J* = 7.1 Hz, 3H), 1.00 (d, *J* = 7.5 Hz, 3H). ^{13}C NMR (126 MHz; $CDCl_3$): δ 173.8, 154.8, 132.1, 121.0, 114.6, 82.4, 61.5, 32.6, 20.9, 14.3, 7.9. GC-MS (EI+) *m/z* (% rel. int., ion): 301.90 (79.18, M^+), 173.90 (100.00, M^+-$C_7H_{13}O_2$).

Benzyl alcohols: Benzyl alcohol, 2,3,4-trimethoxybenzylalcohol, *p*-chlorobenzylalcohol, *p*-trifluoromethylbenzylalcohol, *p*-trifluoromethoxybenzylalcohol, and *o*-trifluoromethylbenzylalcohol, were either purchased from Sigma Aldrich or prepared by the reduction of the corresponding aldehydes with $NaBH_4$. Liquid alcohols were washed with 10% KOH (*aq*) solution and distilled under reduced pressure. Solid benzyl alcohols were purified by column chromatography with silica gel.

Benzyl-X: α-chloroethylbenzene was purchased from TCI. Benzyldiethylphosphate was synthesized according to the literature procedure.[4]

Solvents: 1,3-dimethyl-3, 4, 5, 6-tetrahydro-2(1H)-pyrimidinone (DMPU) and 1-methyl-2-pyrrolidinone (NMP) were purchased from Aldrich and used without further purification. *N,N*-dimethylacetamide (DMAc), *N,N*-dimethylformamide (DMF) tetrahydrofuran, benzene, and dichloromethane were prepared from ACS grade, inhibitor free solvents by treatment with activated alumina and molecular sieves in a solvent purification system. Water contents in solvents were routinely monitored and measured with a Metrohm Karl-Fischer apparatus and were less than 30 ppm in all cases.

---

[4] McLaughlin, M. *Org. Lett.* **2005**, 7, 4875-4878.

B. Benchtop Techniques

Nickel-catalyzed reductive cross coupling reactions can be slightly sensitive to oxygen and moisture. Excess moisture from reagents or reducing agents that have been exposed to air for long periods of time can prolong reaction completion and lead to erosion of cross selectivity. While aware of this propensity, and taking care to use dry solvents, freshly distilled reagents, and oven-dried glassware, the reactions were assembled on the benchtop without any special precautions to minimize exposure to air. After drying 1 dram vials equipped with teflon stir bars (Fisher Scientific, 2 mm diameter x 7 mm length) in a 150 °C oven and cooling them under nitrogen to ambient temperature, the following procedure was used on the bench-top: Solids were weighed out and added directly into the vials. Liquid reagents were then added by automated delivery pipets, except for aryl bromides and the substrate 4-iodophenylboronic acid pinacol ester, which were added with the catalyst and co-catalyst after benzyl mesylate formation (Section II.A). *We recommend that this later procedure be used for aryl halides with sensitive functional groups.* Methanesulfonic anhydride was added as quickly as possible to the vials before capping them with PTFE-faced silicone septa and stirring them on a well plate at room temperature. *Note that an exothermic reaction occurs upon addition of Ms_2O and the resulting mixture will feel warm to the touch.* The order of the addition of the reagents, particularly that of the addition of the catalyst and co-catalyst should be followed precisely as presented in section II.A. This is critical for the cross selectivity of the reaction to be preserved. If the nickel catalyst is added during the generation of the benzyl mesylate, it will result in the rapid reduction and/or dimerization of the starting materials.

C. Analytical Techniques

^{1}H nuclear magnetic resonance (NMR) spectroscopy chemical shifts are reported in ppm and referenced to TMS (tetramethylsilane) in $CDCl_3$ (δ= 0 ppm). For ^{13}C NMR and ^{19}F NMR chemical shifts, the residual solvent peak ($CDCl_3$, δ= 77.0 ppm) and the external standard, α,α,α–trifluorotoluene (δ= 0 ppm) were used as references. NMR spectra were recorded on Avance Bruker NMR spectrometers operating at either 400.13 MHz or 500.13 MHz and data analysis was performed using the iNMR software package (www.inmr.net). Chemical shifts are reported in parts per million (ppm), multiplicities are indicated by s (singlet), d (doublet), t (triplet), q (quartet), m (multiplet) and br (broad). Coupling constants, *J*, are reported in Hertz.

Gas Chromatography (GC) analyses were carried out on an Agilent 7890A GC equipped with dual DB-5 columns (20 m x 1180 μm 0.18 μm), dual FID detectors, and using hydrogen as a carrier gas. The GC analysis method used in all cases was: 1 μL injection of sample with an injection temperature of 300 °C, 100:1 split ratio, and an initial inlet pressure of 20.3 psi. Throughout the run, the pressure was maintained at 1.8 mL/min. The initial oven temperature was held for 0.46 min at 50 °C and increased at 65 °C/min rate until reaching 300 °C. Finally, the temperature was held at 300 °C for 0.69 min. The average run time was 5 minutes with an FID temperature of 325 °C.

Gas Chromatography/Mass Spectrometry (GC/MS) analyses were performed on a Shimadzu GCMS-QP2010 equipped with an RTX-XLB column (30 m x 0.25 mm x 0.28 μm) with a quadrupole mass analyzer, using helium as a carrier gas. The analysis method used in all cases was: 5 μL injection volume of a sample, with an injection temperature of 225 °C, 25:1 split ratio, and an initial inlet pressure of 7.81 psi. Throughout the run, the pressure was held constant at 1.0 mL/min, the interface temperature was held at 250 °C, and the ion source (EI+, 30 eV) was held at 250 °C. The initial oven temperature for the sample was held at 50 °C for 3 minutes and then increased by 40 °C/min until reaching 280 °C. Finally, the temperature was held at 280 °C for 3 min. The average run time was 11.75 minutes.

High resolution mass spectrometry (HRMS) analyses were performed at the University of Illinois MS facility on a Waters Micromass 70-VSE instrument operating in EI+ mode while attached to a GC/MS. This instrument has a resolution of >50,000, resulting in resolution of differences in mass down to 0.003 to 0.005 *m*/*z* for the compounds synthesized. The 70-VSE mass spectrometer was purchased in part with a grant from the Division of Research Resources, National Institutes of Health (RR 04648).

Chromatography purification was carried out with silica gel (EMD, silica gel 60, particle size 0.040-0.063 mm) using standard flash chromatography techniques. Analytical thin layer chromatography (TLC) was performed on silica gel glass plates (EMD, 60 F254; 250 μm). Visualization was accomplished with UV light, or treatment with a phosphomolybdic acid ethanol solution, aqueous cerium ammonium molybdate or potassium permanganate solution, followed by heating.

II. Procedures

A. Coupling of Aryl Halides with Benzyl Mesylates. To a 1-dram vial equipped with a stir bar was placed activated zinc (65 mg, 1.0 mmol, 2.0 equiv), dtbbpy (6.70 mg, 0.025 mmol, 0.050 equiv), dodecane (10 μL, 0.044 mmol), benzyl alcohol (62 μL, 0.60 mmol, 1.2 equiv), aryl halide (0.50 mmol, 1.0 equiv), DIPEA (139 μL, 0.800 mmol, 1.60 equiv), and DMA (2.0 mL). The mixture was stirred for 5 minutes at room temperature after which methanesulfonic anhydride (125 mg, 0.720 mmol, 1.40 equiv) was added. The contents were stirred for 60 min at room temperature, followed by the addition of $NiBr_2{\cdot}3H_2O$ (9.50 mg, 0.035 mmol, 0.070 equiv) and either 1 mol% (2.90 mg, 0.005 mmol, 0.010 equiv) or 3 mol% (8.60 mg, 0.015 mmol, 0.030 equiv) of Co(Pc). *When 4-iodophenylboronic acid pinacol ester or aryl bromides were employed, these reagents were added last, just prior to catalyst and co-catalyst addition.* The reaction was then stirred either at room temperature or at 60 °C and the reaction progress was monitored by GC. Control reactions as well as optimization experiments also followed this general procedure, by either omitting or changing specified reagents.

B. Coupling of Aryl Halides with Benzyl-X (Benzyldiethylphosphate and α-chloroethylbenzene. To a 1-dram vial equipped with a stir bar was placed activated zinc (65 mg, 1.0 mmol, 2.0 equiv), either dtbbpy (6.70 mg, 0.025 mmol, 0.050 equiv) or 2,2′-Bis[(4S)-4-benzyl-2-oxazoline] (16 mg, 0.05 mmol, 0.10 equiv), dodecane (10 μL, 0.044 mmol), benzyl-X (0.60 mmol, 1.2 equiv), aryl halide (0.50 mmol, 1.0 equiv), either 7 mol% (9.50 mg, 0.035 mmol, 0.070 equiv) or 10 mol% (13.6 mg, 0.050 mmol, 0.100 equiv) of $NiBr_2{\cdot}3H_2O$, either 1 mol% (2.90 mg, 0.005 mmol, 0.010 equiv) or 6 mol% (17.4 mg, 0.010 mmol, 0.060 equiv) of Co(Pc) and DMA (2.0 mL). The reaction was then stirred either at room temperature or at 80 °C and the reaction progress was monitored by GC. The coupling reaction of aryl halide with α-chloroethylbenzene using chiral ligand 2,2′-Bis[(4S)-4-benzyl-2-oxazoline] was setup in a glovebox.

C. Monitoring Reactions by GC. Reaction progress and completion was monitored by GC analysis, by taking 50 μL aliquots of the crude reaction mixture with a gas-tight syringe at specified time points. The samples were prepared by quenching the aliquot in a 1-dram vial with 100 μL of water. The resulting mixture was diluted with diethylether, mixed, and filtered through a 2-cm silica plug in a Pasteur pipette directly into a GC vial. GC yields were determined based on the area percent of the analyte. For quantitative analysis the area percent of the analyte was compared to the signal corresponding to a known amount of dodecane and percent yield was calculated based on the known amount of starting material.

D. Purification. Purification was accomplished by filtering the reaction mixture through a 2-cm silica plug, which assisted in the removal of remaining nickel and zinc residue. The top of the silica plug was rinsed with small portions of ether. The resulting filtrate was diluted with diethylether or DCM and washed with 3 x 20 mL of water, after which the organic layer was separated from the aqueous layer. The aqueous layer was then washed with 2 x 20 mL of diethylether or DCM, and the organic layers were combined and concentrated *in vacuo*. To the crude product was added approximately 10 mL of dichloromethane, to which a minimal amount of silica was also added before concentrating the mixture *in vacuo* once more. This product-loaded silica was then used for flash chromatography.

III. Supplementary Tables and Charts

Table S1. Reactivity of benzyl electrophiles with cobalt and nickel under reducing conditions.

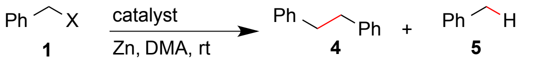

Entry[a]	X	Catalyst[b] [c]	t (min)	4[d]	5
1	Br	[Ni] only	60	76	6
2	OMs	[Ni] only	480	2	8
3	Br	Co(Pc) only	120	18	57
4	OMs	Co(Pc) only	480	4	42
5	Br	[Ni] + Co(Pc)	60	84	8
6	OMs	[Ni] + Co(Pc)	480	10	52
7[e]	OMs	[Ni] + Co(Pc)	480	51	25

[a] Reactions run on 0.5 mmol scale in 2 mL of *N,N*-dimethylacetamide (DMA). Benzyl mesylate was formed *in situ* from benzyl alcohol. [b] [Ni] = 7 mol% $NiBr_2{\bullet}3H_2O$ and 5 mol% 4,4'-di-*tert*-butyl-2,2'-bipyridine (dtbbpy). [c] Co(Pc) = 1 mol% Cobalt phthalocyanine. [d] Yields reported as GC Area%. [e] 25 mol% NaI was added.

Selectivity Data for the Optimization of the Synthesis of Diarylmethanes

B. Overview of Optimization

Initial conditions for the reaction of benzyl alcohols with aryl halides were derived from earlier nickel-catalyzed cross coupling reactions.[5] Previous methodologies employed zinc or manganese as a reducing agent, the use of soluble nickel precursors, and polar aprotic solvents for the stabilization of the catalyst as well as effective radical generation. At the beginning of the optimization process, zinc was chosen as a suitable reducing agent while DMA was chosen as an appropriate solvent to use for the exploration of reaction conditions.

For the specific reaction of benzyl mesylate with iodobenzene, the stoichiometry of the reagents, temperature, and solvents were studied. While the preliminary reaction of pre-formed benzyl mesylate with PhI was successful, the instability of the benzyl mesylate as a starting material, and the wide availability of benzyl alcohols, motivated us to design an *in situ* procedure for the benzyl substrate. As presented in the charts in sections III.B and III.C, the benzyl mesylate could be conveniently accessed by adding an excess of base to benzyl alcohol followed by Ms_2O. At least 20 minutes of stirring after the addition of Ms_2O was needed to afford the diphenylmethane target in good yield. The final set of optimized reaction conditions for both aryl iodides and aryl bromides is included below. Charts S1-S12 in III.B and III.C. summarize the relevant optimization reactions of this methodology and the selectivity for each set of conditions.

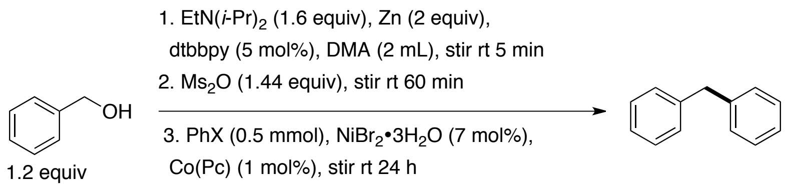

During the course of the reaction between benzyl alcohols and aryl halides under reducing conditions, several major byproducts were formed in addition to the desired diphenylmethane target: bibenzyl, biphenyl, toluene, and benzene. Based upon our mechanistic hypothesis (Scheme 1), we propose that these byproducts can be generated via the pathways shown in Figure S1. The blue pathway represents diphenylmethane formation via capture of a cobalt-generated benzyl radical by arylnickel(II) (step e) and reductive elimination to form product (step h). Toluene formation could be the result of either protonation of a benzylcobalt intermediate (step c) or hydrogen-atom abstraction by a benzyl radical (step d). Benzene formation would arise from protodearylation of arylnickel (II) (step g), while biphenyl would arise from disproportionation arylnickel(II) (step k) and reductive elimination (step l). Similarly, bibenzyl could arise from a disproportionation and reductive elimination sequence (step j), if nickel(0) reacted with a benzyl electrophile instead of an aryl electrophile (step i).

The pathways by which toluene (yellow box) and benzene (orange box) form are in direct competition with the diphenylmethane-forming pathway: when (dtbbpy)Ni(Ar)(X) is present for too long in solution without the presence of the benzyl radical partner, protonation will occur to form benzene (orange box). Likewise, when the concentration of benzyl radical is too high, hydrogen-atom abstraction from solvent, substrate, or product may occur rather than reaction with the arylnickel(II) complex (yellow box) or recombination may form bibenzyl (not shown). Slow reaction of (dtbbpy)Ni(Ar)(X) with a radical (step e) could lead to the formation of biphenyl through disproportionation (green box). Finally, formation of benzyl bromide or iodide could lead to a benzylnickel(II) intermediate (step i) and the formation of bibenzyl (grey box). In order to achieve high cross selectivity, the rates of formation of the two activated coupling partners, arylnickel(II) and benzyl radical, need to be well-matched.

---

[5] (a) Everson, Daniel A.; Shrestha, Ruja; Weix, Daniel J. *J. Am. Chem. Soc.* **2010**, *132*, 920-921; (b) Everson, Daniel A.; Jones, Brittany A.; Weix, Daniel J. *J. Am. Chem. Soc.* **2012**, *134*, 6146-6159.

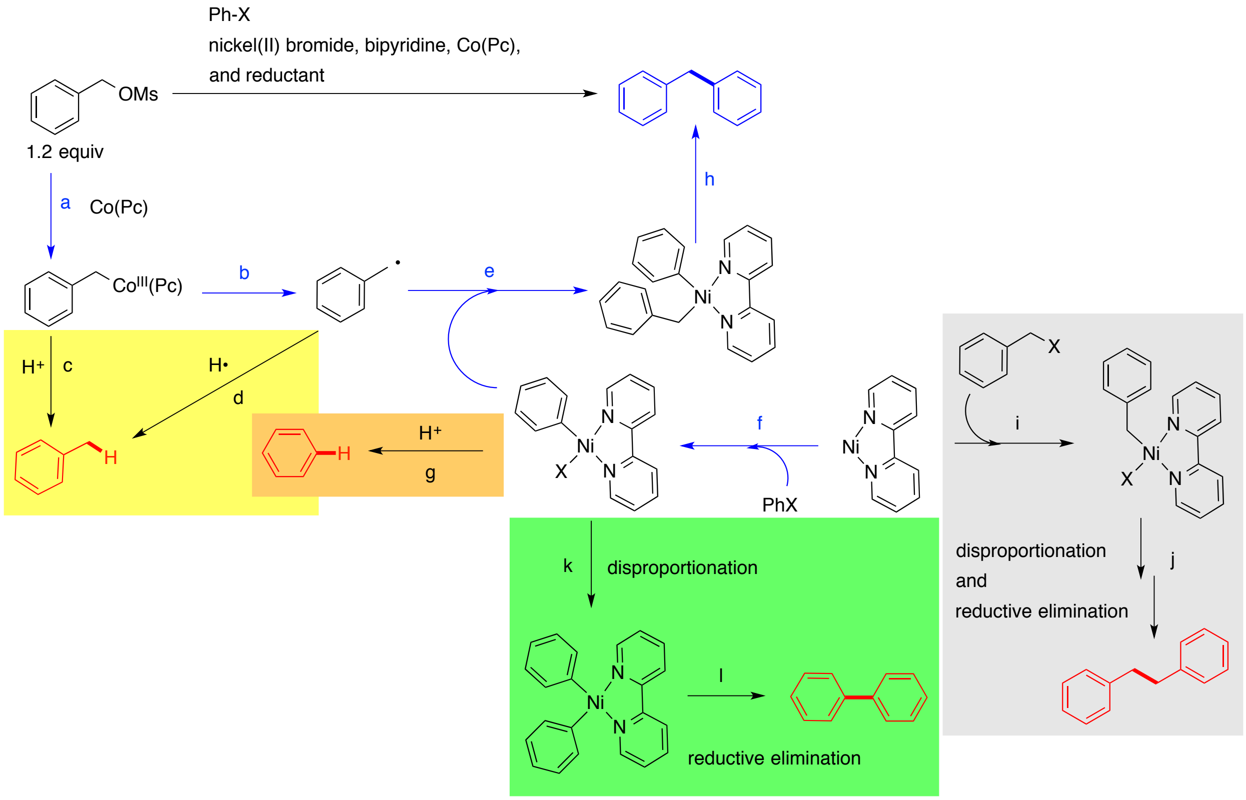

Figure S1. Proposed mechanism for product and byproduct formation. Blue arrows indicate the pathway by which product is formed (a, b, e, f, and h). Benzene (orange box), bibenzyl (grey box), biphenyl (green box), and toluene (yellow box) are formed off-cycle.

C. Optimization of the Reaction of Benzyl Alcohol with Iodobenzene

For the reactions depicted below, conditions that differ from the general reaction conditions are highlighted in bold. All of the bar charts are expressed as GC Area% analyzed after 24 h. In some cases the reactions were not complete, however, data at 24 h was included for comparison. In the majority of experiments 5-15% of the mass is not accounted for in the graph and is attributed to benzyl alcohol and/or benzaldehyde formation.

1.Nickel Precursors

Chart S1. Selectivity Data for Selected Nickel(II) Precursors.

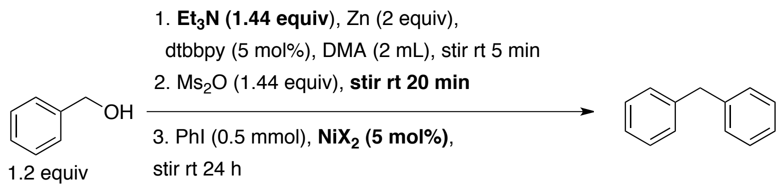

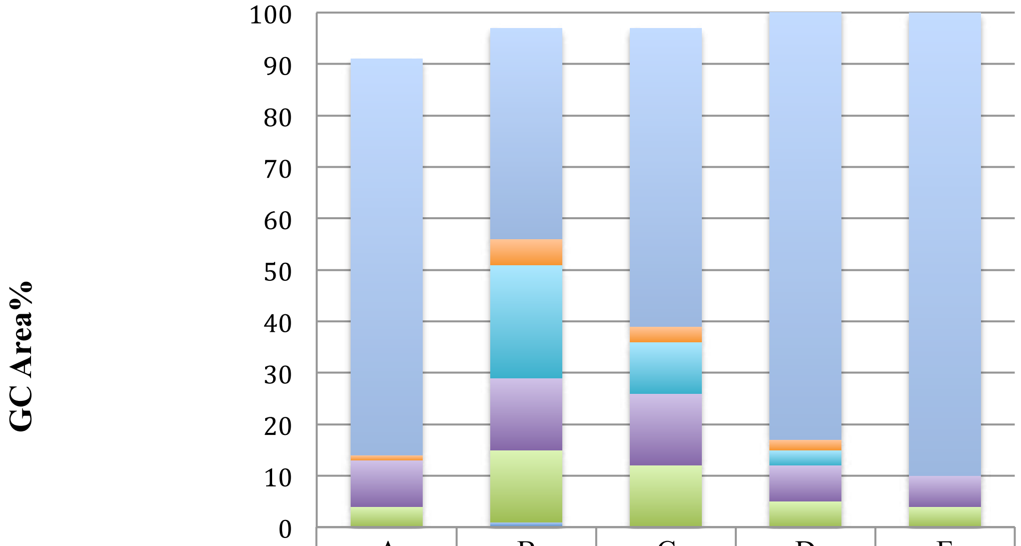

	A	B	C	D	E
Product A%	77	41	58	84	90
Biphenyl A%	1	5	3	2	0
Bibenzyl A%	0	22	10	3	0
Benzene A%	9	14	14	7	6
Toluene A%	4	14	12	5	4
Iodobenzene A%	0	0	0	0	0
Benzyl Mesylate A%	0	1	0	0	0

Code	Nickel Precursor
A	$NiCl_2$(dme)
B	Ni(acac)$_2$
C	NiI_2•5H_2O
D	$NiBr_2$•3H_2O
E	$NiBr_2$(dme)

2. Bases for *in situ* Formation of Benzyl Mesylate

Chart S2. Selectivity Data for Bases Used in Benzyl Mesylate Formation.

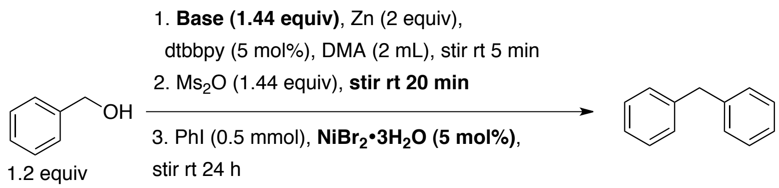

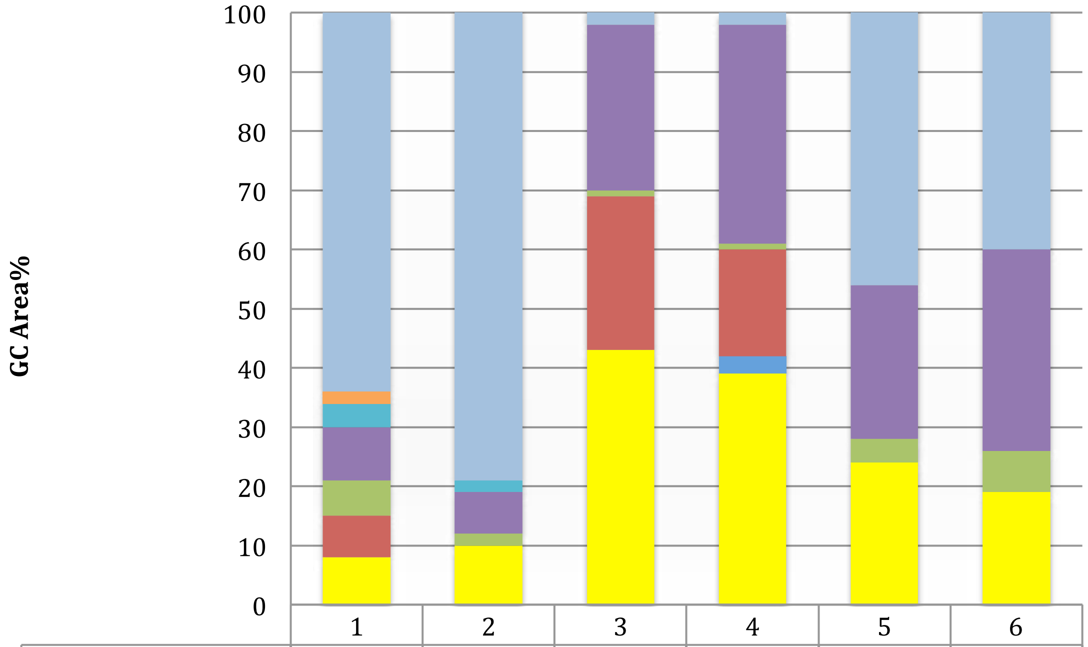

	1	2	3	4	5	6
Product A%	64	80	3	2	48	41
Biphenyl A%	2	0	0	0	0	0
Bibenzyl A%	4	2	0	0	0	0
Benzene A%	9	7	28	37	26	34
Toluene A%	6	2	1	1	4	7
Iodobenzene A%	7	0	26	18	0	0
Benzyl Mesylate A%	0	0	0	3	0	0
Benzyl Alcohol A%	8	10	43	39	24	19

Base Key

Code	Base
1	Triethylamine
2	*N,N*-diisopropylethylamine
3	Potassium carbonate
4	Tripotassiumphosphate
5	Pyrrolidine
6	*N*-methylmorpholine

3. Temperature

Chart S3. Selectivity Data for Varying Temperatures (with PhI).

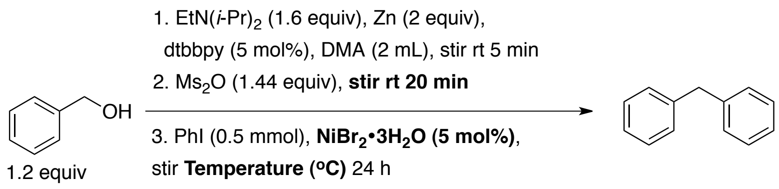

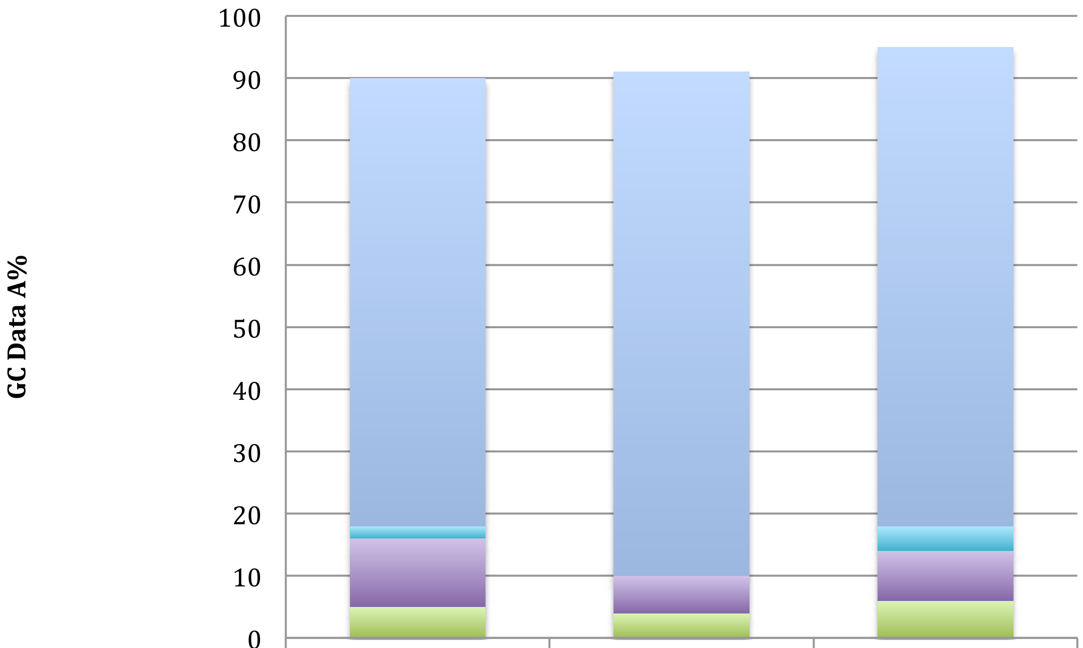

	40	60	80
Product A%	72	81	77
Biphenyl A%	0	0	0
Bibenzyl A%	2	0	4
Benzene A%	11	6	8
Toluene A%	5	4	6
Iodobenzene A%	0	0	0
Benzyl Mesylate A%	0	0	0

4. Reducing Agents

Chart S4. Selectivity Data for Alternate Reducing Agents (with PhI).

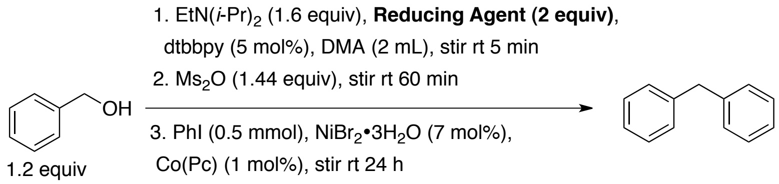

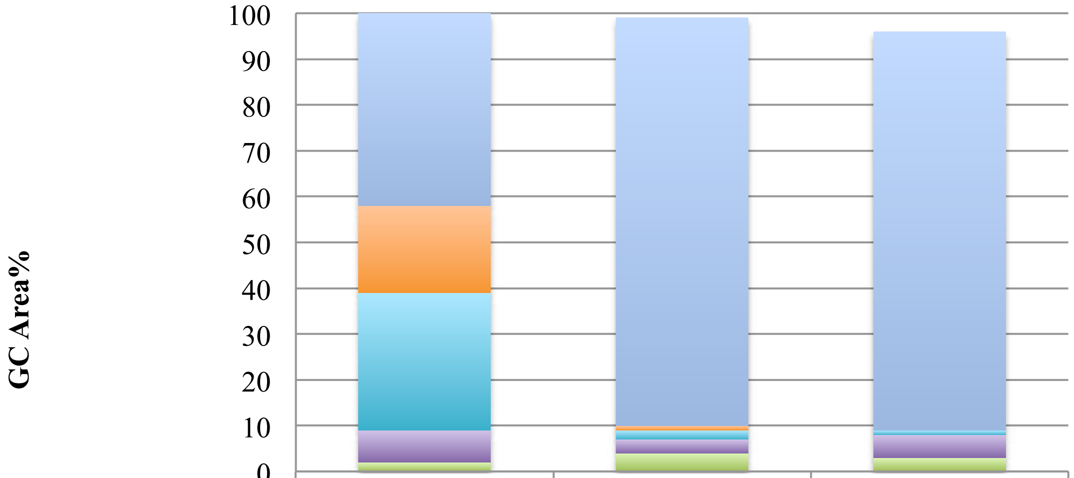

	Mn powder	Zn dust	Activated Zn dust
Product A%	42	89	87
Biphenyl A%	19	1	0
Bibenzyl A%	30	2	1
Benzene A%	7	3	5
Toluene A%	2	4	3
Iodobenzene A%	0	0	0
Benzyl Mesylate A%	0	0	0

Key:
"Activated Zn dust" refers to zinc dust that was washed with a 2% aqueous HCl solution, filtered, washed with water, EtOH, and ether, and subsequently dried in a vacuum oven.

D. Optimization of the Reaction of Benzyl alcohol with Bromobenzene

1. Co(Pc) Loading

Chart S5. Selectivity Data for Co(Pc) Co-Catalyst Loading.

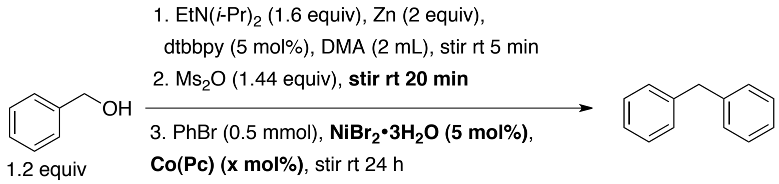

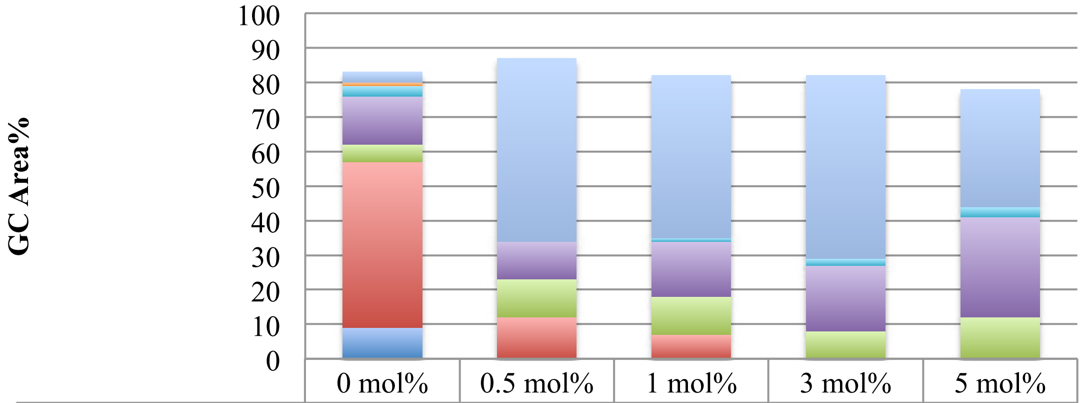

	0 mol%	0.5 mol%	1 mol%	3 mol%	5 mol%
Product A%	3	53	47	53	34
Biphenyl A%	1	0	0	0	0
Bibenzyl A%	3	0	1	2	3
Benzene A%	14	11	16	19	29
Toluene A%	5	11	11	8	12
Bromo Benzene A%	48	12	7	0	0
Benzyl Mesylate A%	9	0	0	0	0

2. $NiBr_2$•$3H_2O$ Loading (with dtbbpy loading constant at 5 mol%)

Chart S6. Selectivity Data for $NiBr_2$•$3H_2O$ Loading with 5 mol% dtbbpy.

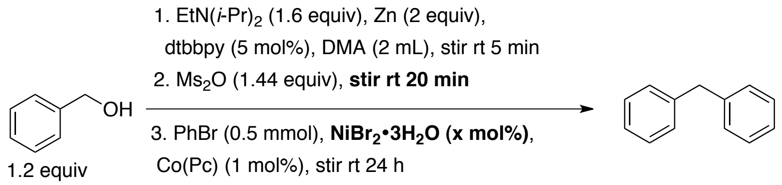

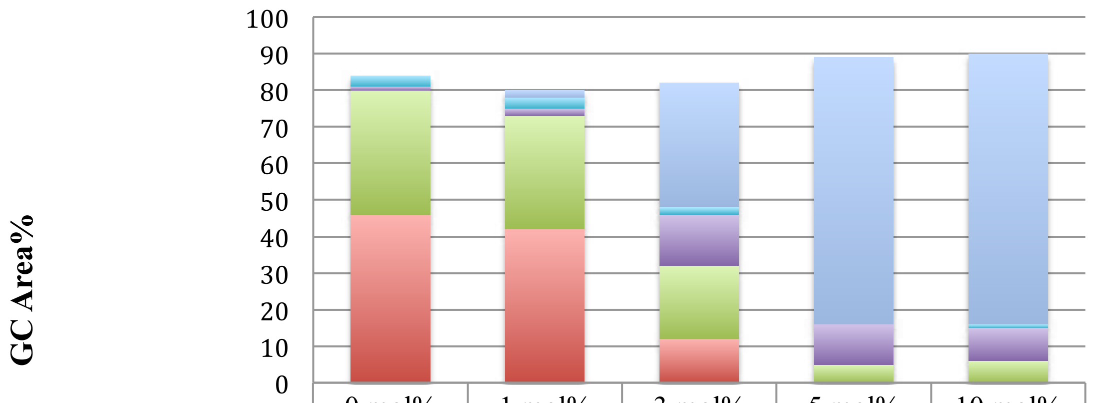

	0 mol%	1 mol%	3 mol%	5 mol%	10 mol%
Product A%	0	2	34	73	74
Biphenyl A%	0	0	0	0	0
Bibenzyl A%	3	3	2	0	1
Benzene A%	1	2	14	11	9
Toluene A%	34	31	20	5	6
Bromo Benzene A%	46	42	12	0	0
Benzyl Mesylate A%	0	0	0	0	0

3. Reducing Agent

Chart S7. Selectivity Data for Alternate Reducing Agents (with PhBr).

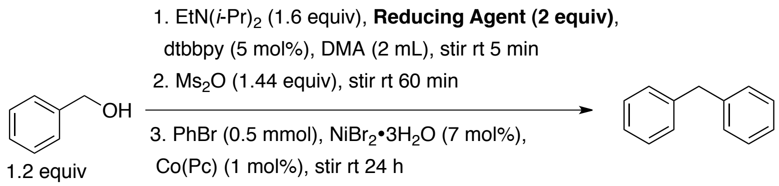

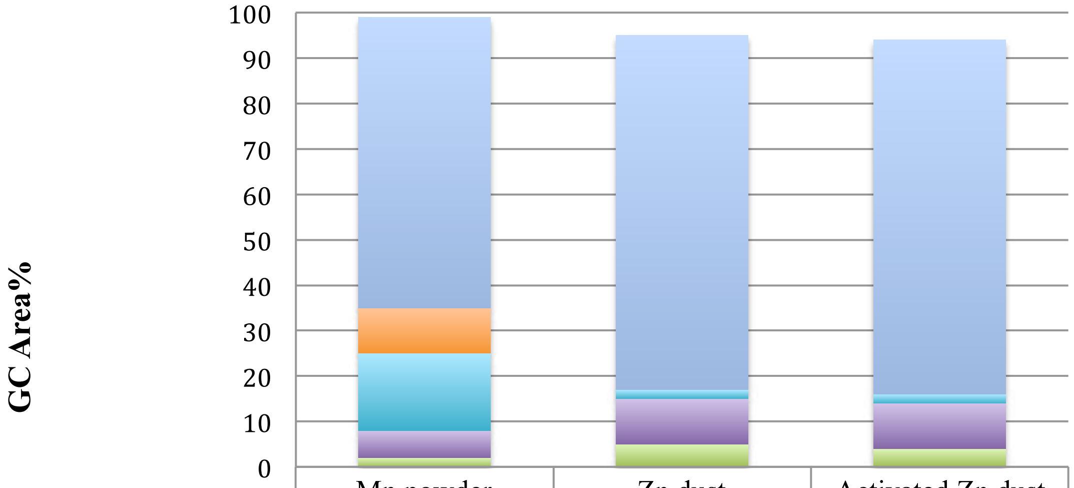

	Mn powder	Zn dust	Activated Zn dust
Product A%	64	78	78
Biphenyl A%	10	0	0
Bibenzyl A%	17	2	2
Benzene A%	6	10	10
Toluene A%	2	5	4
Bromo Benzene A%	0	0	0
Benzyl Mesylate A%	0	0	0

Key:
"Activated Zn dust" refers to zinc dust that was washed with a 2% aqueous HCl solution, filtered, washed with water, EtOH, and ether, and subsequently dried in a vacuum oven.

4. Temperature

Chart S8. Selectivity Data for Varying Temperatures (with PhBr).

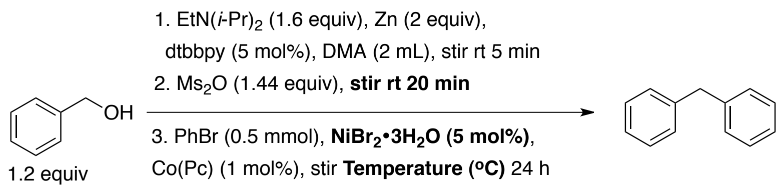

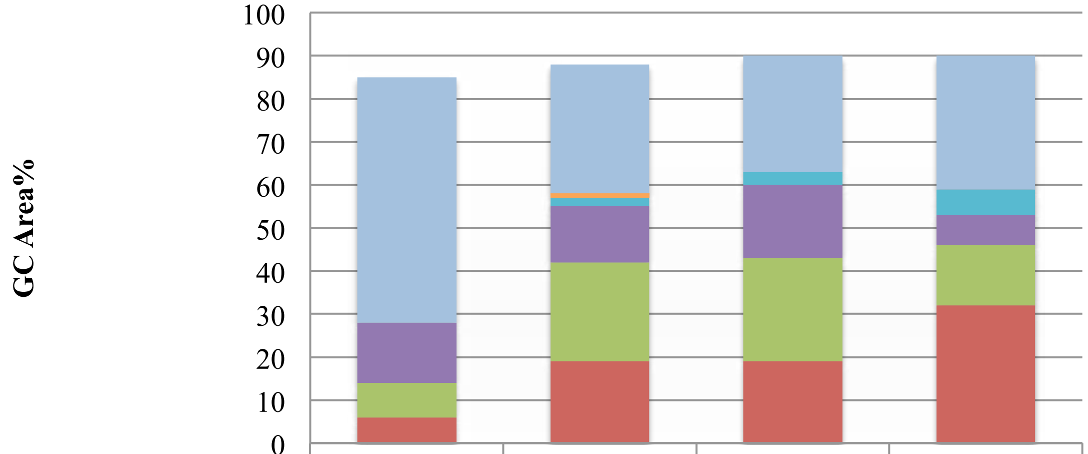

	23	40	60	80
Product A%	57	30	27	31
Biphenyl A%	0	1	0	0
Bibenzyl A%	0	2	3	6
Benzene A%	14	13	17	7
Toluene A%	8	23	24	14
Bromo Benzene A%	6	19	19	32
Benzyl Mesylate A%	0	0	0	0

5. Stirring Time for Benzyl Mesylate Formation

Chart S9. Selectivity Data for the *in situ* Formation of Benzyl Mesylate.

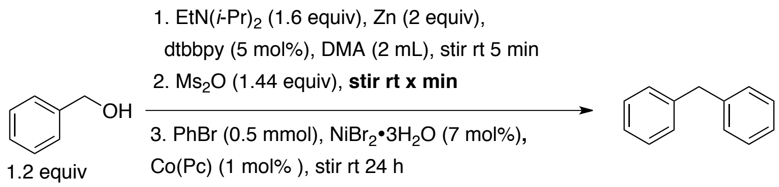

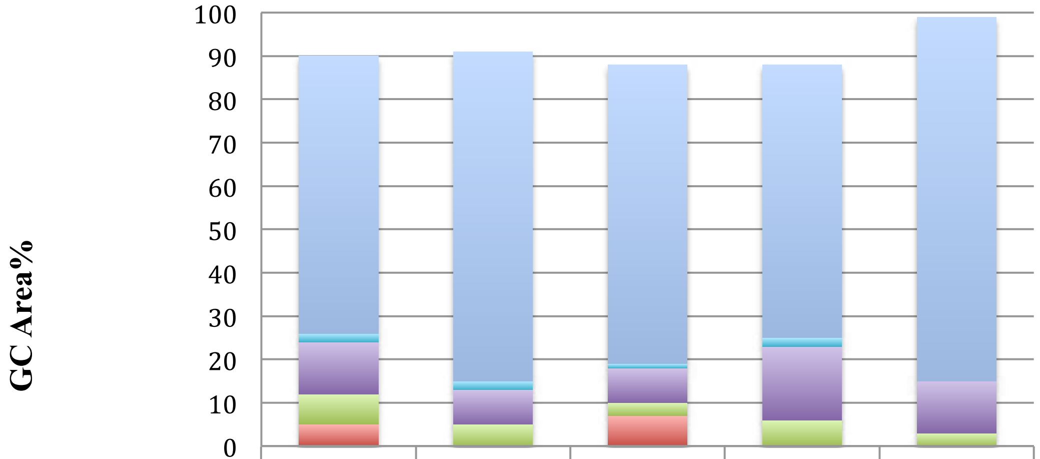

	5 min	10 min	20 min	30 min	60 min
Product A%	64	76	69	63	84
Biphenyl A%	0	0	0	0	0
Bibenzyl A%	2	2	1	2	0
Benzene A%	12	8	8	17	12
Toluene A%	7	5	3	6	3
Bromo Benzene A%	5	0	7	0	0
Benzyl Mesylate A%	0	0	0	0	0

6. Solvent

Chart S10. Selectivity Data for Selected Solvents.

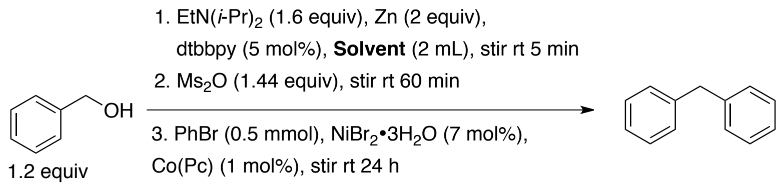

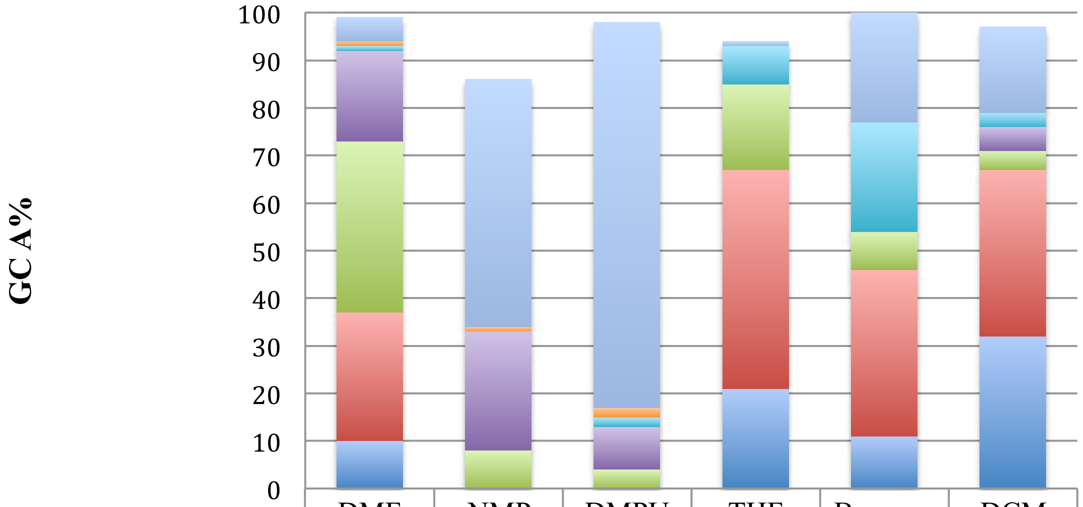

	DMF	NMP	DMPU	THF	Benzene	DCM
Product A%	5	52	81	1	23	18
Biphenyl A%	1	1	2	0	0	0
Bibenzyl A%	1	0	2	8	23	3
Benzene A%	19	25	9	0	0	5
Toluene A%	36	8	4	18	8	4
Bromo Benzene A%	27	0	0	46	35	35
Benzyl Mesylate A%	10	0	0	21	11	32

Solvent Key:
DMF-*N*,*N*-dimethylacetamide
NMP-*N*-methyl-2-pyrrolidone
DMPU-1,3-dimethyl-3,4,5,6-tetrahydro-2(1H)-pyrimidinone
THF-tetrahydrofuran
DCM-dichloromethane

IV. Characterization Data

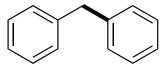

Diphenylmethane (3a) (Scheme 2, X=Br) [101-81-5][6]

The general procedure A was followed with bromobenzene (79 mg, 0.50 mmol) and benzyl alcohol (62 μL, 0.60 mmol). Purification by flash chromatography (100% hexanes) afforded 67 mg (80% yield) of the title compound as a colorless oil. Analytical data matched those reported in the literature: ^{1}H NMR (500 MHz; $CDCl_3$) δ 7.30-7.18 (m, 10H), 3.99 (s, 2H). GC-MS (EI+) *m/z* (% rel. int., ion): 168.10 (100.00, M^+).

Diphenylmethane (3a) (Scheme 2, X=I) [101-81-5][6]

The general procedure A was followed with iodobenzene (102 mg, 0.500 mmol) and benzyl alcohol (62 μL, 0.60 mmol). Purification by flash chromatography (100% hexanes) afforded 61 mg (73% yield) of the title compound as a colorless oil. Analytical data matched those reported in the literature: ^{1}H NMR (500 MHz; $CDCl_3$) δ 7.40-7.28 (m, 10H), 4.08 (s, 2H). ^{13}C NMR (126 MHz; $CDCl_3$) δ 141.2, 129.1, 128.6, 126.2, 42.0 GC-MS (EI+) *m/z* (% rel. int., ion): 168.10 (100.00, M^+). HRMS (EI+, *m/z*): [M^+] calcd for $C_{13}H_{12}$, 168.0939; found, 168.094.

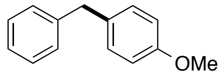

1-benzyl-4-methoxybenzene (3b) (Scheme 2, X=Br) [834-14-0][6]

The general procedure A was followed with 1-bromo-4-methoxybenzene (94 mg, 0.50 mmol) and benzyl alcohol (62 μL, 0.60 mmol). Purification by flash chromatography (5% ethyl acetate in hexanes) afforded 50 mg (50% yield) of the title compound as a colorless oil. Analytical data matched those reported in the literature: ^{1}H-NMR (500 MHz; $CDCl_3$): δ 7.29-7.09 (m, 7H), 6.83-6.82 (d, *J* = 8.5 Hz, 2H), 3.92 (s, 2H), 3.78 (s, 3H). GC-MS (EI+) *m/z* (% rel. int., ion): 198.05(100.00, M^+).

1-benzyl-4-methoxybenzene (3b) (Scheme 2, X=I) [834-14-0][6]

The general procedure A was followed with 1-iodo-4-methoxybenzene (117 mg, 0.500 mmol) and benzyl alcohol (62 μL, 0.60 mmol). Purification by flash chromatography (5% ethyl acetate in hexanes) afforded 69 mg (70% yield) of the title compound as a colorless oil. Analytical data matched those reported in the literature: ^{1}H-NMR (500 MHz; $CDCl_3$): δ 7.33-7.13 (m, 7H), 6.88-6.86 (d, *J* = 8.5 Hz, 2H), 3.96 (s, 2H), 3.82 (s, 3H). ^{13}C NMR (126 MHz; $CDCl_3$): δ 157.9, 141.5, 133.1, 129.7, 128.7, 128.3, 125.9, 113.8, 55.1, 40.9. GC-MS (EI+) *m/z* (% rel. int., ion): 198.05 (100.00, M^+). HRMS (EI+, *m/z*): [M^+] calcd for $C_{14}H_{14}O$, 198.1045; found, 198.105.

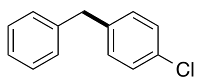

1-benzyl-4-chlorobenzene (3c) (Scheme 2, X=Br) [831-81-1][7]

The general procedure A was followed with 1-bromo-4-chlorobenzene (96 mg, 0.50 mmol) and benzyl alcohol (62 μL, 0.60 mmol). Purification by flash chromatography (100% hexanes) afforded 74 mg (73% yield) of the title compound as a colorless oil. Analytical data matched those reported in the literature: ^{1}H NMR (500 MHz; $CDCl_3$): δ 7.31-7.10 (m, 9H), 3.95 (s, 2H). GC-MS (EI+) *m/z* (% rel. int., ion): 202.05 (37.26, M^+), 167.10 (100.00, M^+-Cl).

1-benzyl-4-chlorobenzene (3c) (Scheme 2, X=I) [831-81-1][7]

The general procedure A was followed with 1-iodo-4-chlorobenzene (119 mg, 0.500 mmol) and benzyl alcohol (62 μL, 0.60 mmol). Purification by flash chromatography (100% hexanes) afforded 67 mg (66% yield) of the title compound as a colorless oil. Analytical data matched those reported in the literature: ^{1}H NMR (500 MHz; $CDCl_3$): δ 7.32-7.14 (m, 9H), 3.98 (s, 2H). ^{13}C NMR (126 MHz; $CDCl_3$) δ 141.1, 140.1, 132.4, 130.8, 129.4, 129.1, 126.8, 41.8. GC-MS (EI+) *m/z* (% rel. int., ion): 202.05 (100.00, M^+). HRMS (EI+, *m/z*): [M^+] calcd for $C_{13}H_{11}Cl$, 202.0549; found, 202.055.

---

[6] Maity, P.; Shacklady-McAtee, D. M.; Yap, G. P.; Sirianni, E. R.; Watson, M. P. *J. Am. Chem. Soc.* **2013**, *135*, 280-285.

[7] Cheng, Y; Dong, W; Wang, L. *Org. Lett.* **2014**, *16*, 2000-2002.

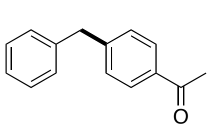

1-(4-benzylphenyl)ethan-1-one (3d) (Scheme 2, X=I) [782-92-3][8]

The general procedure A was followed with 1-(4-iodophenyl)ethan-1-one (123 mg, 0.500 mmol) and benzyl alcohol (62 µL, 0.60 mmol). Purification by flash chromatography (8% ethyl acetate in hexanes) afforded 75 mg (71% yield) of the title compound as a white solid. Analytical data matched those reported in the literature. mp 38-39 °C (lit.[9] 39 °C). ^{1}H NMR (500 MHz; $CDCl_3$) δ 7.92-7.91 (d, J = 8.2 Hz, 2H), 7.33-7.20 (m, 7H), 4.07 (s, 2H), 2.61 (s, 3H). ^{13}C NMR (126 MHz; $CDCl_3$) δ 198.3, 147.3, 140.5, 135.8, 129.6, 129.4, 129.1, 126.9, 42.4, 27.1. IR (ATR, cm^{-1}): 1674 (C=O, strong). GC-MS (EI+) *m/z* (% rel. int., ion): 210.10 (44.47, M^+), 195.05 (100.00, M^+-CH_3). HRMS (EI+, *m/z*): [M^+] calcd for $C_{15}H_{14}O$, 210.1045; found, 210.105.

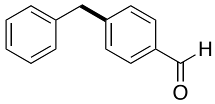

4-benzylbenzaldehyde (3e) (Scheme 2, X=Br) [67468-65-9][10]

The general procedure A was followed with 4-bromobenzaldehyde (92 mg, 0.50 mmol) and benzyl alcohol (62 µL, 0.60 mmol). Purification by flash chromatography (9% ethyl acetate in hexanes) afforded 86 mg (88 % yield) of the title compound as a colorless oil. Analytical data matched those reported in the literature: ^{1}H NMR (500 MHz; $CDCl_3$) δ 9.98 (s, 1H), 7.82-7.80 (d, J = 8.1 Hz, 2H), 7.36-7.17 (m, 7H), 4.06 (s, 2H). GC-MS (EI+) *m/z* (% rel. int., ion): 196.09 (100.00, M^+).

4-benzylbenzaldehyde (3e) (Scheme 2, X=I) [67468-65-9][10]

The general procedure A was followed with 4-iodobenzaldehyde (116 mg, 0.500 mmol) and benzyl alcohol (62 µL, 0.60 mmol). Purification by flash chromatography (9% ethyl acetate in hexanes) afforded 67 mg (68 % yield) of the title compound as a colorless oil. Analytical data matched those reported in the literature: ^{1}H NMR (500 MHz; $CDCl_3$) δ 10.00 (s, 1H), 7.84-7.83 (d, J = 8.1 Hz, 2H), 7.39-7.21 (m, 7H), 4.09 (s, 2H). ^{13}C NMR (126 MHz; $CDCl_3$) δ 192.1, 148.5, 139.9, 134.8, 130.2, 129.7, 129.1, 128.8, 126.7, 42.2. GC-MS (EI+) *m/z* (% rel. int., ion): 196.05 (100.00, M^+). HRMS (EI+, *m/z*): [M^+] calcd for $C_{14}H_{12}O$, 196.0888; found, 196.089.

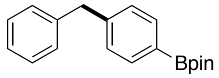

2-(4-benzylphenyl)-4,4,5,5-tetramethyl-1,3,2-dioxaborolane (3f) (Scheme 2, X=I)

The general procedure A was followed with 2-(4-iodophenyl)-4,4,5,5-tetramethyl-1,3,2-dioxaborolane (165 mg, 0.500 mmol) and benzyl alcohol (62 µL, 0.60 mmol). Purification by flash chromatography (3% ethyl acetate in hexanes) afforded 128 mg (87% yield) of the title compound as a white solid. mp 80-82 °C. ^{1}H NMR (500 MHz; $CDCl_3$) δ 7.78-7.76 (d, J = 7.9 Hz, 2H), 7.32-7.19 (m, 7H), 4.03 (s, 2H), 1.36 (s, 12H). ^{13}C NMR (126 MHz; $CDCl_3$) δ 144.5, 141.0, 135.2, 129.1, 128.6, 128.6, 126.2, 83.8, 42.3, 25.0. IR (ATR, cm^{-1}): 1354, 1323 (C-O, strong). GC-MS (EI+) *m/z* (% rel. int., ion): 294.20 (100.00, M^+). HRMS (EI+, *m/z*): [M^+] calcd for $C_{19}H_{23}O_2B$, 294.1791; found, 294.179.

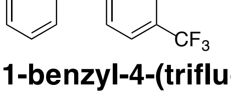

1-benzyl-4-(trifluoromethyl)benzene (3g) (Scheme 2, X=Br) [34239-04-8][6]

The general procedure A was followed with 1-iodo-4-(trifluoromethyl)benzene (136 mg, 0.500 mmol) and benzyl alcohol (62 µL, 0.60 mmol). Purification by flash chromatography (3% ethyl acetate in hexanes) afforded 83 mg (70% yield) of the title compound as a colorless oil. Analytical data matched those reported in the literature: ^{1}H

[8] Molander, G. A.; Takatoshi, I. *Org. Lett.* **2001**, *3*, 393-396.
[9] Montaudo, G.; Caccamese, S.; Finocchiaro, P., *J. Am. Chem. Soc.* **1971**, *93*, 4202-4207.
[10] Elia, M. D.; Molander, G. A. *J. Org. Lett.* **2006**, *71,* 9198-9202.

NMR (500 MHz; $CDCl_3$) 7.54-7.52 (m, 2H), 7.32-7.17 (m, 7H), 4.03 (s, 2H). ^{19}F NMR (400 MHz; $CDCl_3$): δ -62.80. GC-MS (EI+) *m/z* (% rel. int., ion): 236.15 (64.45, M^+), 167.10 (100.00, M^+-CF_3).

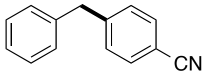

4-benzylbenzonitrile 4-benzylbenzonitrile (3h) (Scheme 2, X=I) [23450-31-9][11]

The general procedure A was followed with 4-iodobenzonitrile (115 mg, 0.500 mmol) and benzyl alcohol (62 μL, 0.60 mmol). Purification by flash chromatography (7% ethyl acetate in hexanes) afforded 71 mg (74% yield) of the title compound as a white solid. Analytical data matched those reported in the literature. mp 47-48 °C (lit.[12] 49-50 °C). ^{1}H NMR (500 MHz; $CDCl_3$) δ 7.60-7.59 (d, *J* = 8.1 Hz, 2H), 7.35-7.19 (m, 7H), 4.06 (s, 2H). ^{13}C NMR (126 MHz; $CDCl_3$) δ 146.8, 139.4, 132.4, 129.8, 129.1, 128.9, 126.8, 119.1, 42.1. IR (ATR, cm^{-1}): 2269 (C≡N, strong). GC-MS (EI+) *m/z* (% rel. int., ion): 193.10 (100.00, M^+). HRMS (EI+, *m/z*): [M^+] calcd for $C_{14}H_{11}N$, 193.0892; found, 193.089.

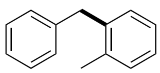

1-benzyl-2-methylbenzene (3i) (Scheme 2, X=Br) [713-36-0][13]

The general procedure A was followed with 1-bromo-2-methylbenzene (86 mg, 0.50 mmol) and benzyl alcohol (62 μL, 0.60 mmol). Purification by flash chromatography (100% hexanes) afforded 58 mg (64% yield) of the title compound as a colorless oil. Analytical data matched those reported in the literature: ^{1}H NMR (500 MHz; $CDCl_3$) δ 7.28-7.09 (m, 9H), 3.99 (s, 2H), 2.27 (s, 3H). GC-MS (EI+) *m/z* (% rel. int., ion): 182.15 (100.00, M^+).

1-benzyl-2-methylbenzene (3i) (Scheme 2, X=I) [713-36-0][13]

The general procedure A was followed with 1-iodo-2-methylbenzene (109 mg, 0.500 mmol) and benzyl alcohol (62 μL, 0.60 mmol). Purification by flash chromatography (100% hexanes) afforded 67 mg (74% yield) of the title compound as a colorless oil. Analytical data matched those reported in the literature: ^{1}H NMR (500 MHz; $CDCl_3$) δ 7.34-7.15 (m, 9H), 4.04 (s, 2H), 2.30 (s, 3H). ^{13}C NMR (126 MHz; $CDCl_3$) δ 140.7, 139.3, 137.0, 130.6, 130.3, 129.1, 128.7, 126.8, 126.3, 126.3, 39.8, 20.0. GC-MS (EI+) *m/z* (% rel. int., ion): 182.15 (65.34, M^+), 167.10 (100.00, M^+-CH_3). HRMS (EI+, *m/z*): [M^+] calcd for $C_{14}H_{14}$, 182.1096; found, 182.109.

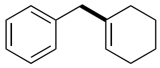

cyclohex-1-en-1-ylmethyl)benzene (3j) (Scheme 2, X=Br) [4714-09-4][14]

The general procedure A was followed with 1-iodocyclohex-1-ene (104 mg, 0.500 mmol) and benzyl alcohol (62 μL, 0.60 mmol). Purification by flash chromatography (100% hexanes) afforded 38 mg (44% yield) of the title compound as a colorless oil. Analytical data matched those reported in the literature: ^{1}H NMR (500 MHz; $CDCl_3$) δ 7.29-7.16 (m, 5H), 5.46 (s (broad), 1H), 3.24 (s, 2H), 2.01 (s (broad) 2H), 1.86 (s (broad) 2H), 1.61-1.54 (m, 4H). ^{13}C NMR (126 MHz; $CDCl_3$) δ 140.5, 137.2, 128.9, 128.2, 125.8, 123.0, 44.7, 28.1, 25.4, 23.0, 22.5. GC-MS (EI+) *m/z* (% rel. int., ion): 172.10 (46.56, M^+), 81.05 (100.00, M^+-C_7H_7).

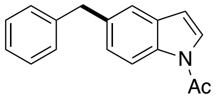

1-(5-benzyl-1*H*-indol-1-yl)ethan-1-one (3k) (Scheme 2, X=Br)

The general procedure A was followed with 1-(5-bromo-1*H*-indol-1-yl)ethan-1-one (104 mg, 0.500 mmol) and benzyl alcohol (62 μL, 0.60 mmol). Purification by flash chromatography (10% ethyl acetate in hexanes) afforded 65 mg (52% yield) of the title compound as a white solid. mp 66-67 °C. IR (ATR, cm^{-1}): 1697 (C=O, strong), 1323

---

11 Bernhardt, S.; Knochel, P.; Shen, Z. *Chem. Eur. J.* **2013**, *19*, 828-833.

12 Young, J. R.; Stevenson, G. R.; Bauld, N. L. *J. Am. Chem. Soc.* **1972**, *94*, 8790-8794.

13 McLaughlin, M. Org. Lett. **2005**, *7*, 4875-4878.

14 Piber, M; Jensen, A. E.; Rottlaender, M.; Knochel, P. Org. Lett. **1999**, *1,* 1323-1326.

(C-N, strong).^{1}H-NMR (500 MHz; $CDCl_3$): δ 8.38-8.36 (m, 1H), 7.42-7.39 (m, 2H), 7.33-7.29 (m, 2H), 7.25-7.22 (m, 4H), 6.60 (d, J = 3.8 Hz, 1H), 4.11 (s, 2H), 2.65 (s, 3H). ^{13}C NMR (126 MHz; $CDCl_3$) δ168.6, 141.6, 136.7, 134.3, 130.9, 129.0, 128.6, 126.5, 126.2, 125.5, 130.0, 116.5, 109.2, 42.0, 24.0. GC-MS (EI+) *m/z* (% rel. int., ion): 249.05 (76.38, M^+), 207.05 (100.00, M^+-C_2H_3O). HRMS (EI+, *m/z*): [M^+] calcd for $C_{17}H_{15}NO$, 249.1154; found, 249.116.

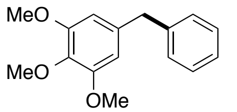

5-benzyl-1,2,3-trimethoxybenzene (3l) (Scheme 2, X=I)

The general procedure A was followed with iodobenzene (56 μL, 0.50 mmol) and 3,4,5-trimethoxyphenyl)methanol (119 mg, 0.600 mmol). Purification by flash chromatography (10% ethyl acetate in hexanes) afforded 94 mg (73% yield) of the title compound as a white solid. Mp 37-38 °C. ^{1}H NMR (500 MHz; $CDCl_3$) δ 7.31-7.19 (m, 5H), 6.40 (s, 2H), 3.92 (s, 2H), 3.82 (s, 3H), 3.81 (s, 6H). ^{13}C NMR (126 MHz; $CDCl_3$) δ 152.8, 140.5, 136.3, 136.0, 128.4, 128.1, 125.8, 105.6, 60.5, 55.7, 41.9. IR (ATR, cm^{-1}): 1123 (C-O, strong). GC-MS (EI+) *m/z* (% rel. int., ion): 258.13 (100.00, M^+). HRMS (EI+, *m/z*): [M^+] calcd for $C_{16}H_{18}O_3$, 258.1256; found, 258.126.

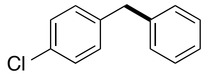

1-benzyl-4-chlorobenzene (3c') (Scheme 2, X=Br) [831-81-1][7]

The general procedure A was followed with bromobenzene (52 μL, 0.50 mmol) and 4-chlorophenyl)methanol (86 mg, 0.60 mmol). Purification by flash chromatography (100% hexanes) afforded 82 mg (81% yield) of the title compound as a colorless oil. Analytical data matched those reported in the literature: ^{1}H NMR (500 MHz; $CDCl_3$) δ 7.30-7.10 (m, 9H), 3.94 (s, 2H). GC-MS (EI+) *m/z* (% rel. int., ion): 202.05 (78.86, M^+), 167.05 (100.00, M^+-Cl).

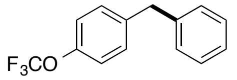

1-benzyl-4-(trifluoromethoxy)benzene (3m) (Scheme 2, X=Br)[15]

The general procedure A was followed with bromobenzene (52 μL, 0.50 mmol) and 4-trifluoromethoxy)phenyl)methanol (115 mg, 0.600 mmol). Purification by flash chromatography (9 % ethyl acetate in hexanes) afforded 98 mg (78% yield) of the title compound as a colorless oil. ^{1}H NMR (500 MHz; $CDCl_3$) δ 7.31-7.11 (m, 9H), 3.97 (s, 2H). ^{13}C NMR (126 MHz; $CDCl_3$) δ 148.0, 140.8, 140.3, 130.5, 129.3, 129.0, 126.8, 121.4(broad), 41.6. ^{19}F NMR (400 MHz; $CDCl_3$) δ -58.34. GC-MS (EI+) *m/z* (% rel. int., ion): 252.15 (100.00, M^+). HRMS (EI+, *m/z*): [M^+] calcd for $C_{14}H_{11}OF_3$, 252.0762; found, 252.076.

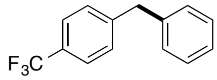

1-benzyl-4-(trifluoromethyl)benzene (3g') (Scheme 2, X=Br) [34239-04-8][6]

The general procedure A was followed with bromobenzene (52 μL, 0.50 mmol) and 4-(trifluoromethyl)phenyl)methanol (106 mg, 0.600 mmol). Purification by flash chromatography (3% ethyl acetate in hexanes) afforded 66 mg (56% yield) of the title compound as a colorless oil. Analytical data matched those reported in the literature: ^{1}H NMR (500 MHz; $CDCl_3$) δ 7.54-7.52 (d, J= 7.8 Hz, 2H), δ 7.31-7.16 (m, 7H), 4.03 (s, 2H). ^{13}C NMR (126 MHz; $CDCl_3$) δ 145.3, 140.1, 129.3, 129.1, 128.8, 126.6, 125.5, 125.5, 123.4, 41.9. ^{19}F NMR (400 MHz; $CDCl_3$) δ -62.78. GC-MS (EI+) *m/z* (% rel. int., ion): 236.05 (97.27, M^+), 167.15 (100.00, M^+-CF_3).

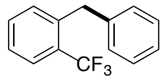

1-benzyl-2-(trifluoromethyl)benzene (3n) (Scheme 2, X=Br) [64675-45-2][16]

---

[15] Olah, G. A.; Yamato, T.; Hashimoto, T. *J. Am. Chem. Soc.* **1987**, 109, 3708-3713.

The general procedure A was followed with bromobenzene (52 μL, 0.50 mmol) and 2-(trifluoromethyl)phenyl)methanol (106 mg, 0.600 mmol). Purification by flash chromatography (3 % ethyl acetate in hexanes) afforded 63 mg (53% yield) of the title compound as a colorless oil. ^{1}H NMR (500 MHz; $CDCl_3$) δ 7.67-7.65 (m, 1H), δ 7.43-7.40 (m, 1H), δ 7.31-7.28 (m, 3H), δ 7.23-7.22 (m, 1H), δ 7.17-7.14 (m, 3H), 4.19 (s, 2H). ^{13}C NMR (126 MHz; $CDCl_3$) δ 139.8, 139.4, 131.6 (broad), 131.6, 129.0, 128.4, 126.2 (broad), 126.1, 125.7 (quartet), 125.6, 123.4, 37.7. ^{19}F NMR (400 MHz; $CDCl_3$) δ -60.07. GC-MS (EI+) *m/z* (% rel. int., ion): 235.95 (100.00, M^+).

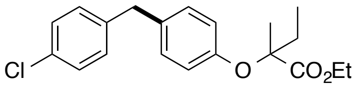

Ethyl 2-(4-(4-chlorobenzyl)phenoxy)-2-methylbutanoate (Beclobrate) (3o) (Scheme 2, X=Br) [55937-99-0][17]

The general procedure A was followed with ethyl 2-(4-bromophenoxy)-2-methylbutanoate (synthetic procedure on S4) (151 mg, 0.500 mmol) and 4-chlorophenyl)methanol (86 mg, 0.60 mmol). Purification by flash chromatography (10% ethyl acetate in hexanes) afforded 104 mg (60% yield) of the title compound as a colorless oil. Analytical data matched those reported in the literature: ^{1}H-NMR (500 MHz; $CDCl_3$): δ 7.27 (d, J = 8.4 Hz, 2H), 7.12 (d, J = 8.3 Hz, 2H), 7.04 (d, J = 8.5 Hz, 2H), 6.81 (d, J = 8.5 Hz, 2H), 4.26 (qd, J = 7.1, 1.1 Hz, 2H), 3.90 (s, 2H), 2.00 (td, J = 16.3, 8.8 Hz, 2H), 1.51 (s, 3H), 1.28 (t, J = 7.1 Hz, 3H), 1.01 (t, J = 7.5 Hz, 3H). ^{13}C NMR (126 MHz; $CDCl_3$): δ 174.2, 154.0, 140.0, 134.2, 131.9, 130.3, 129.6, 128.6, 119.5, 82.1, 61.4, 40.5, 32.7, 20.9, 14.3, 8.0. GC-MS (EI+) *m/z* (% rel. int., ion): 346.10 (75.95, M^+), 183.10 (100.00, M^+-$C_7H_{13}O_2$, -Cl).

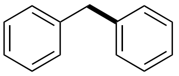

Diphenylmethane (3a') (Table 2, Entry 5) [101-81-5][6]

The general procedure B was followed with bromobenzene (79 mg, 0.50 mmol) and benzyl diethyl phosphate (146 mg, 0.600 mmol). Purification by flash chromatography (100% hexanes) afforded 56 mg (70% yield) of the title compound as a colorless oil. Analytical data matched those reported in the literature: ^{1}H-NMR (500 MHz; CDCl3): δ 7.4-7.3 (m, 4H), 7.3-7.2 (m, 6H), 4.0 (s, 2H). ^{13}C NMR (126 MHz; $CDCl_3$): δ 141.3, 129.1, 128.6, 26.2, 42.1. GC-MS (EI+) *m/z* (% rel. int., ion): 168.05 (100.00, M^+)

---

[16] Yen, S. K.; Koh, L. L.; Huynh, H. V.; Hor, T. S. A. *Eur. J. Inorg. Chem*. **2009**, *28,* 4288-4297.

[17] Mertins, K; Iovel, I; Kischel, J; Zapf, A; Beller, M. *Adv.Synth. Catal.* **2006**, 348, 691-695

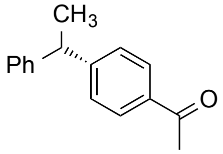

(S)-1-(4-(1-phenylethyl)phenyl)ethan-1-one (8) (Scheme 3) [1142813-40-8][18]

The general procedure B was followed with 1-(4-iodophenyl)ethan-1-one (123 mg, 0.500 mmol), (1-chloroethyl)benzene (70 mg, 0.50 mmol), and 2,2′-Bis[(4S)-4-benzyl-2-oxazoline] (16 mg, 0.05 mmol) in place of dtbbpy. Purification by flash chromatography (5% ethyl acetate in hexanes) afforded 46 mg (41% yield) of the title compound as a colorless oil. The isolated product was determined to have a 43% ee by SFC analysis (90/10 CO_2/*i*-PrOH, 4 mL/min flow rate, 12.0 MPa backpressure) on a Chiralpak IA column (25 cm long × 4.6 mm diameter): $t_{8\text{-}S}$= 3.0 min (71.5%) and $t_{8\text{-}R}$= 3.2 min (28.5%). The absolute configuration of **8** was confirmed by comparison of the optical rotation data from Imao et al.[18] $[\alpha]^{23}_{D}$ +3.57° (c 1.75, $CDCl_3$) is consistent with the (S) configuration for **8** and corresponds to 47% ee. Analytical data matched those reported in the literature: ^{1}H-NMR (500 MHz; CDCl3): δ 7.91 (d, J = 8.3 Hz, 2H), 7.33 (dd, J = 12.5, 7.9 Hz, 4H), 7.24-7.22 (m, 3H), 4.24 (q, J = 7.2 Hz, 1H), 2.60 (s, 3H), 1.69 (d, J = 7.2 Hz, 3H). ^{13}C NMR (126 MHz; CDCl3): δ 198.0, 152.2, 145.5, 135.4, 128.79, 128.74, 128.0, 127.8, 126.6, 45.0, 26.8, 21.8. GC-MS (EI+) *m/z* (% rel. int., ion): 224.05 (57.29, M^+), 209.05 (100.00, M^+-CH_3).

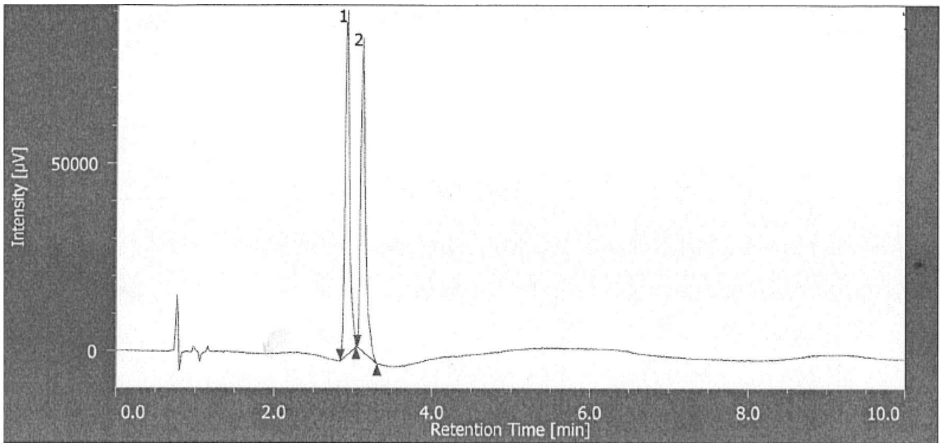

Image of SFC chromatogram for racemic **8** formed by reaction with dtbbpy as the ligand demonstrating separation of the racemic mixture on the IA column. λ= 235 nm

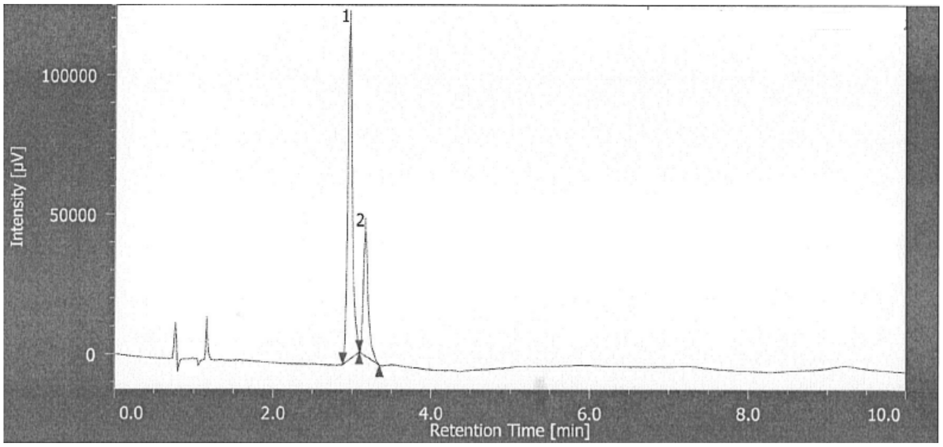

Image of SFC chromatogram for racemic **8** formed by reaction with 2,2′-Bis[(4S)-4-benzyl-2-oxazoline] as the ligand showing a 43% ee**.** λ= 235 nm

18 Imao, D; Glasspoole, B. W.; Laberge, V. S.; Crudden, C. M. *J. Am. Chem. Soc.* **2009**, 131, 5024-5025

V. Copies of NMR Spectra

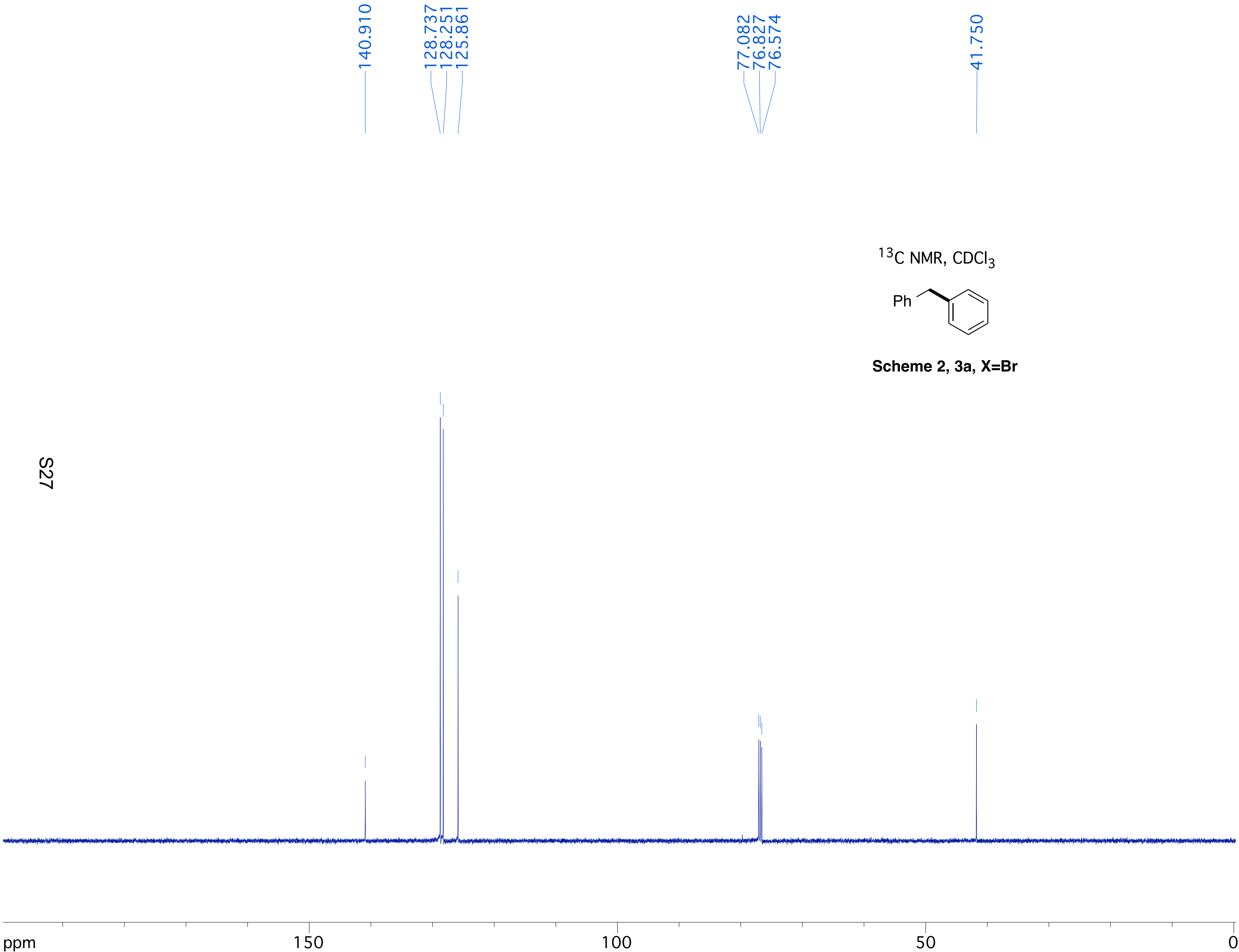
140.910
128.737
128.251
125.861
77.082
76.827
76.574
41.750
^{13}C NMR, $CDCl_3$
Ph
Scheme 2, 3a, X=Br
ppm
150
100
50
0

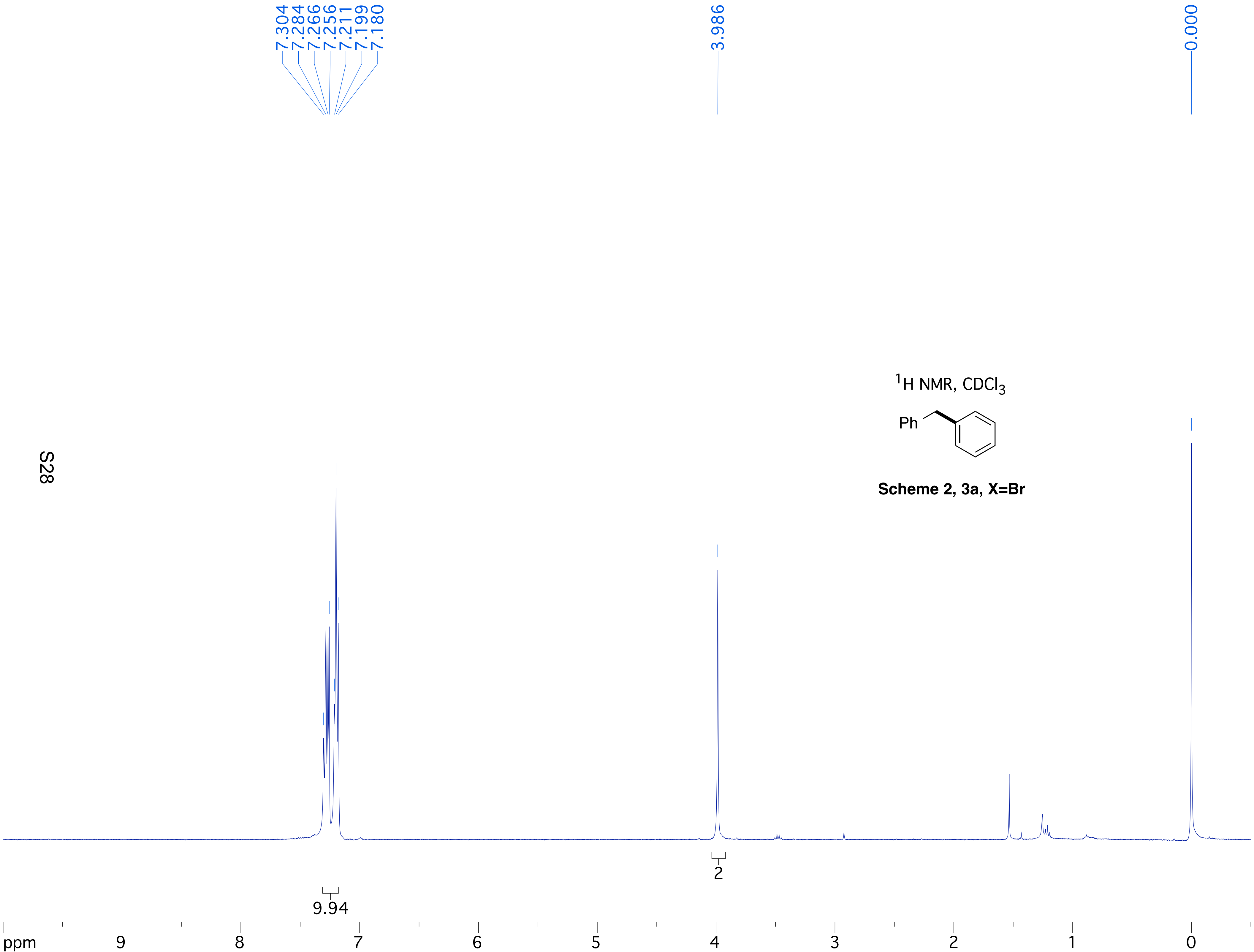

Scheme 2, 3a, X=Br

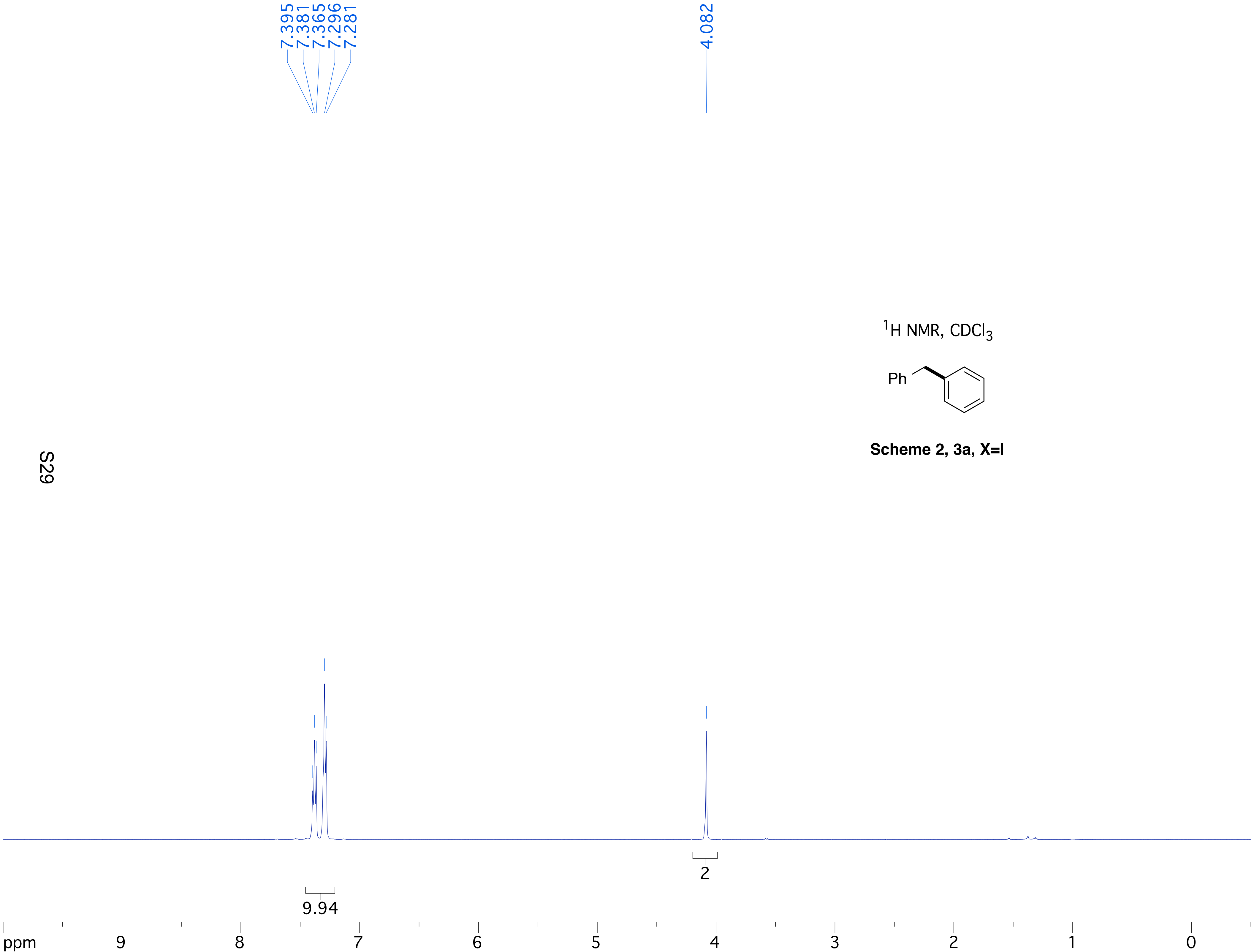
7.395
7.381
7.365
7.296
7.281
4.082
1H NMR, CDCl3
Ph
Scheme 2, 3a, X=I
9.94
2
ppm 9 8 7 6 5 4 3 2 1 0

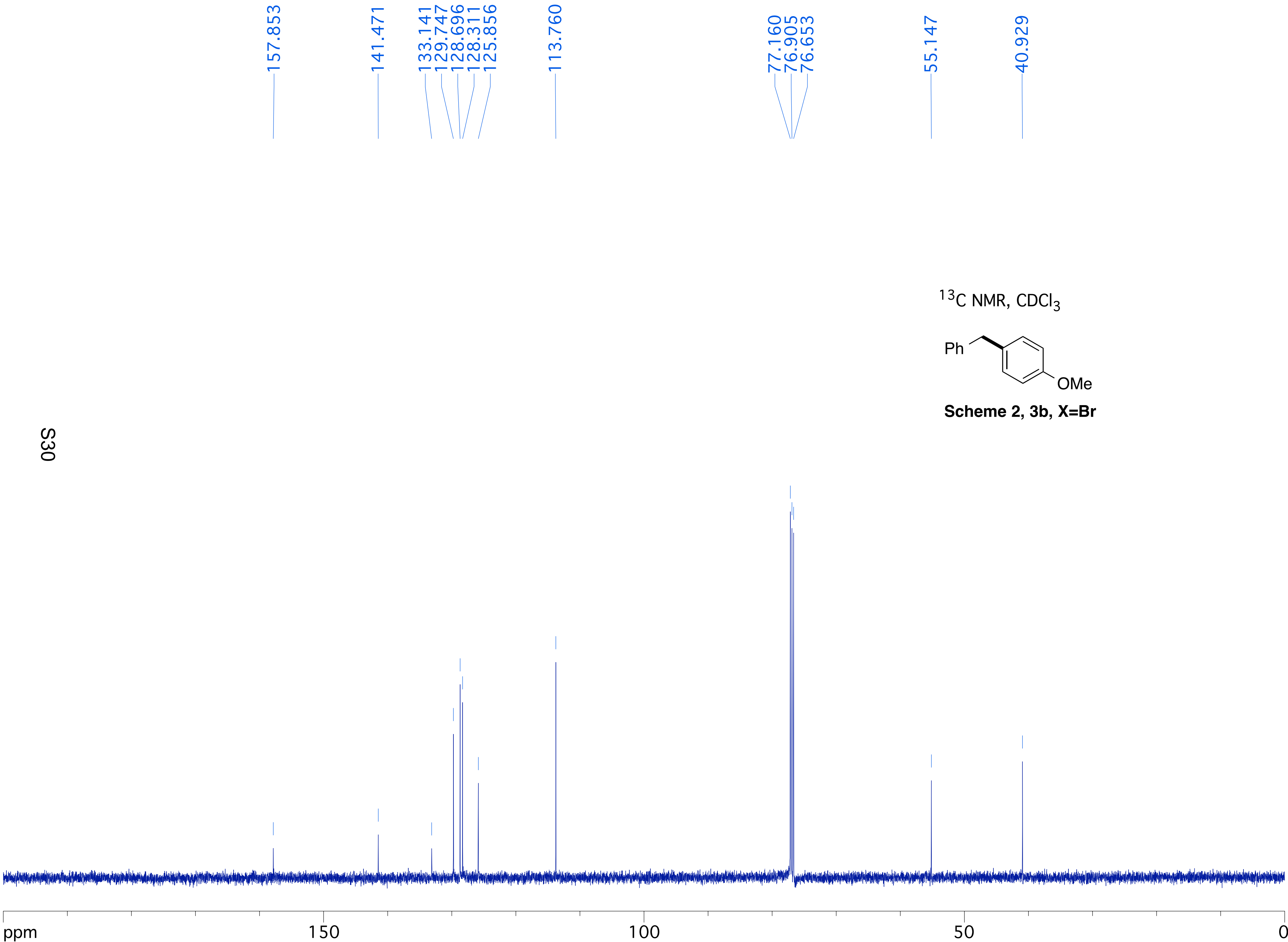

^{13}C NMR, $CDCl_3$

Scheme 2, 3b, X=Br

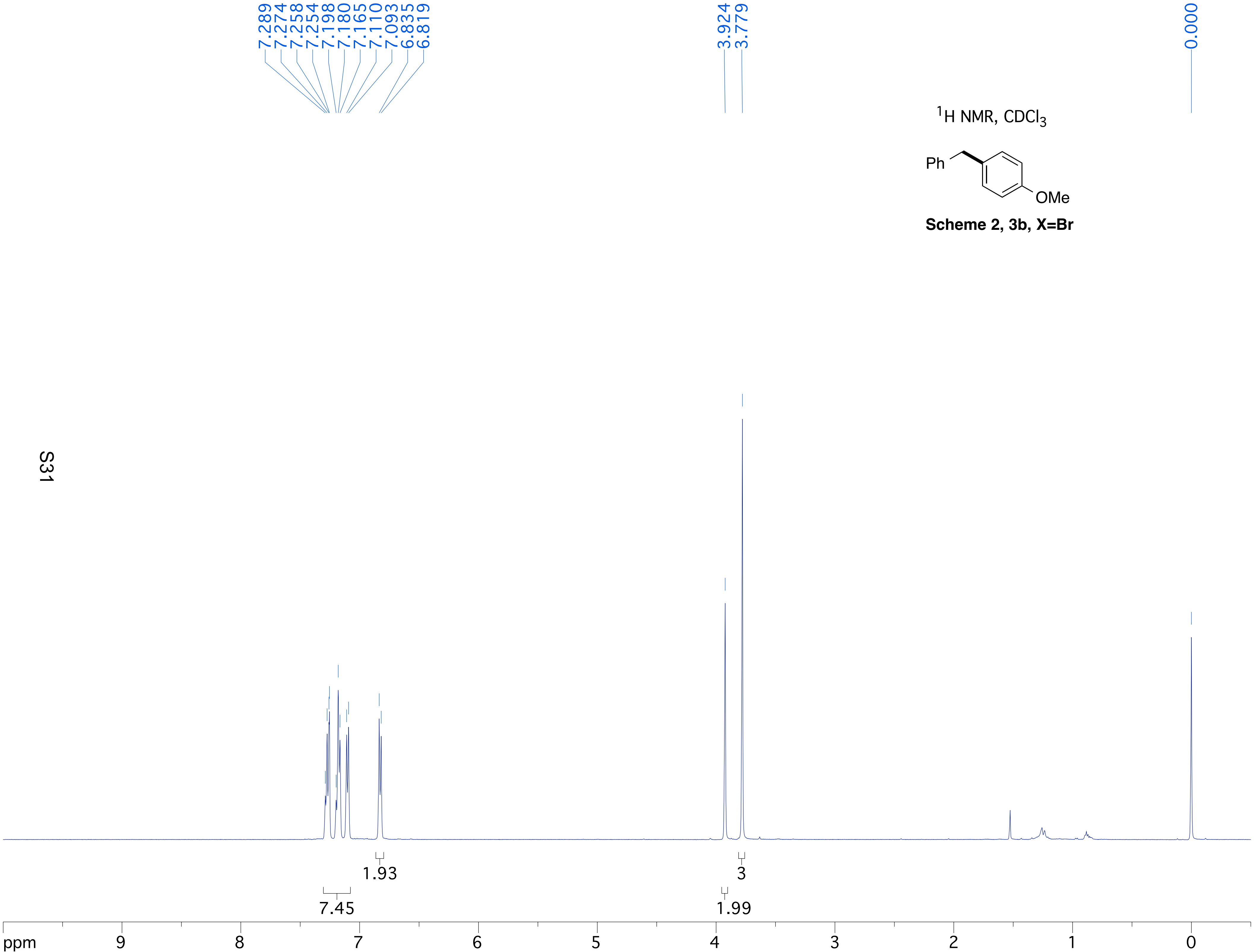

^1H NMR, $CDCl_3$

Scheme 2, 3b, X=Br

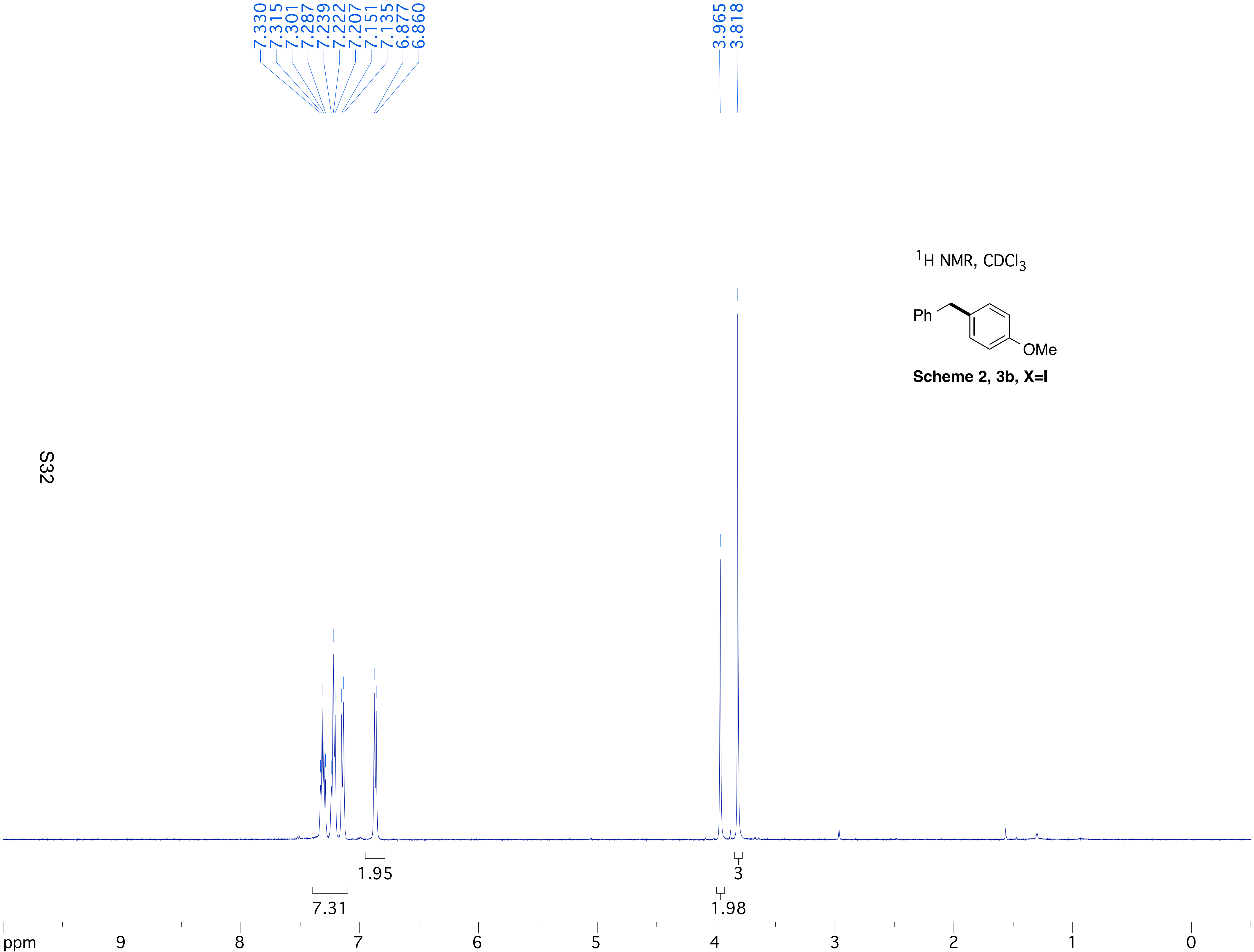
7.330
7.315
7.301
7.287
7.239
7.222
7.207
7.151
7.135
6.877
6.860
3.965
3.818
^{1}H NMR, $CDCl_3$
Ph
OMe
Scheme 2, 3b, X=I
1.95
7.31
3
1.98
ppm
9
8
7
6
5
4
3
2
1
0

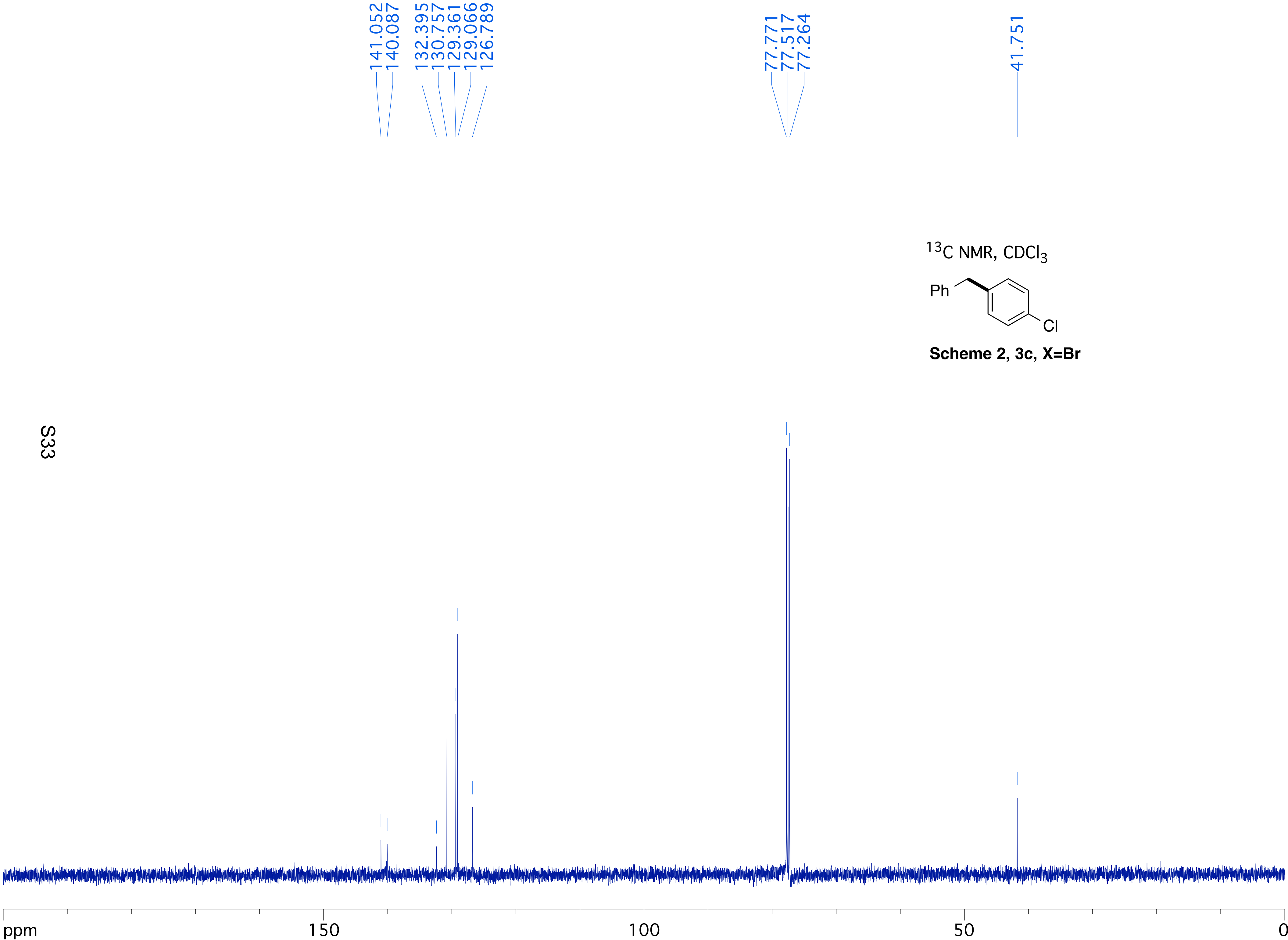

^{13}C NMR, $CDCl_3$

Scheme 2, 3c, X=Br

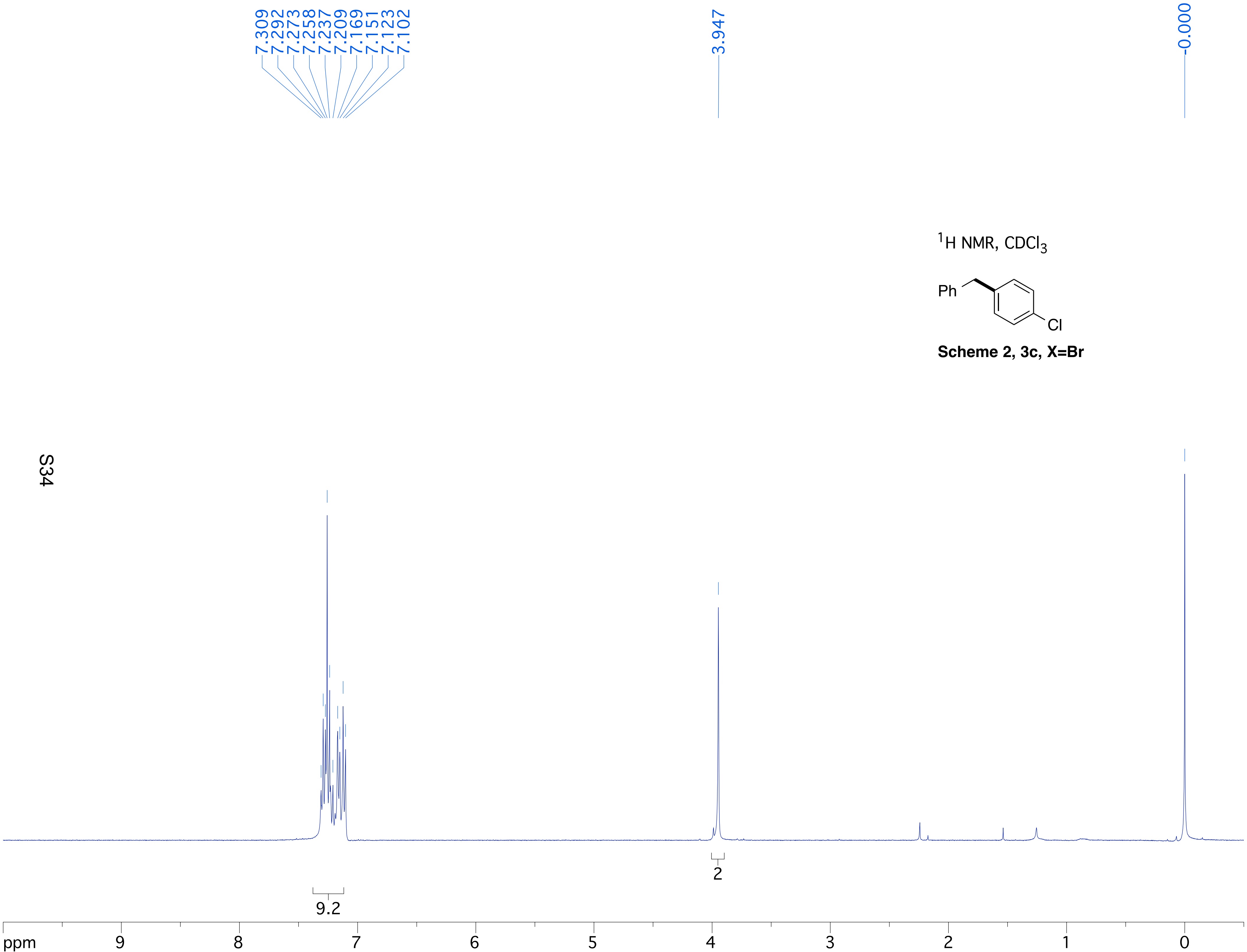

^1H NMR, $CDCl_3$

Scheme 2, 3c, X=Br

^{1}H NMR, $CDCl_3$

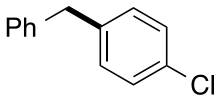

Scheme 2, 3c, X=I

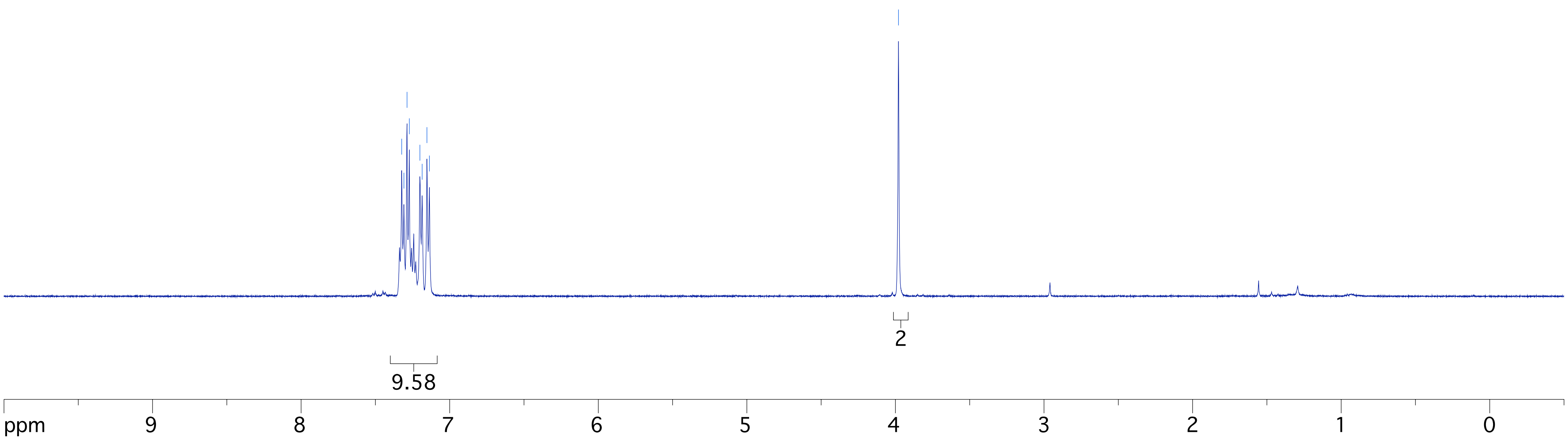

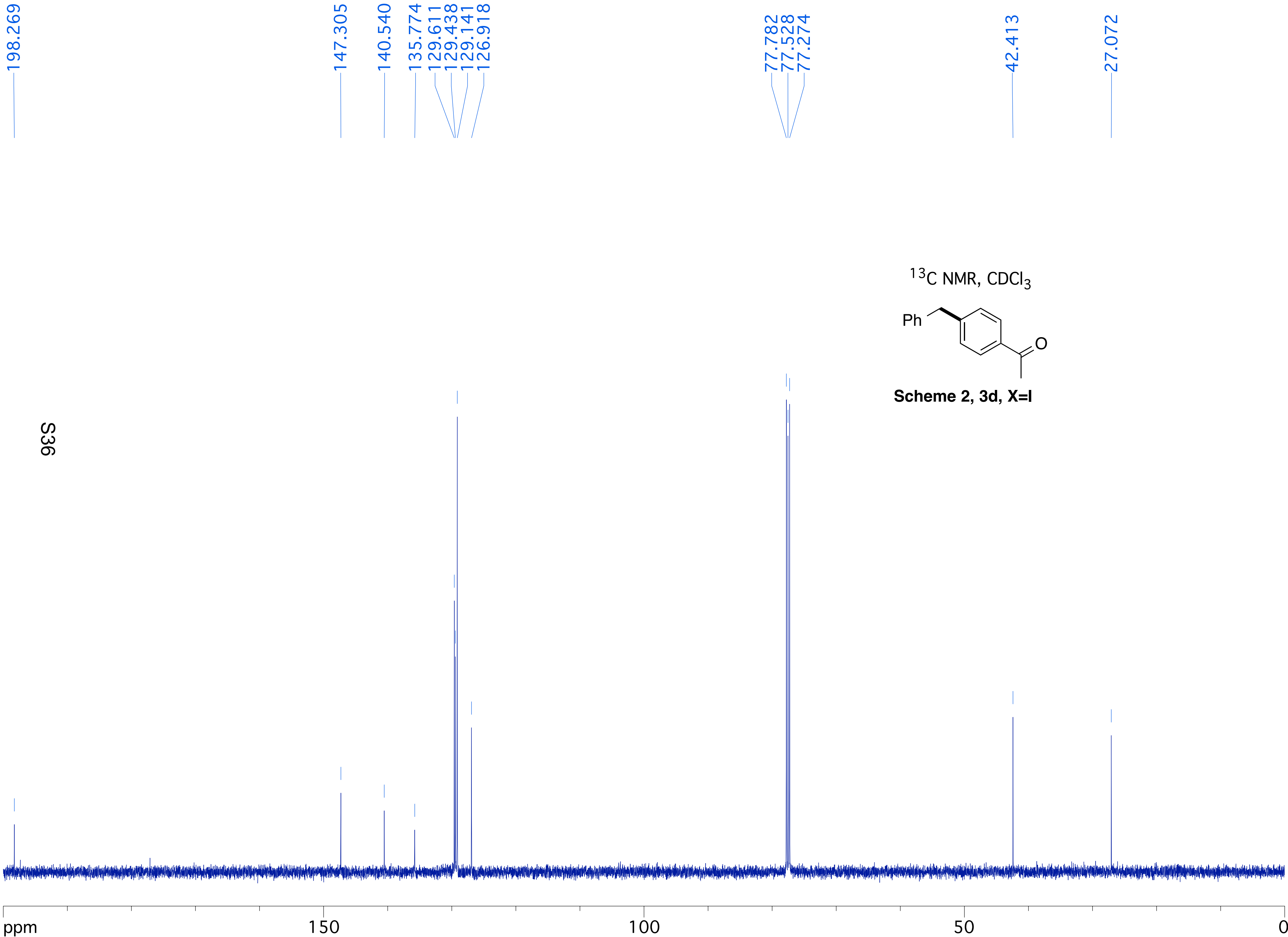

^{13}C NMR, $CDCl_3$

Scheme 2, 3d, X=I

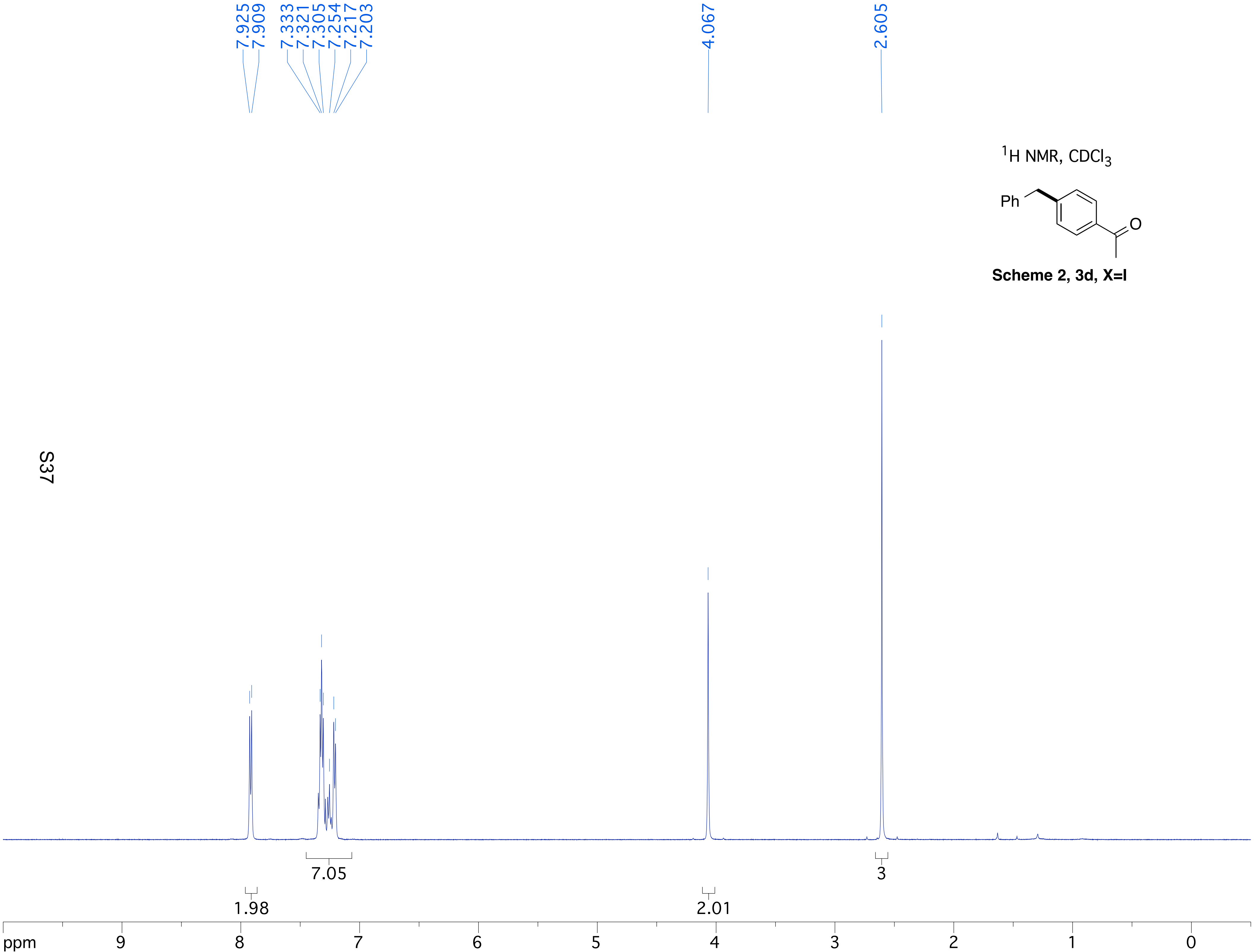

Scheme 2, 3d, X=I

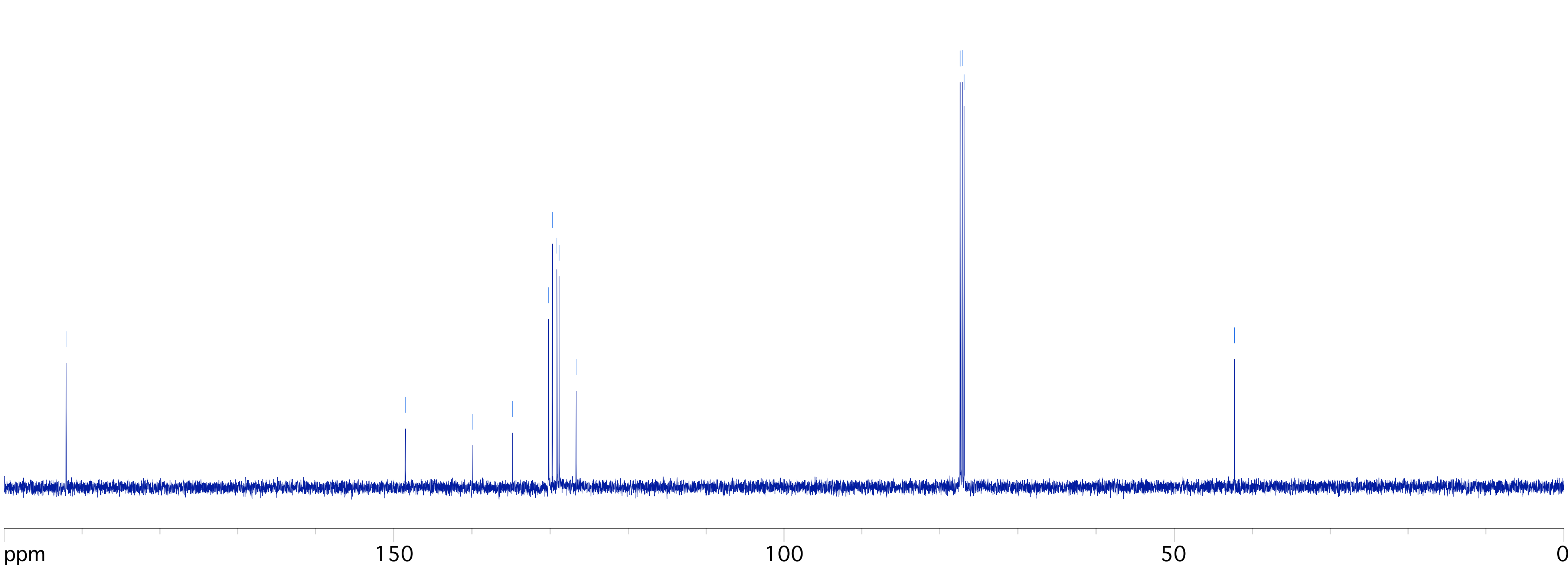

^{13}C NMR, $CDCl_3$

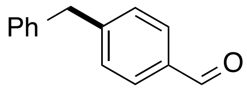

Scheme 2, 3e, X=Br

^1H NMR, $CDCl_3$

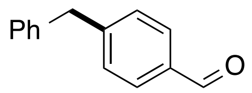

Scheme 2, 3e, X=Br

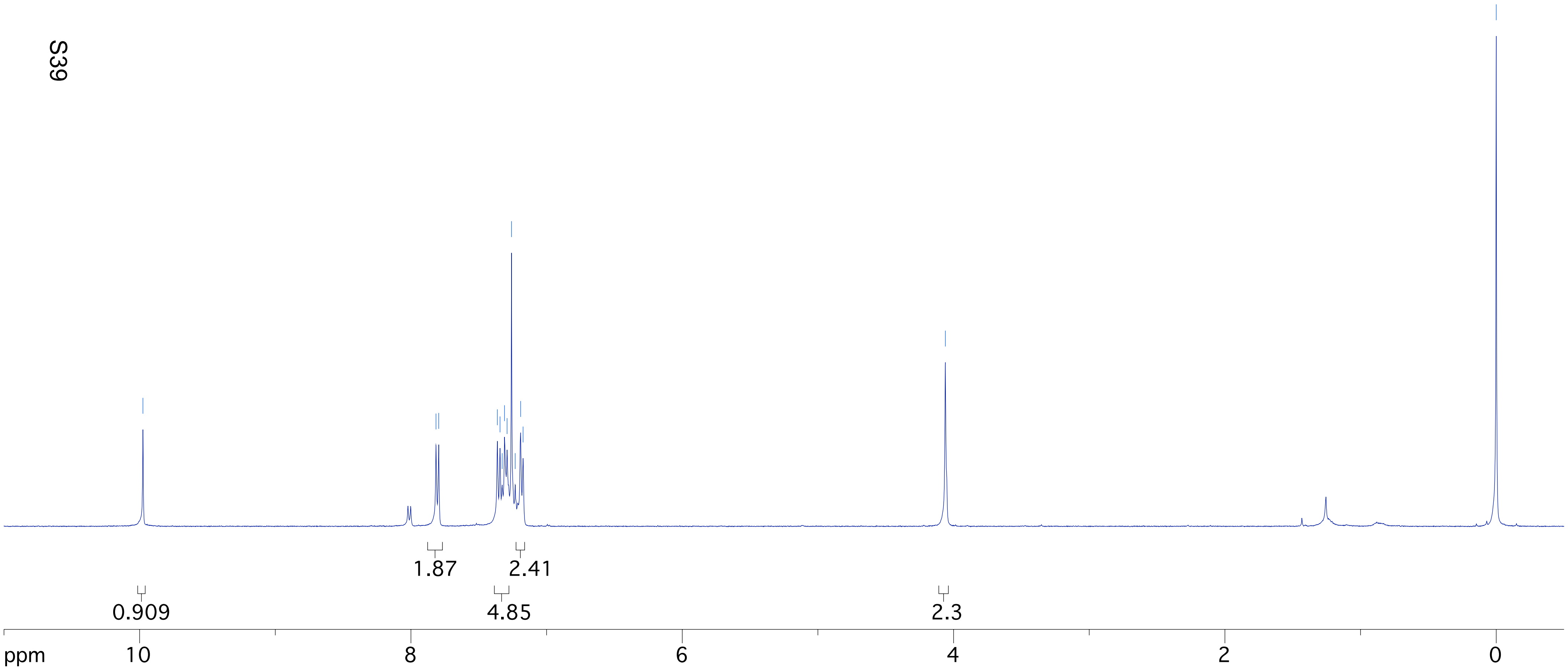

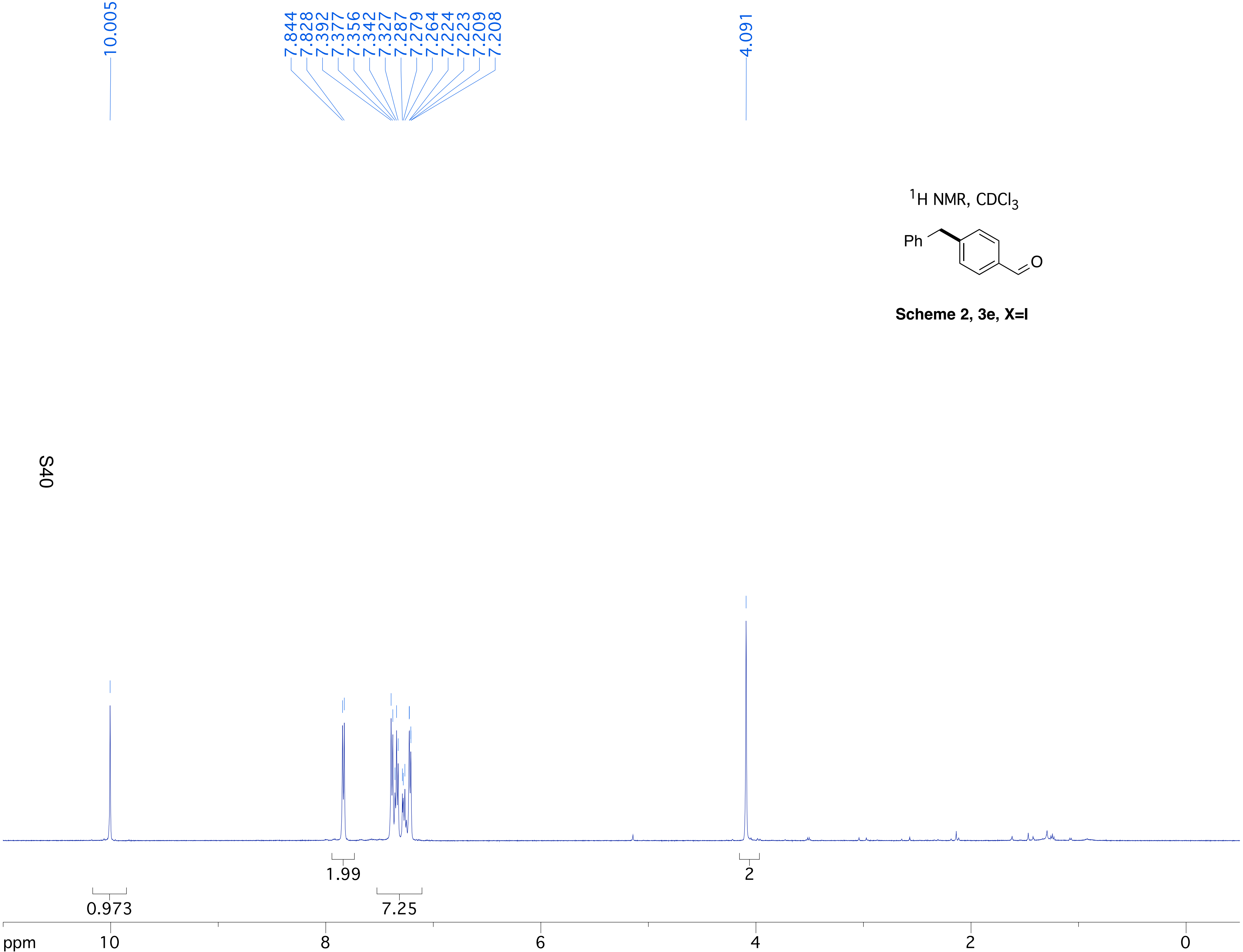

10.005
7.844
7.828
7.392
7.377
7.356
7.342
7.327
7.287
7.279
7.264
7.224
7.223
7.209
7.208
4.091
^{1}H NMR, $CDCl_3$
Ph
O
Scheme 2, 3e, X=I
0.973
1.99
7.25
2
ppm
10
8
6
4
2
0

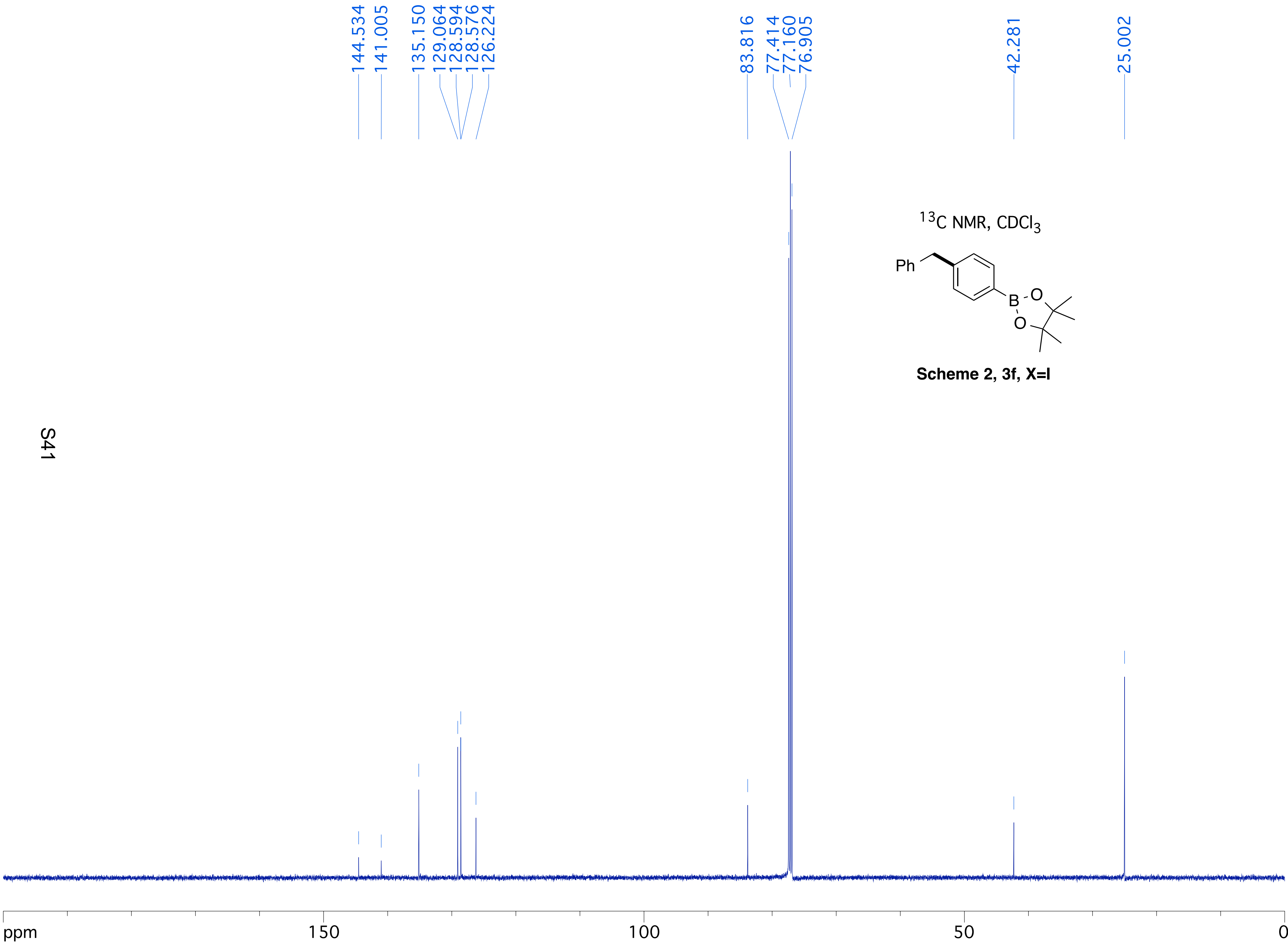

144.534
141.005
135.150
129.064
128.594
128.576
126.224
83.816
77.414
77.160
76.905
42.281
25.002
^{13}C NMR, $CDCl_3$
Ph
B
O
O
Scheme 2, 3f, X=I
ppm
150
100
50
0

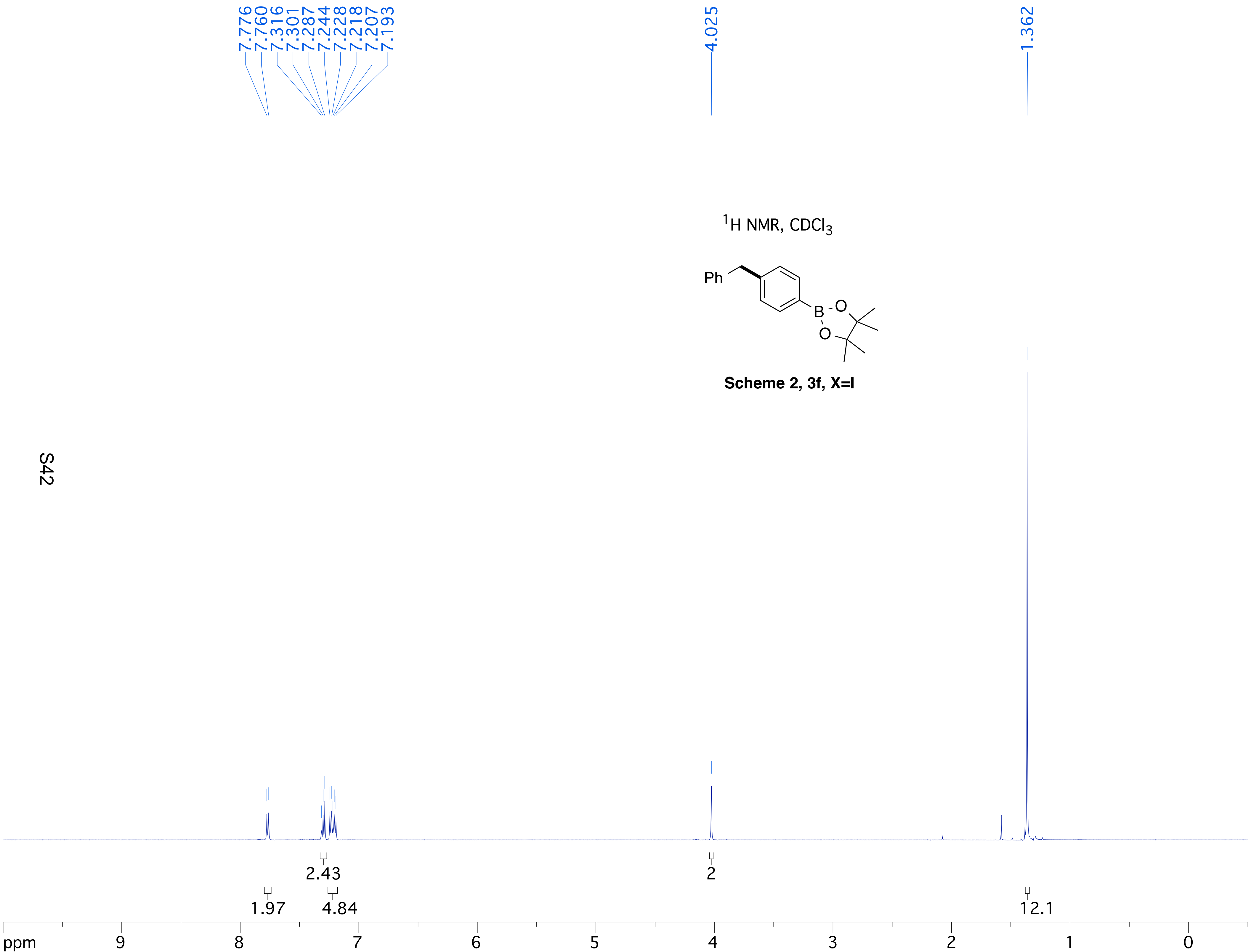
7.776
7.760
7.316
7.301
7.287
7.244
7.228
7.218
7.207
7.193
4.025
1.362
1H NMR, CDCl3
Ph
B
O
O
Scheme 2, 3f, X=I
2.43
2
1.97
4.84
12.1
ppm
9
8
7
6
5
4
3
2
1
0

^{19}F NMR, $CDCl_3$

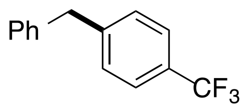

Scheme 2, 3g, X=Br

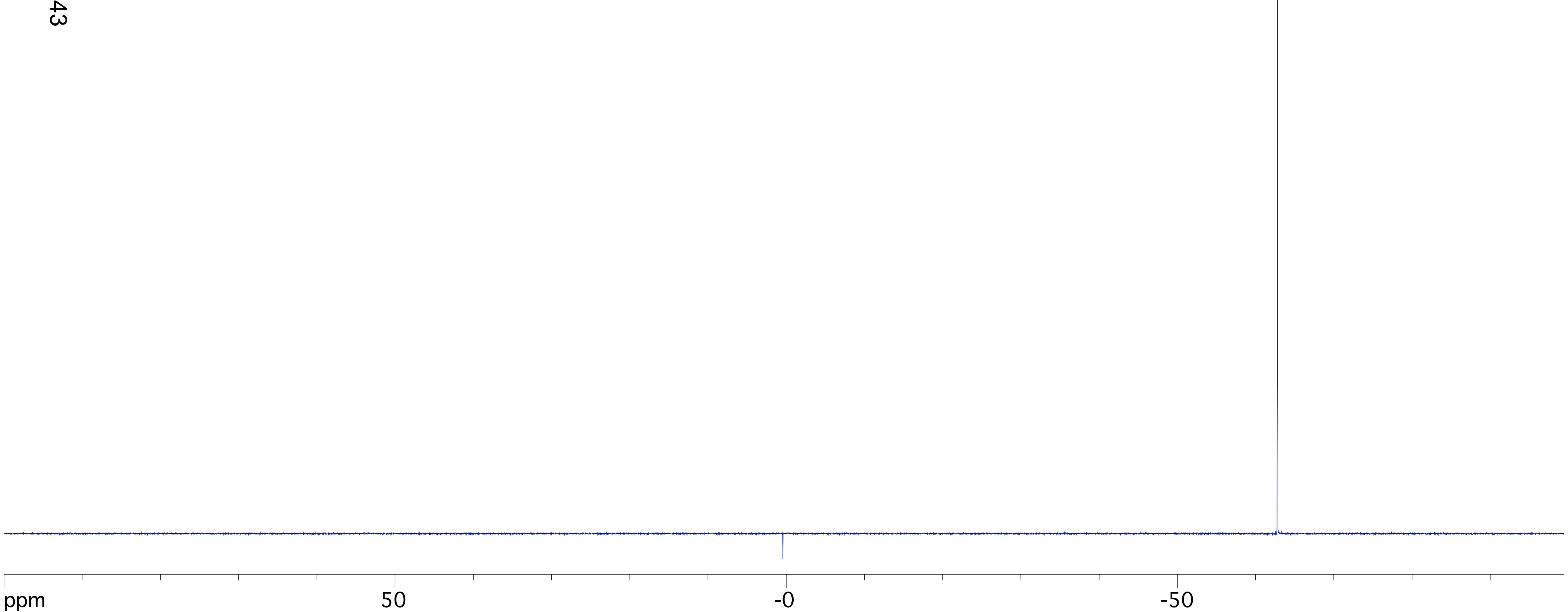

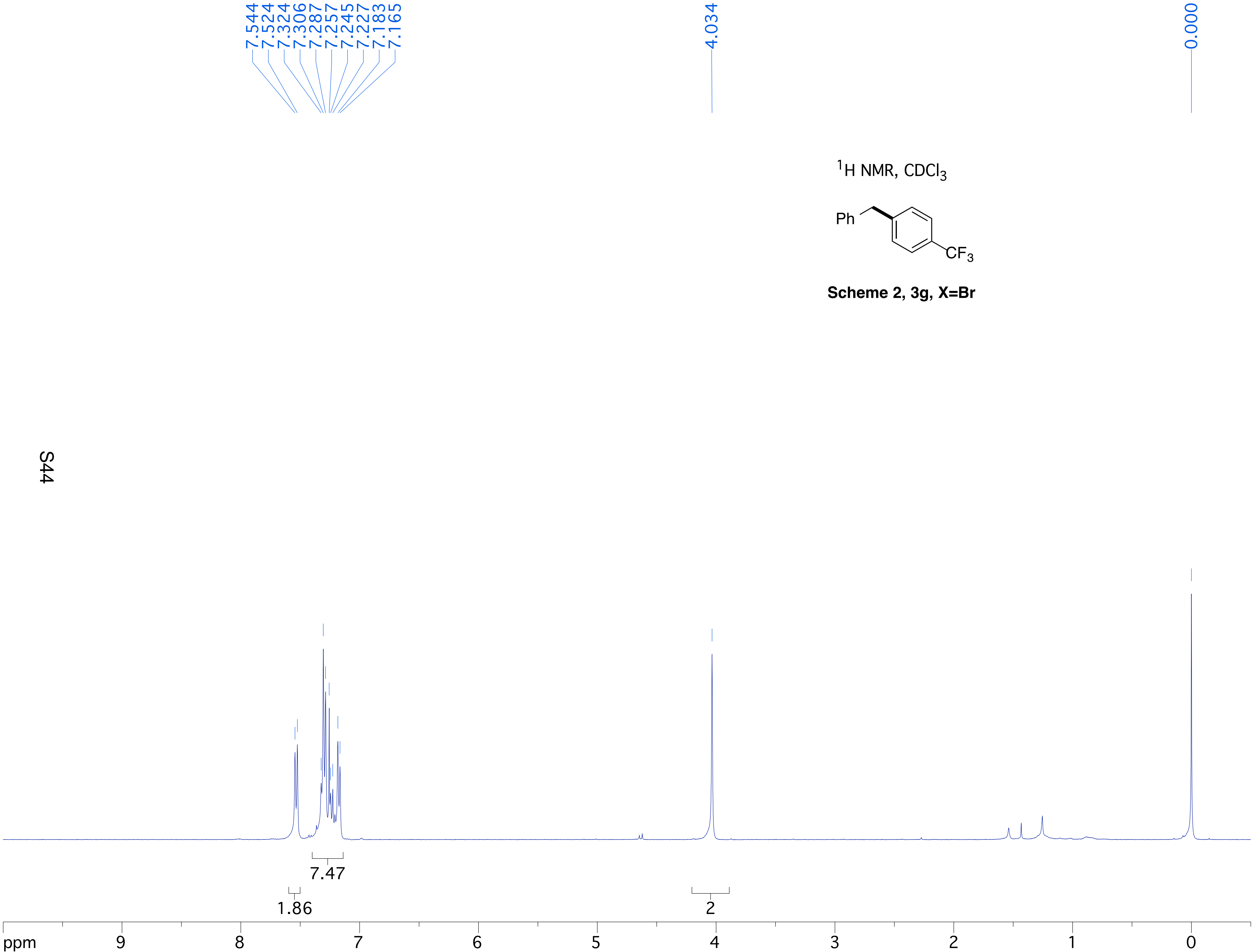
7.544
7.524
7.324
7.306
7.287
7.257
7.245
7.227
7.183
7.165
4.034
0.000
1H NMR, CDCl3
Ph
CF3
Scheme 2, 3g, X=Br
1.86
7.47
2
ppm
9
8
7
6
5
4
3
2
1
0

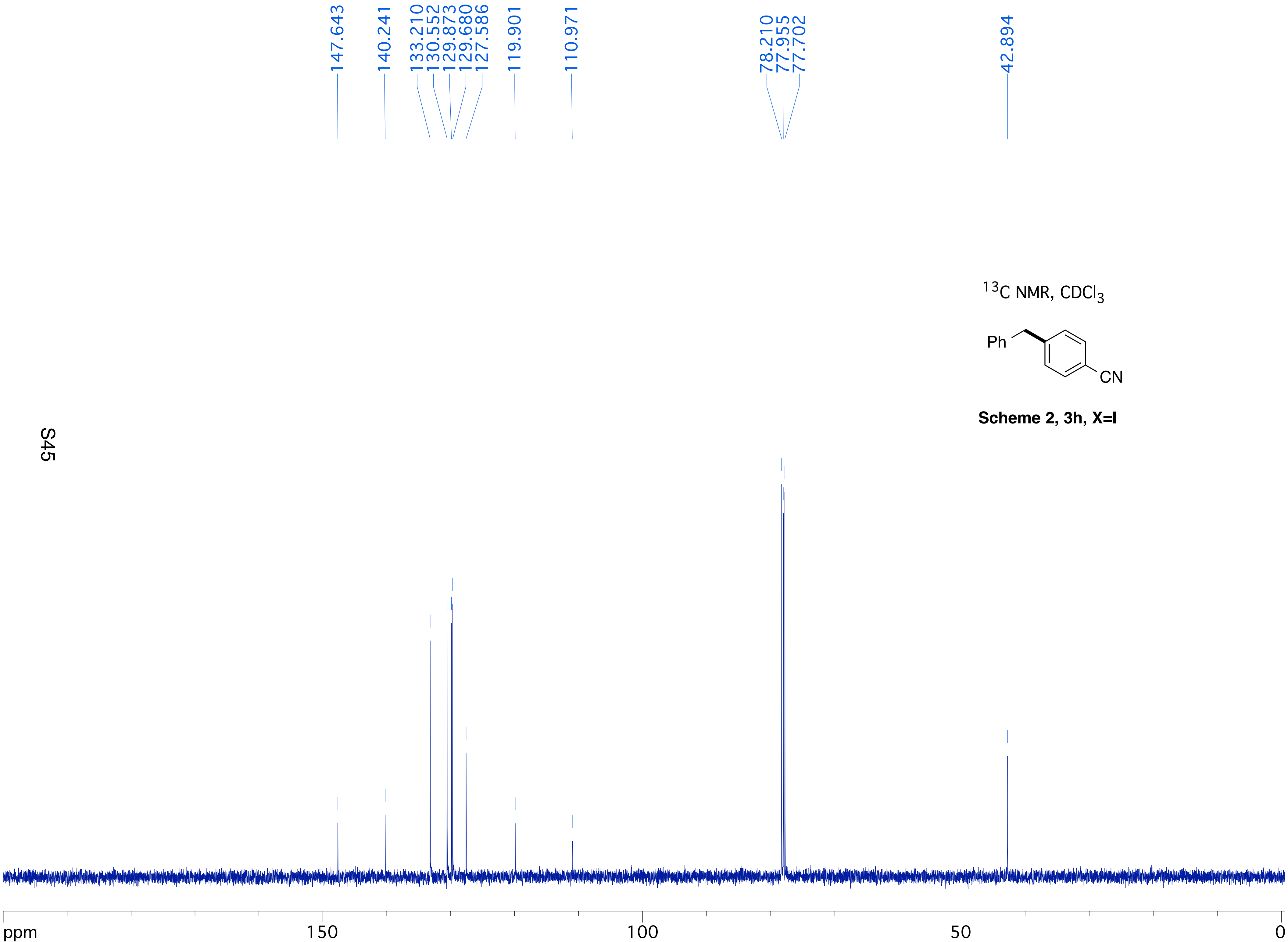

^{13}C NMR, $CDCl_3$

Scheme 2, 3h, X=I

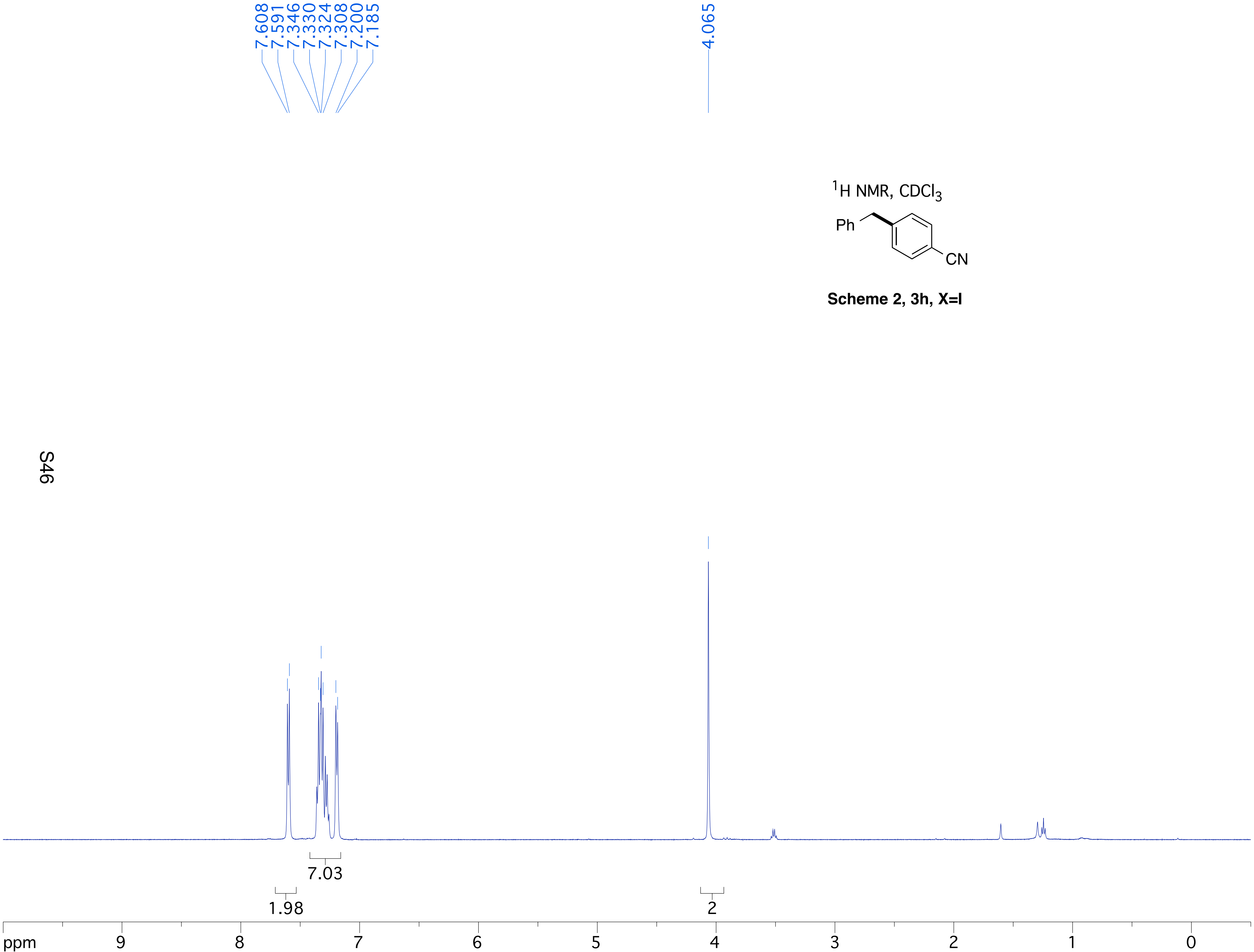

Scheme 2, 3h, X=I

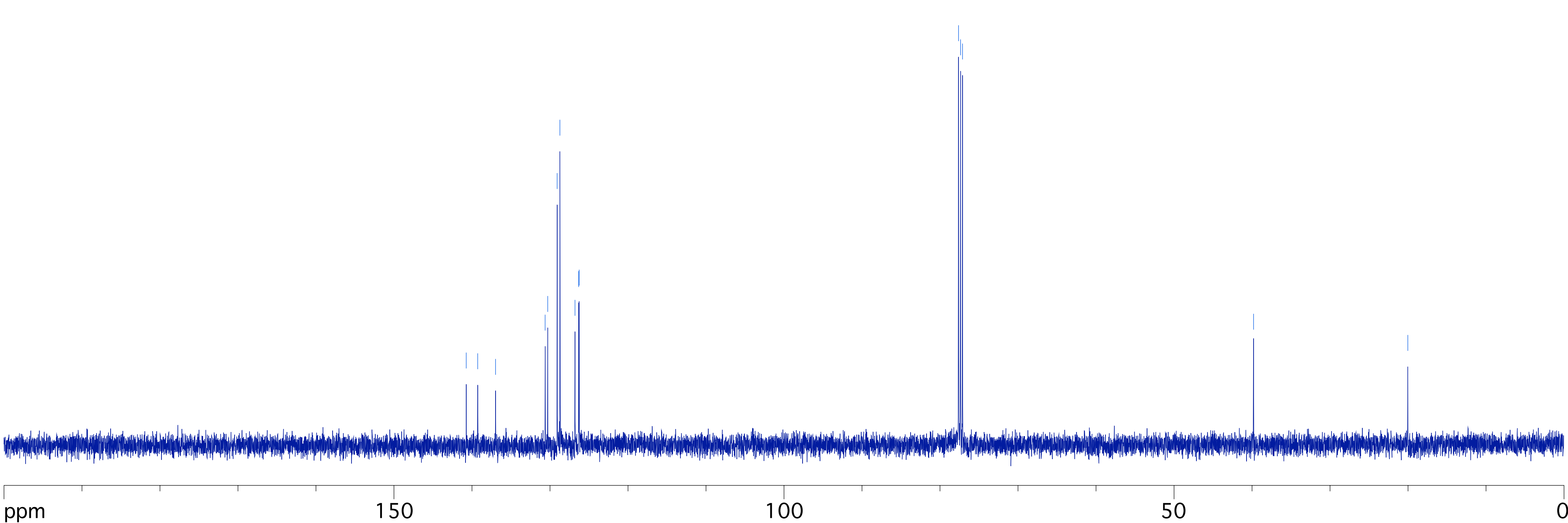

^{13}C NMR, $CDCl_3$

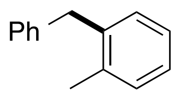

Scheme 2, 3i, X=Br

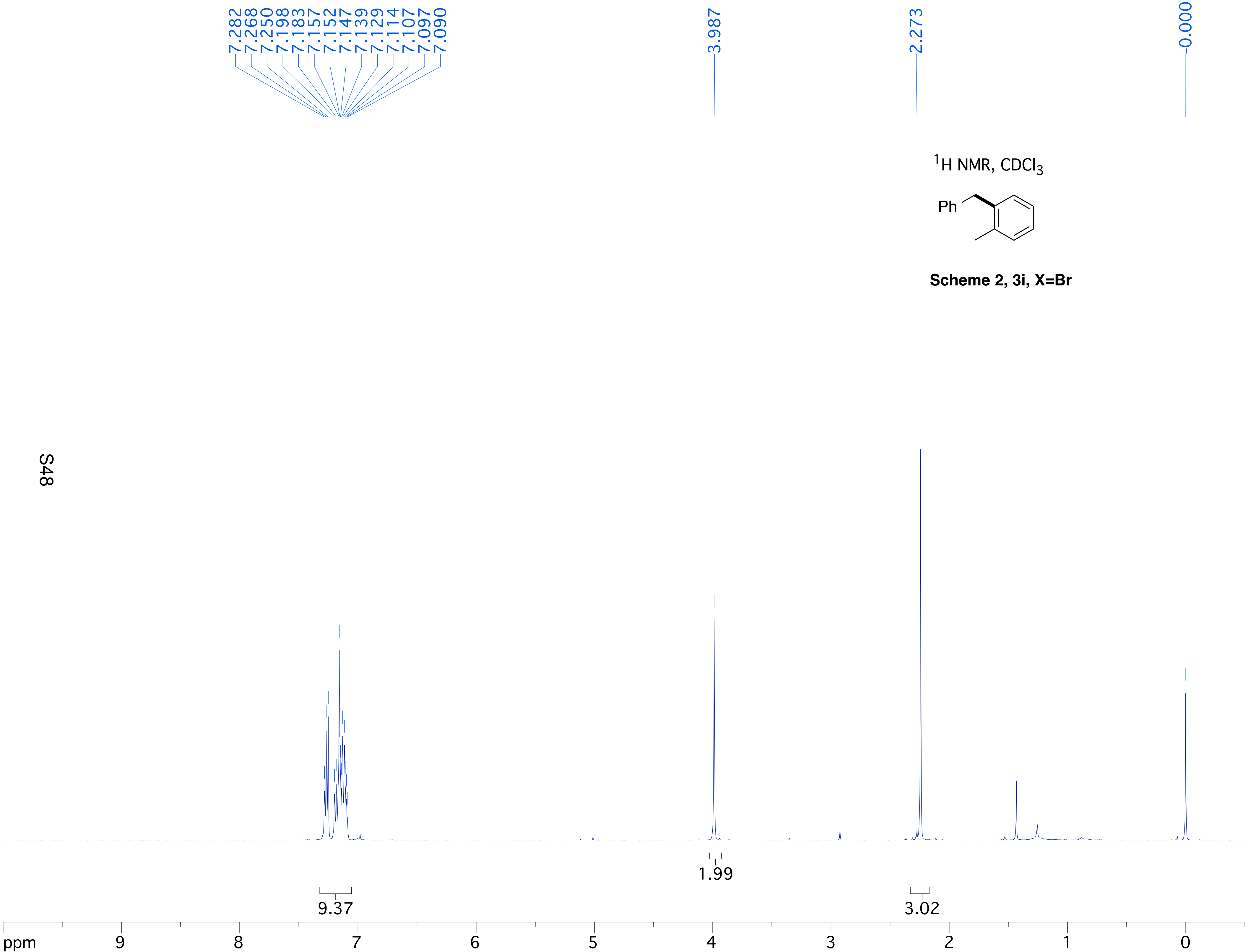

7.282
7.268
7.250
7.198
7.183
7.157
7.152
7.147
7.139
7.129
7.114
7.107
7.097
7.090
3.987
2.273
-0.000
^{1}H NMR, $CDCl_3$
Ph
Scheme 2, 3i, X=Br
9.37
1.99
3.02
ppm
9
8
7
6
5
4
3
2
1
0

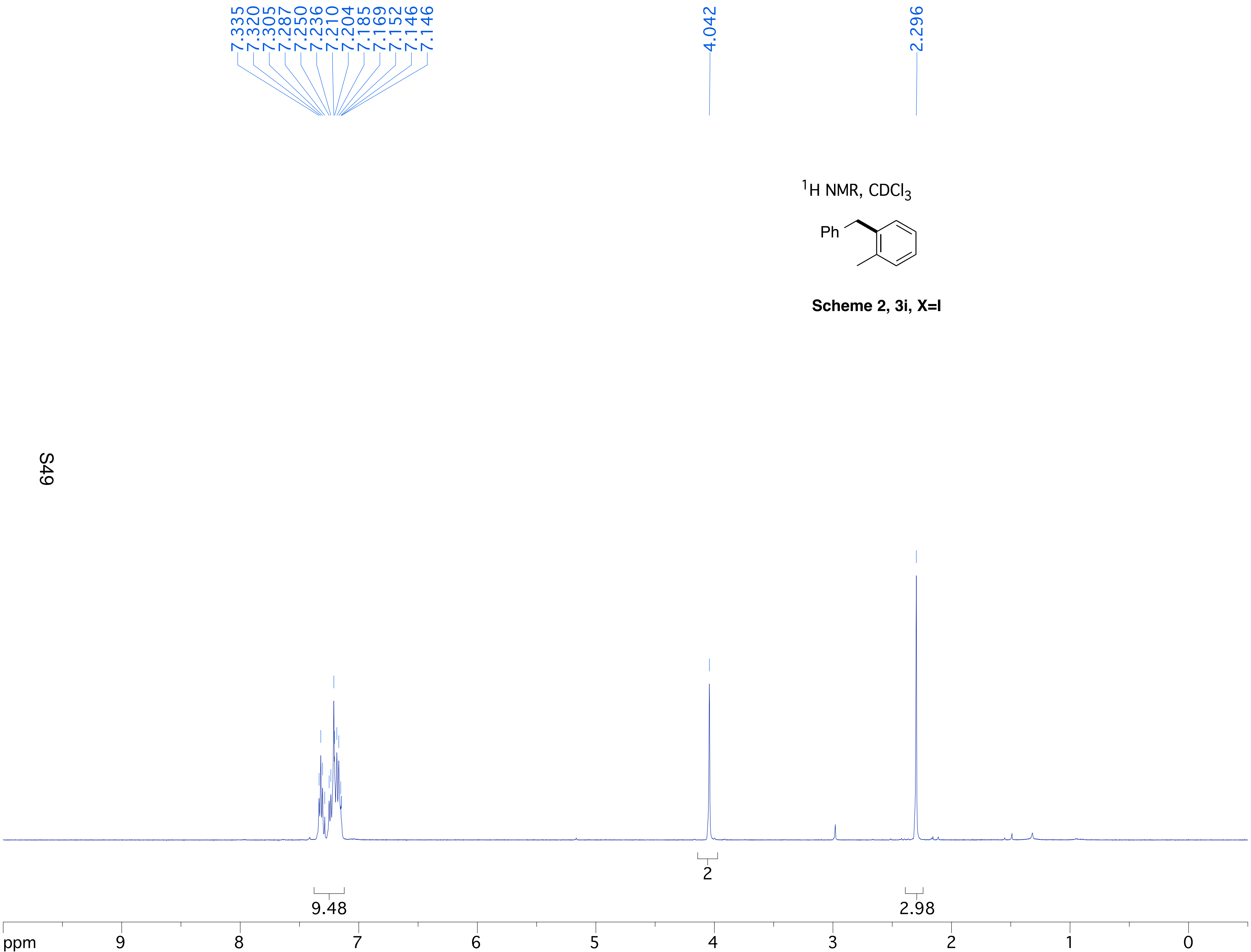
7.335
7.320
7.305
7.287
7.250
7.236
7.210
7.204
7.185
7.169
7.152
7.146
7.146
4.042
2.296
1H NMR, CDCl3
Ph
Scheme 2, 3i, X=I
2
9.48
2.98
ppm
9
8
7
6
5
4
3
2
1
0

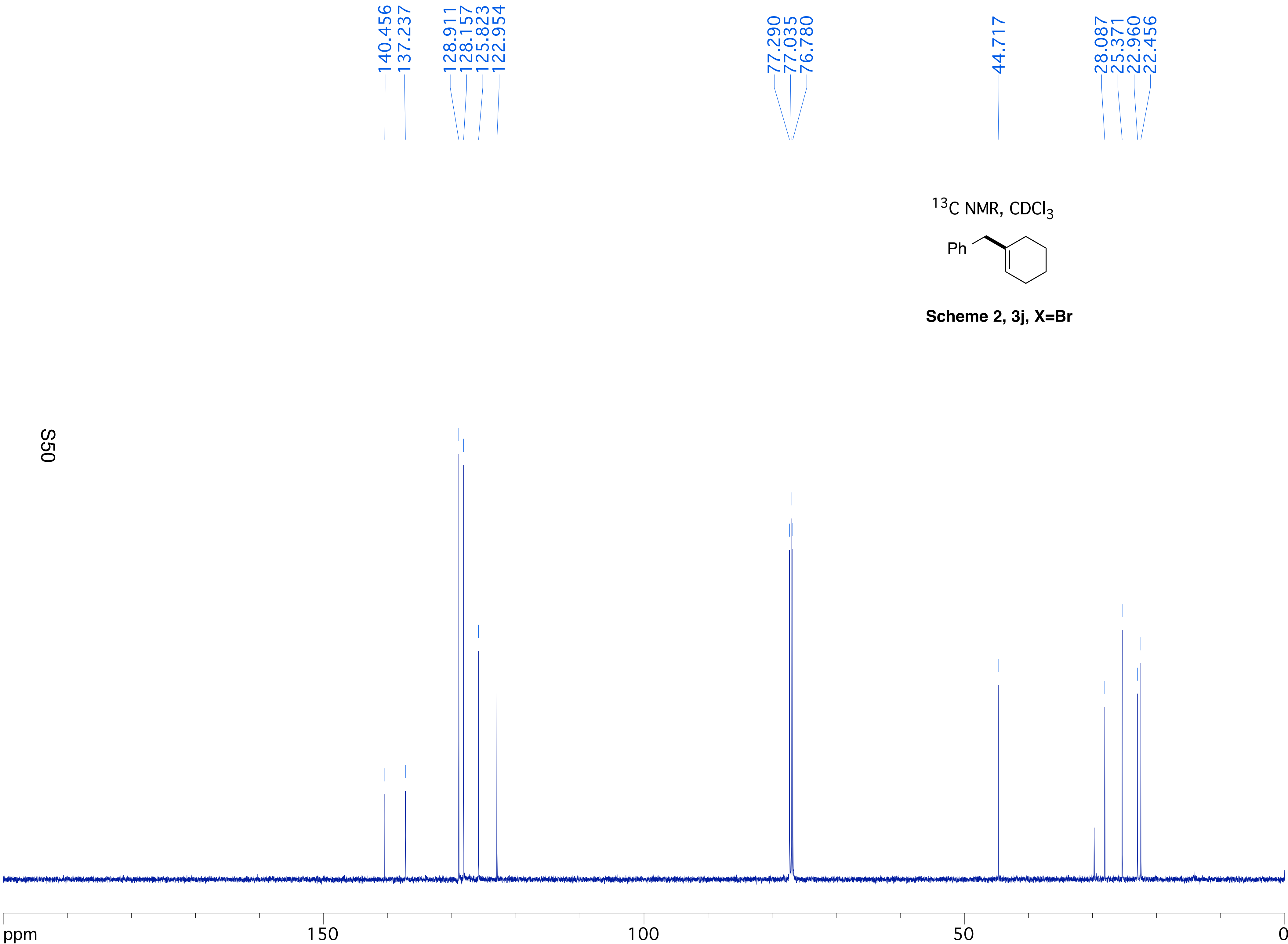
140.456
137.237
128.911
128.157
125.823
122.954
77.290
77.035
76.780
44.717
28.087
25.371
22.960
22.456
13C NMR, CDCl3
Ph
Scheme 2, 3j, X=Br
ppm
150
100
50
0

^{1}H NMR, $CDCl_3$

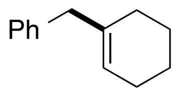

Scheme 2, 3j, X=Br

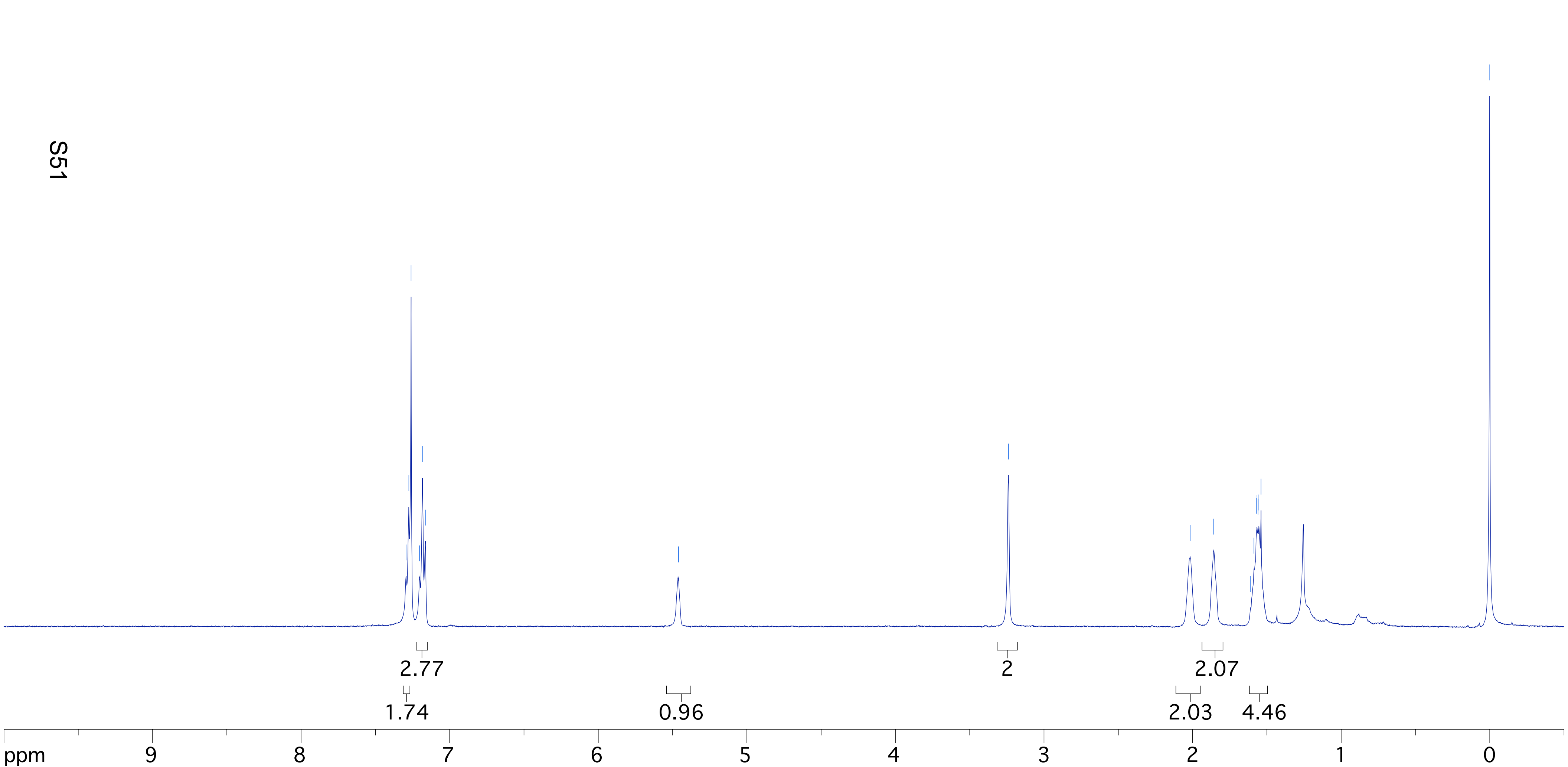

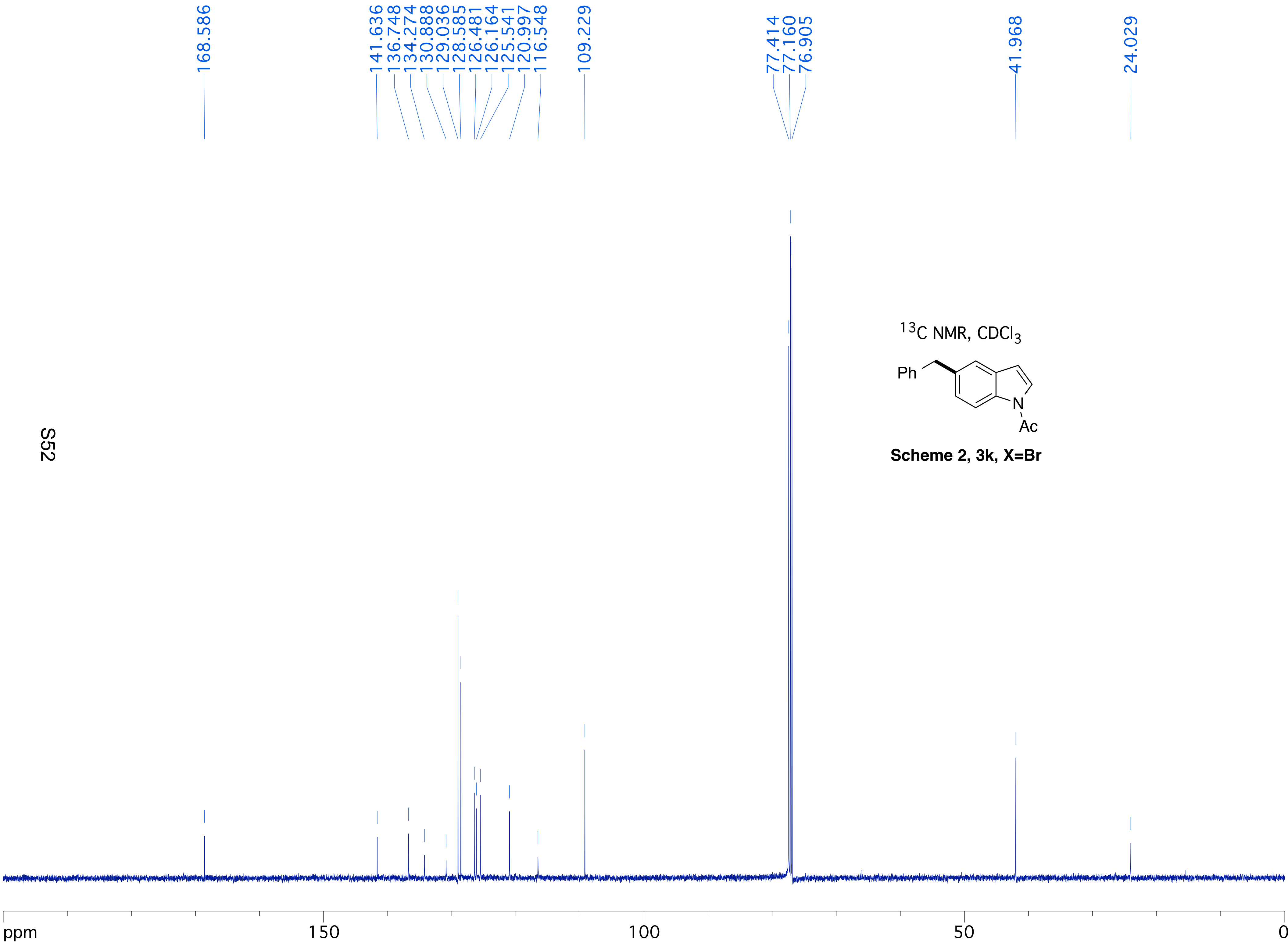
168.586
141.636
136.748
134.274
130.888
129.036
128.585
126.481
126.164
125.541
120.997
116.548
109.229
77.414
77.160
76.905
41.968
24.029
^{13}C NMR, $CDCl_3$
Ph
N
Ac
Scheme 2, 3k, X=Br
ppm
150
100
50
0

^{1}H NMR, $CDCl_3$

Scheme 2, 3k, X=Br

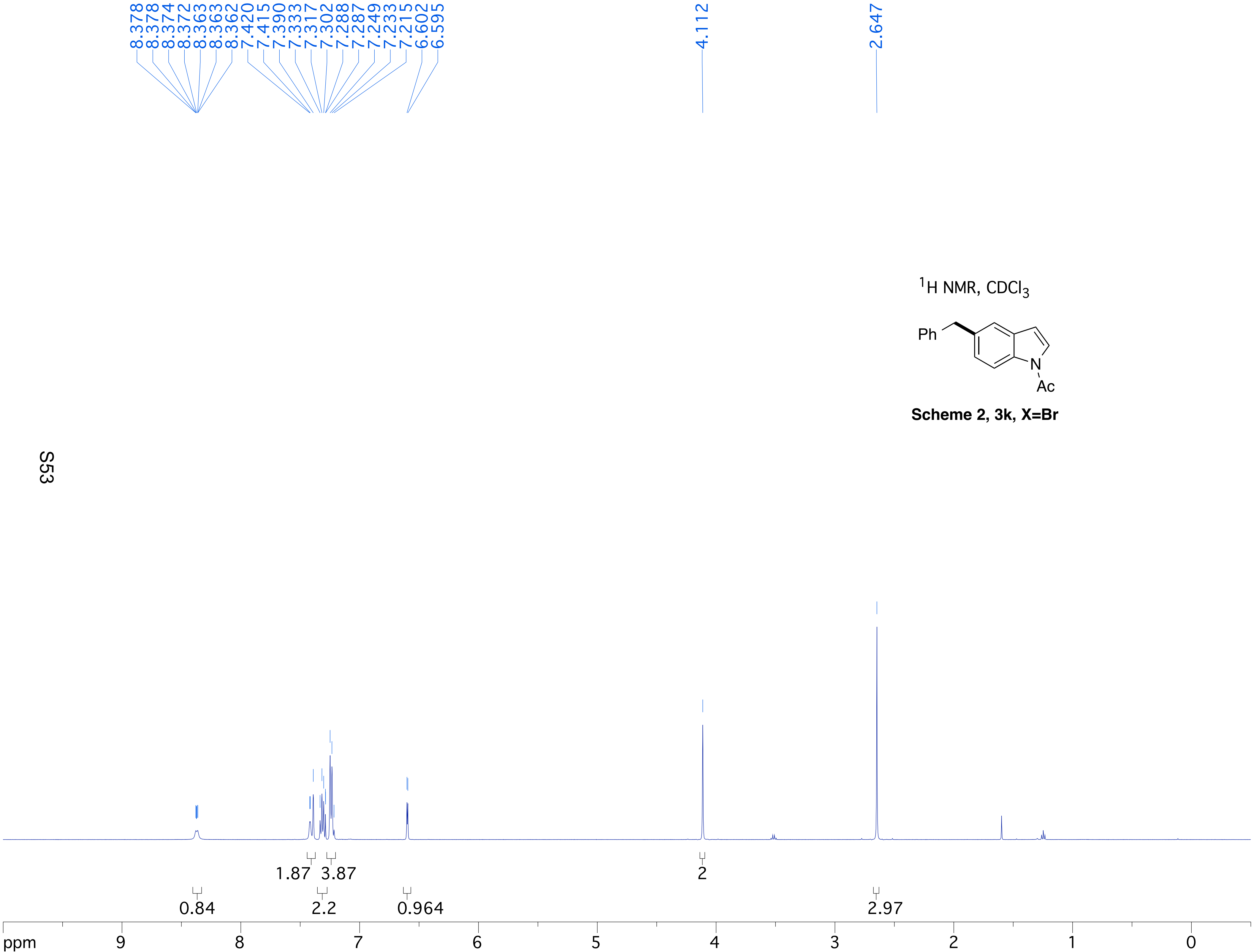

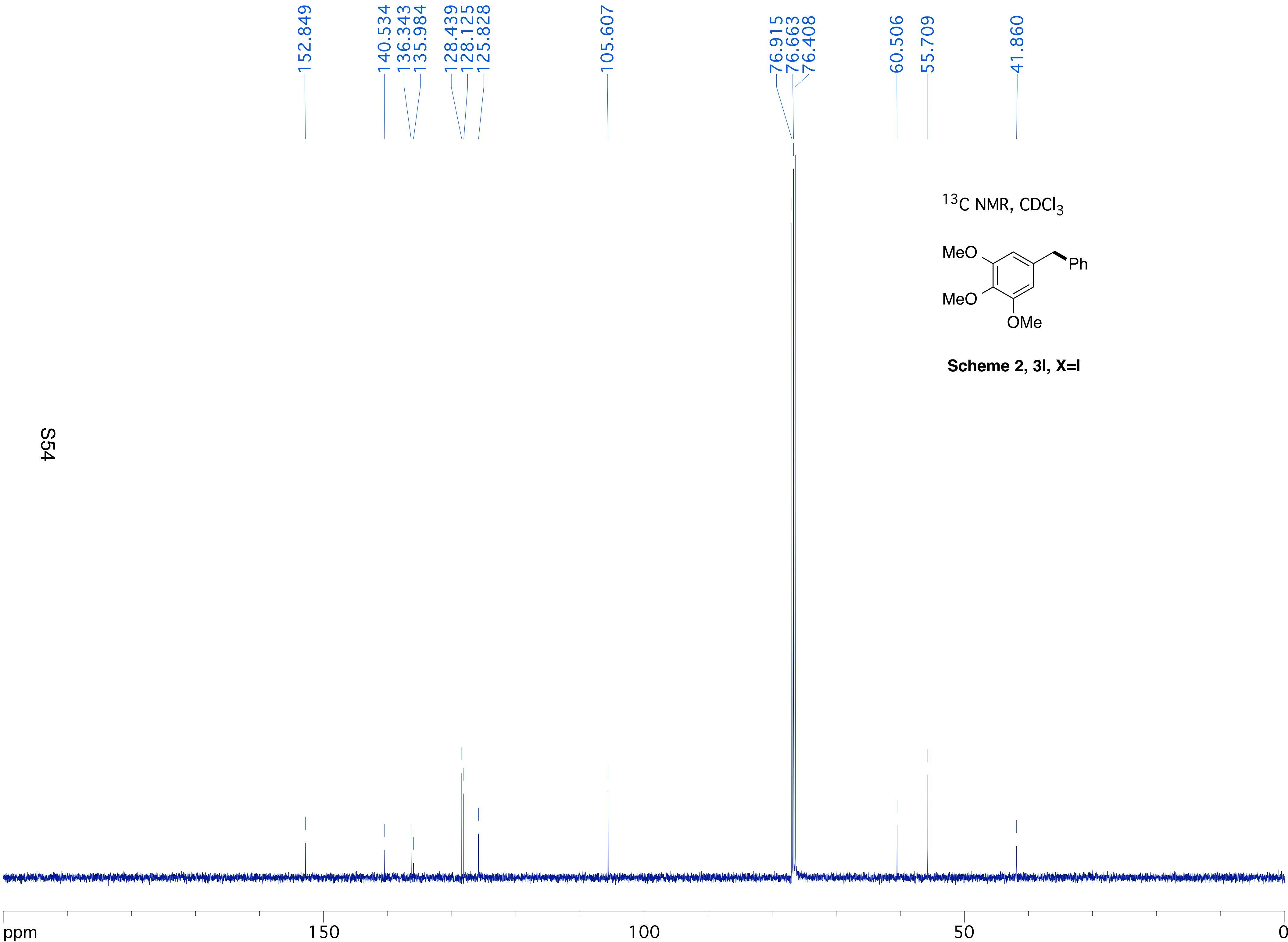

^{13}C NMR, $CDCl_3$

Scheme 2, 3l, X=I

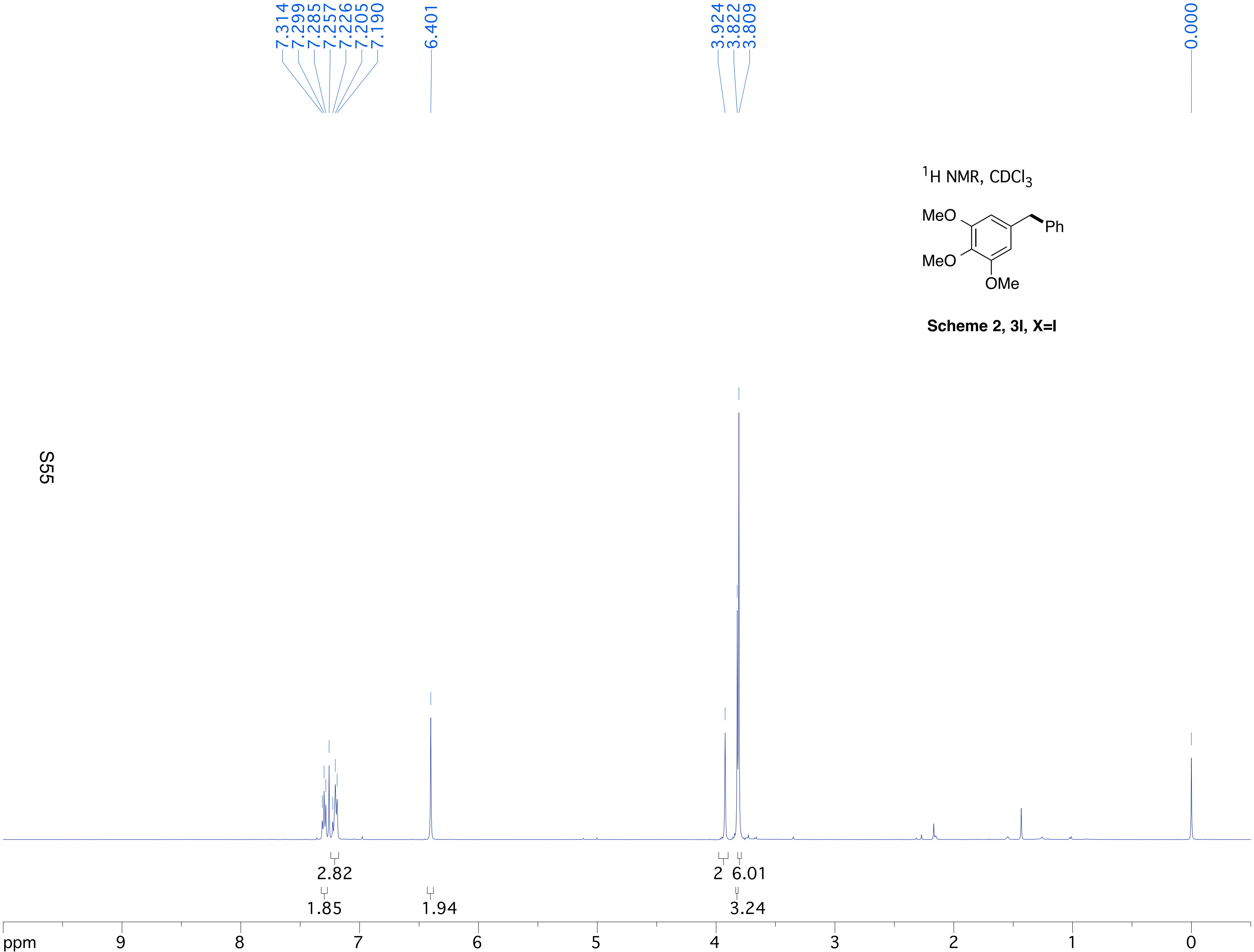
7.314
7.299
7.285
7.257
7.226
7.205
7.190
6.401
3.924
3.822
3.809
0.000
^{1}H NMR, $CDCl_3$
MeO
MeO
OMe
Ph
Scheme 2, 3l, X=I
2.82
1.85
1.94
2
6.01
3.24
ppm
9
8
7
6
5
4
3
2
1
0

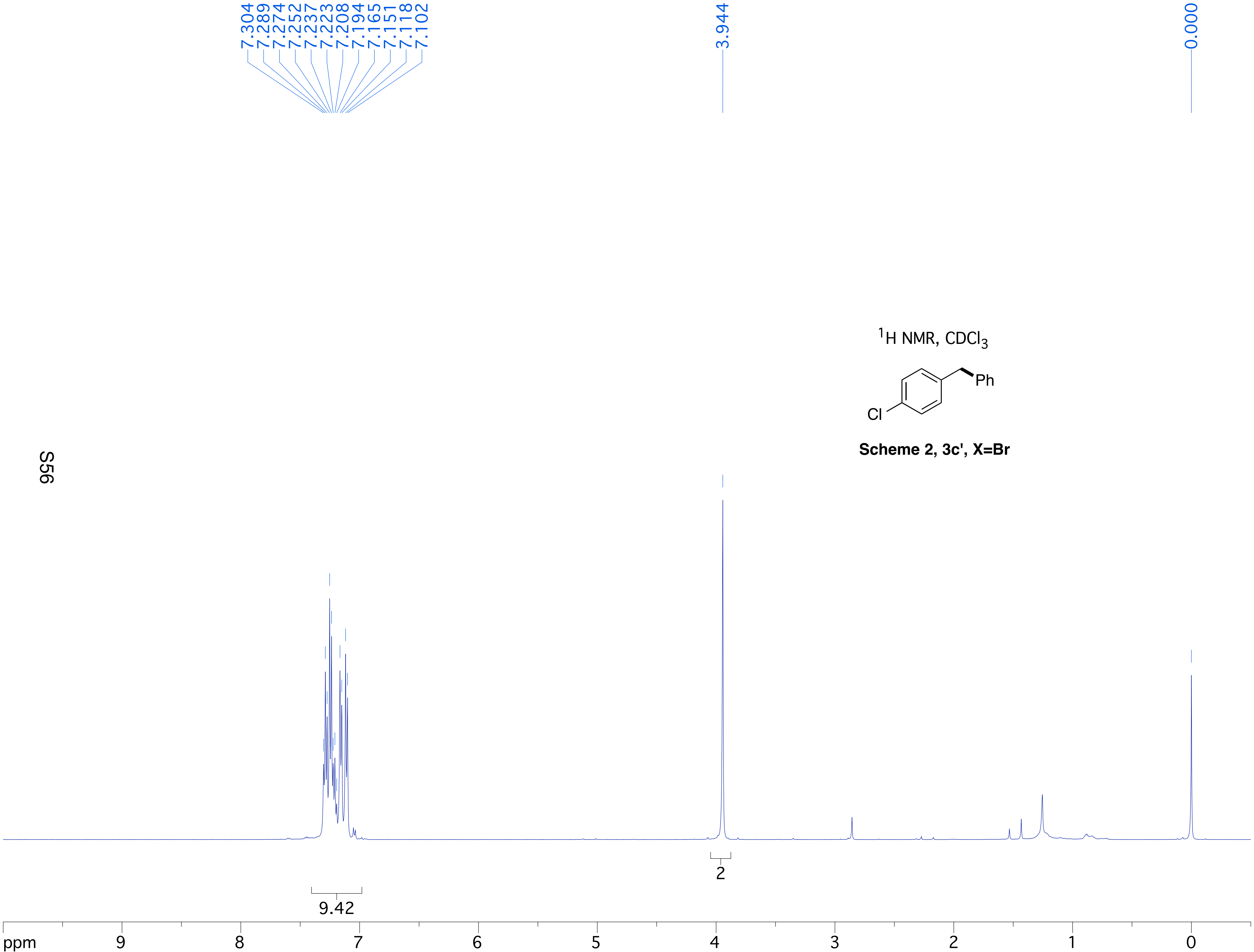
7.304
7.289
7.274
7.252
7.237
7.223
7.208
7.194
7.165
7.151
7.118
7.102
3.944
0.000
^{1}H NMR, $CDCl_3$
Ph
Cl
Scheme 2, 3c', X=Br
9.42
2
ppm
9
8
7
6
5
4
3
2
1
0

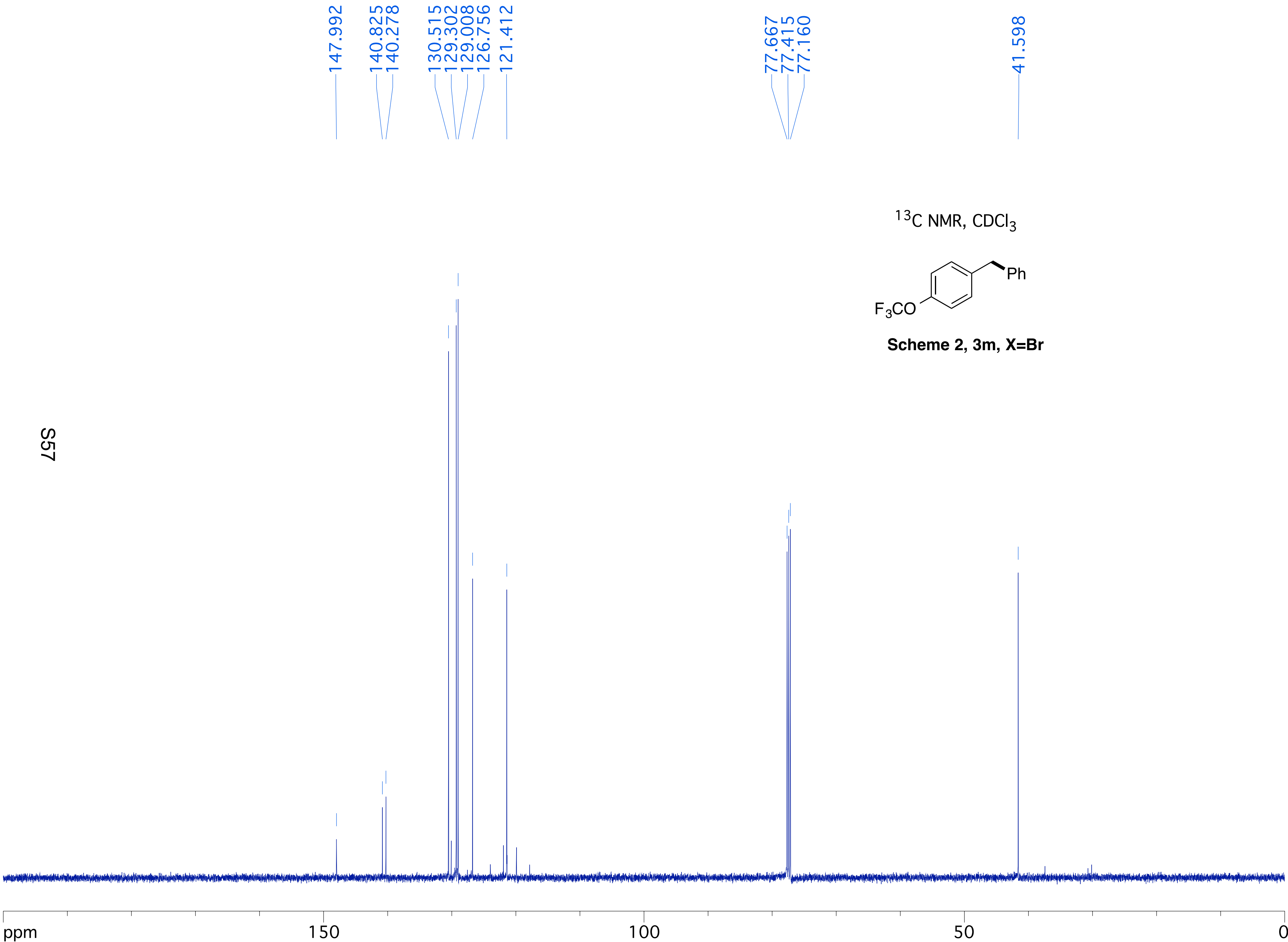

147.992
140.825
140.278
130.515
129.302
129.008
126.756
121.412
77.667
77.415
77.160
41.598
13C NMR, CDCl3
F3CO
Ph
Scheme 2, 3m, X=Br
ppm
150
100
50
0

^{19}F NMR, $CDCl_3$

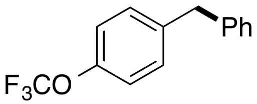

Scheme 2, 3m, X=Br

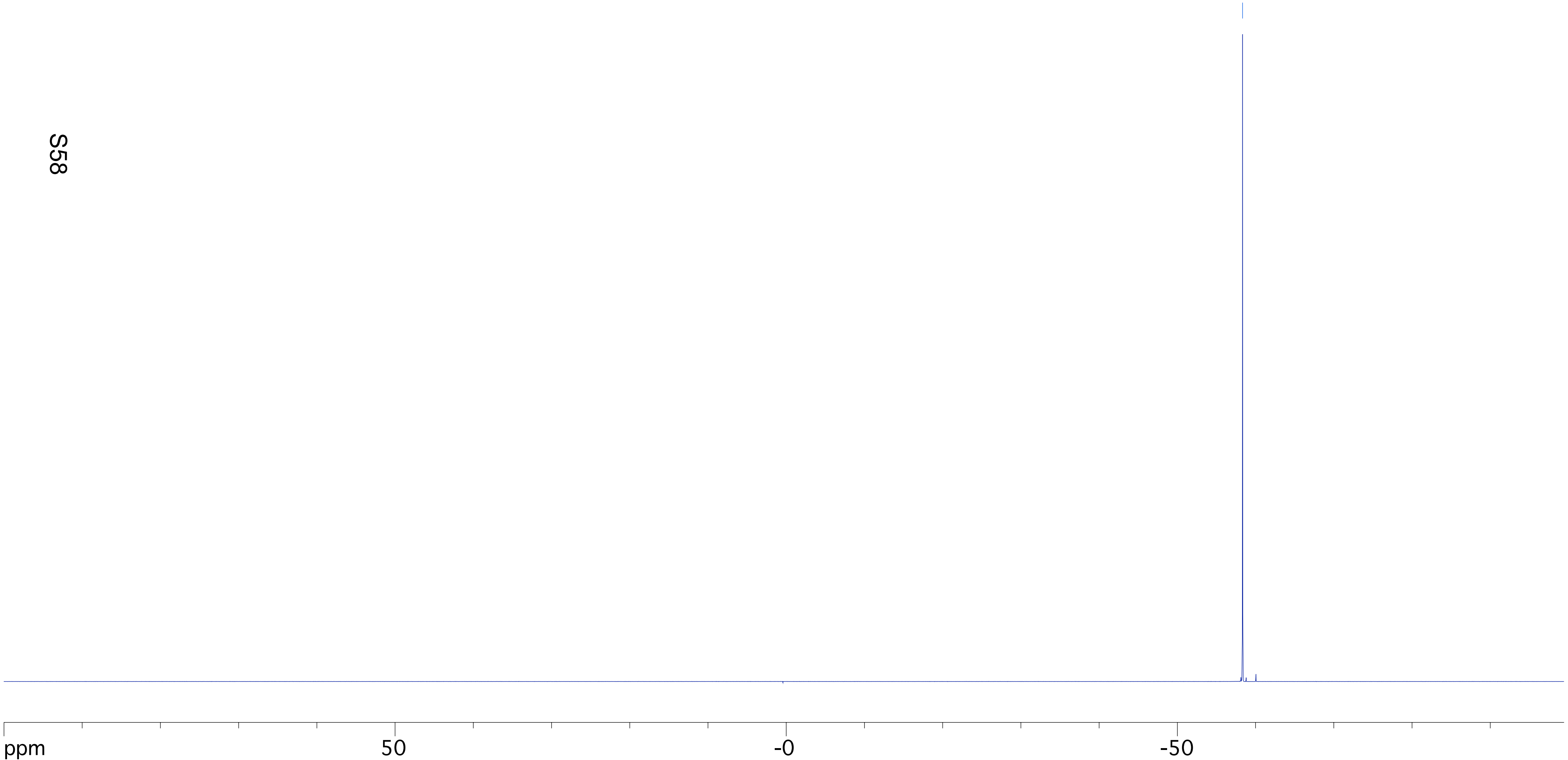

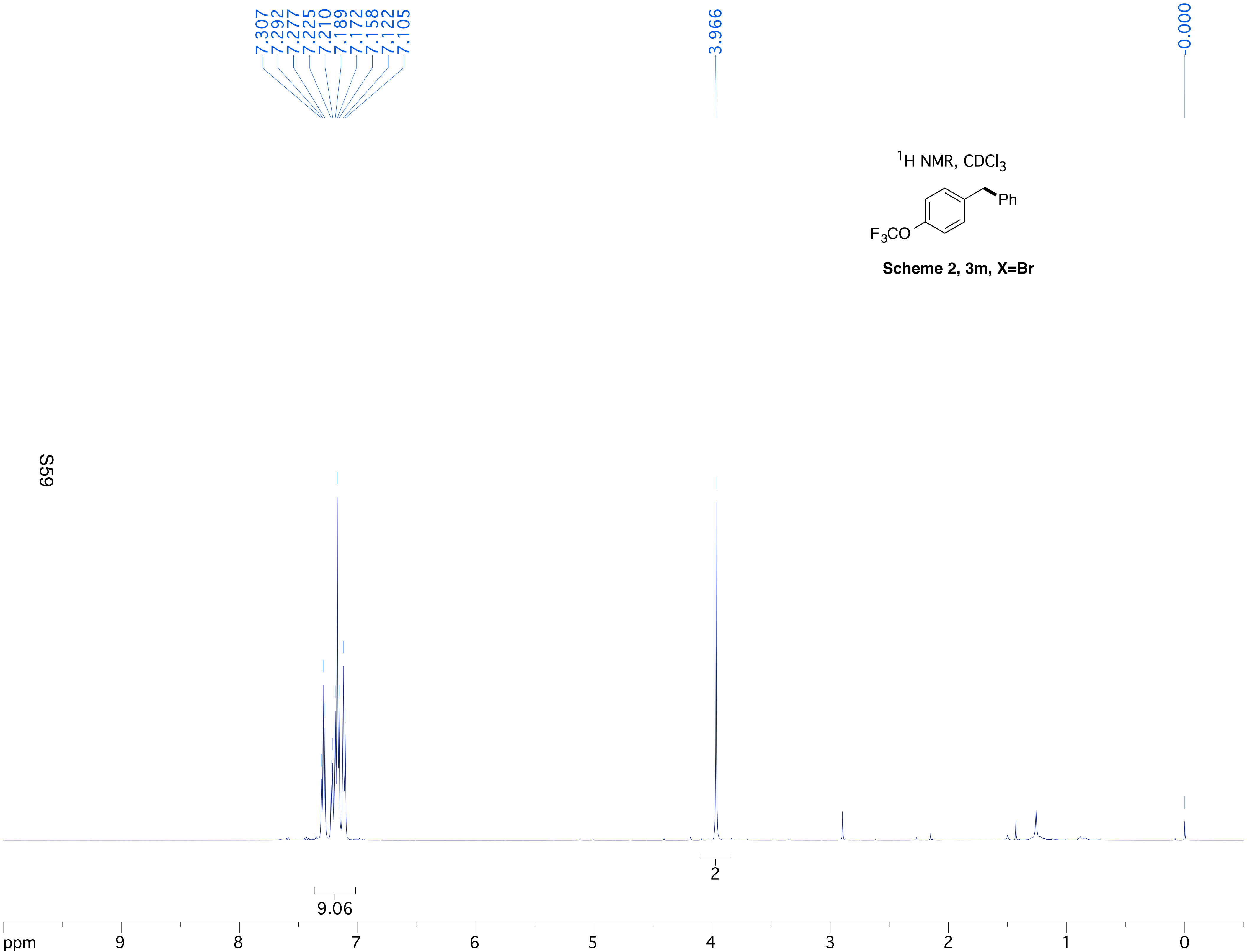

Scheme 2, 3m, X=Br

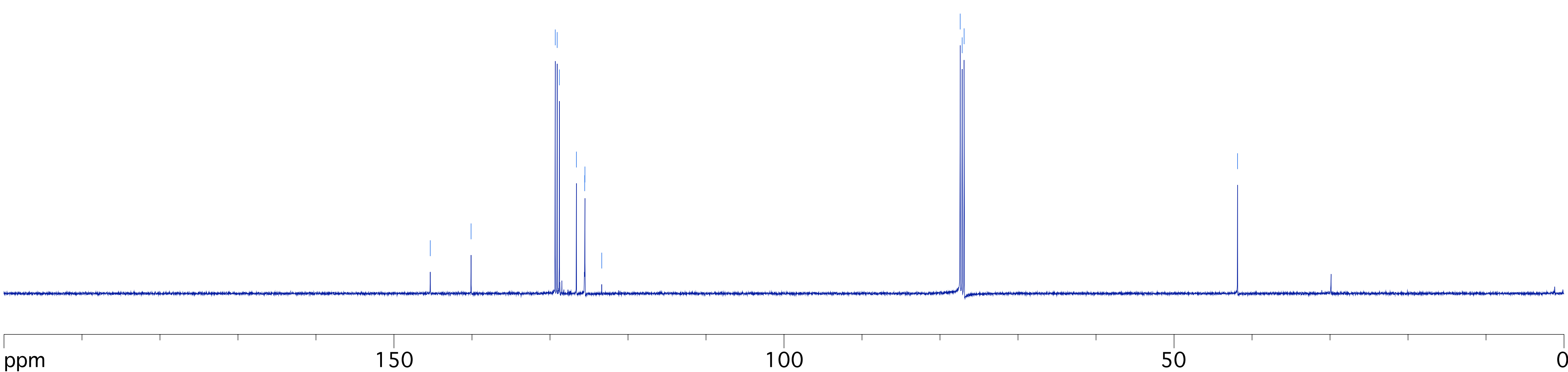

^{13}C NMR, $CDCl_3$

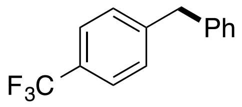

Scheme 2, 3g', X=Br

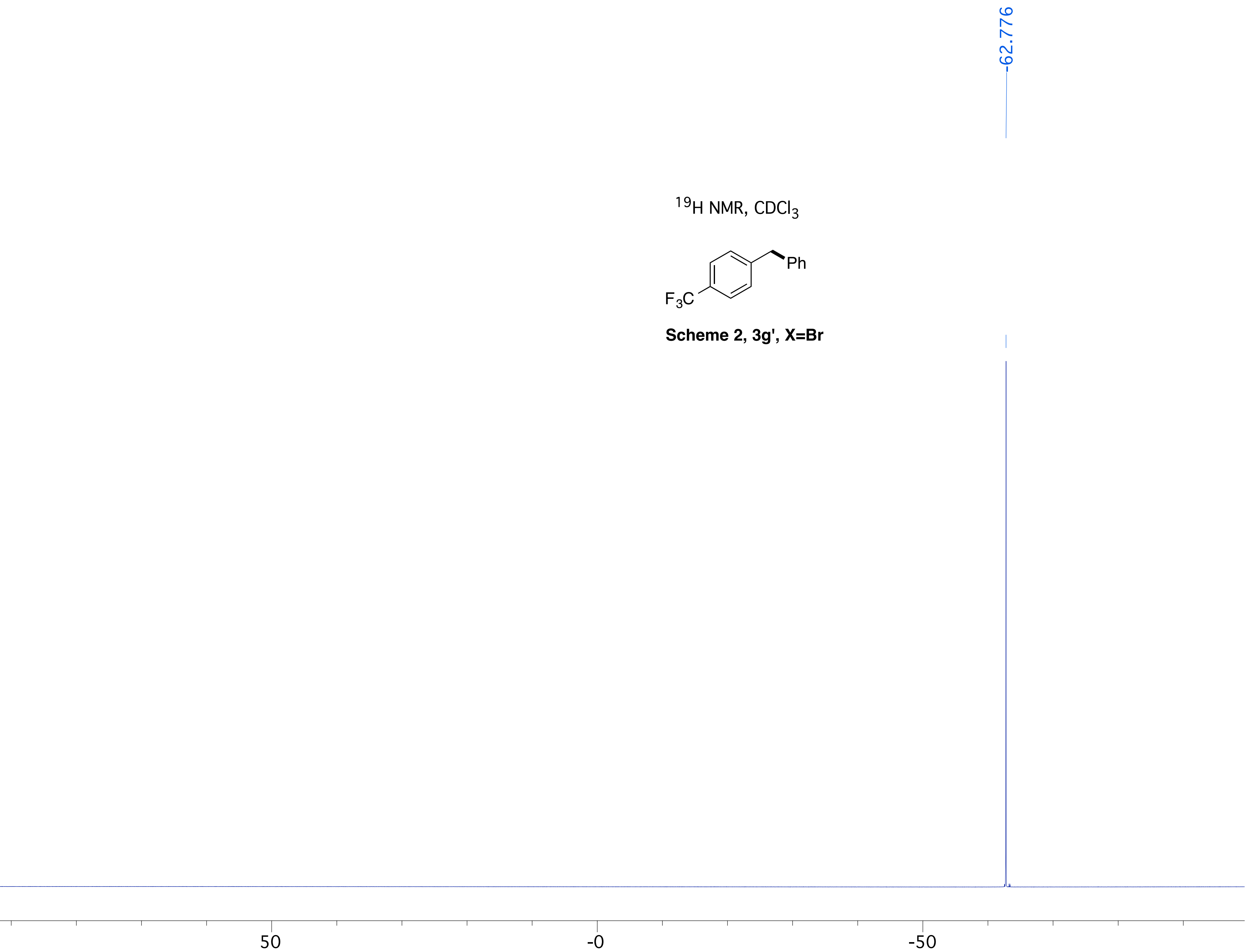
-62.776
^{19}H NMR, $CDCl_3$
F_3C
Ph
Scheme 2, 3g', X=Br
ppm
50
-0
-50

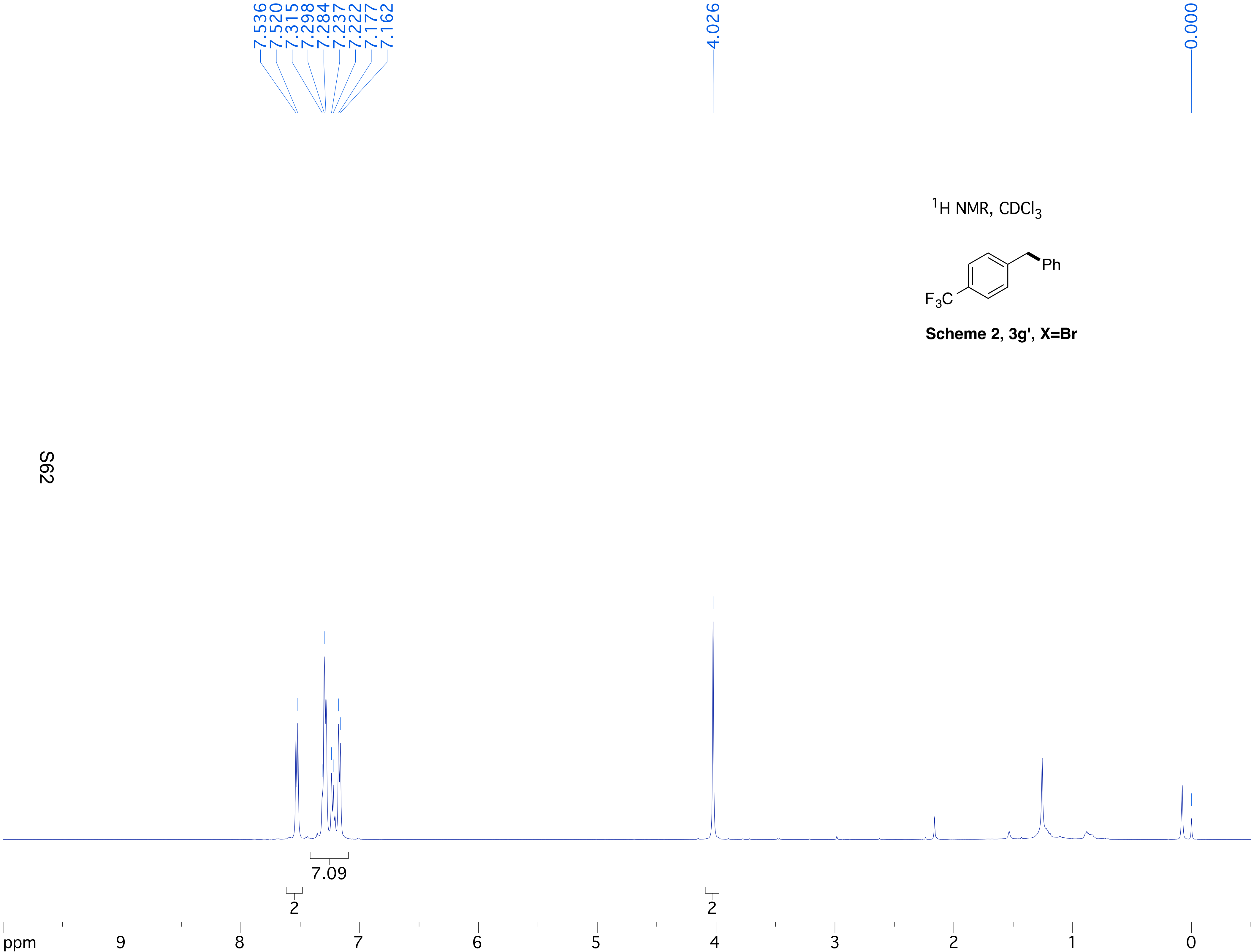

7.536
7.520
7.315
7.298
7.284
7.237
7.222
7.177
7.162
4.026
0.000
^{1}H NMR, $CDCl_3$
F_3C
Ph
Scheme 2, 3g', X=Br
7.09
2
2
ppm
9
8
7
6
5
4
3
2
1
0

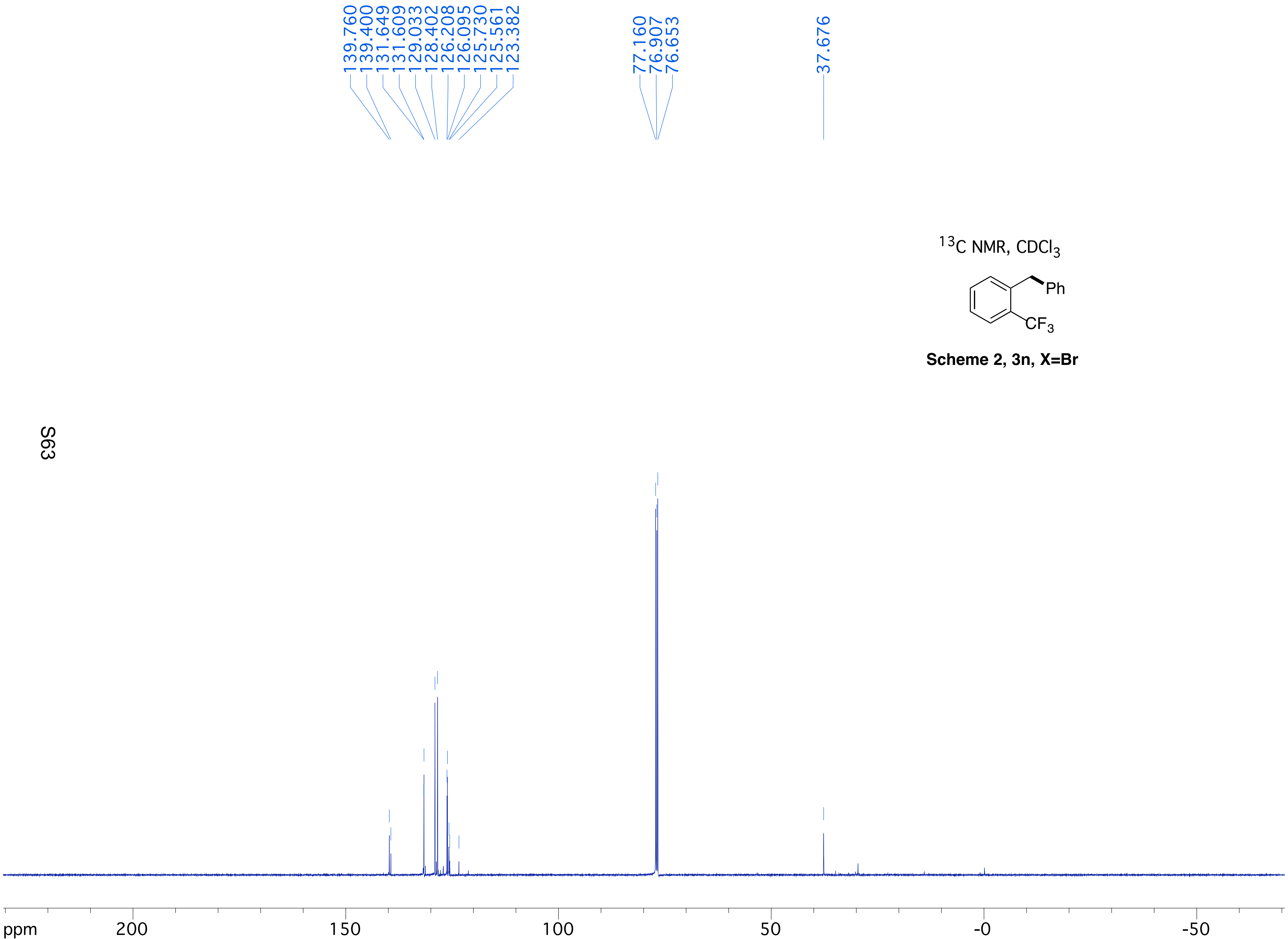

^{13}C NMR, $CDCl_3$

Scheme 2, 3n, X=Br

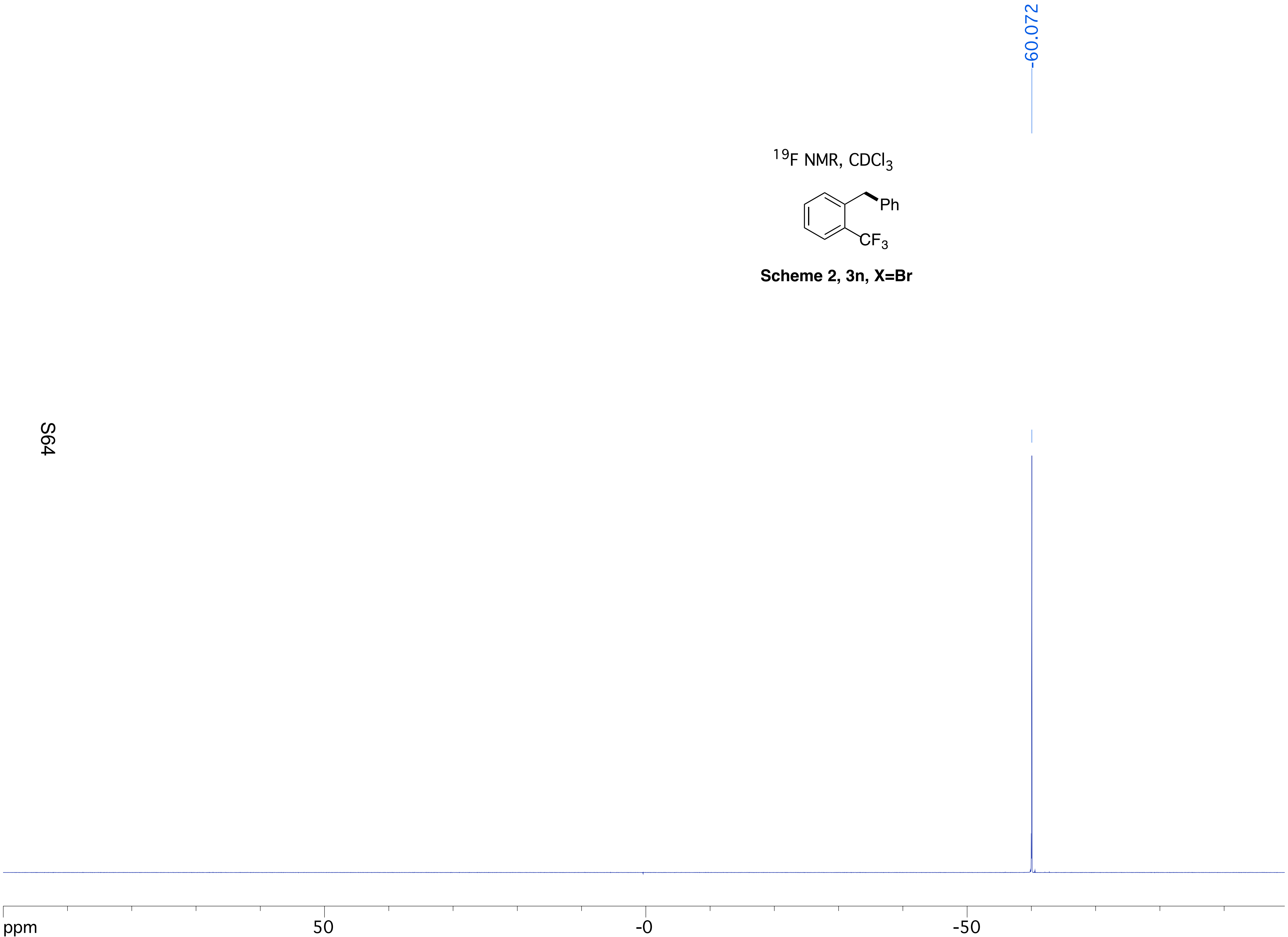

-60.072
19F NMR, CDCl3
Ph
CF3
Scheme 2, 3n, X=Br
ppm
50
-0
-50

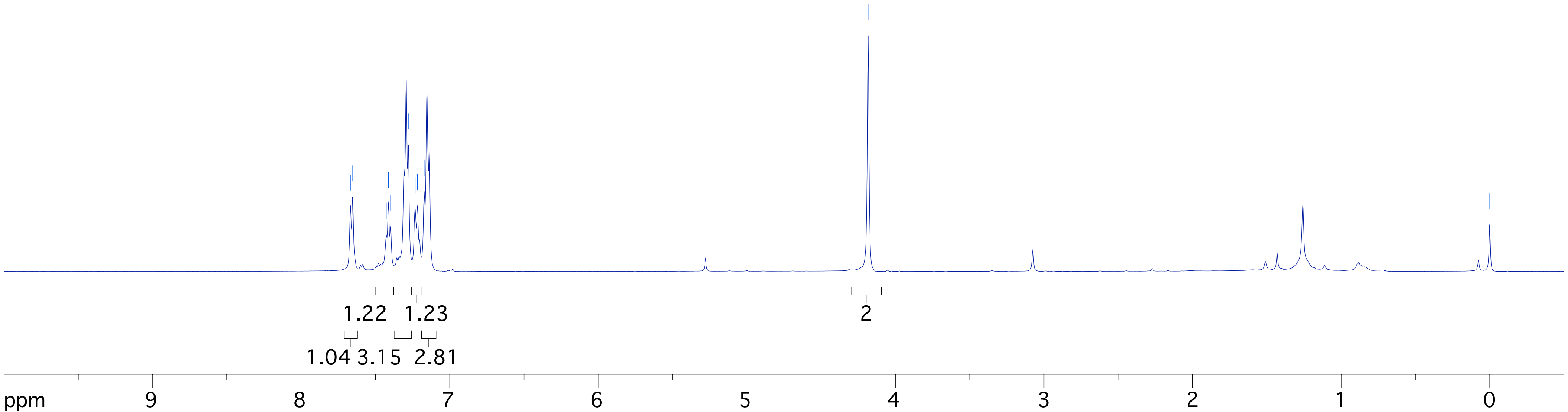

^1H NMR, $CDCl_3$

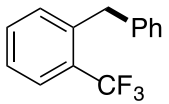

Scheme 2, 3n, X=Br

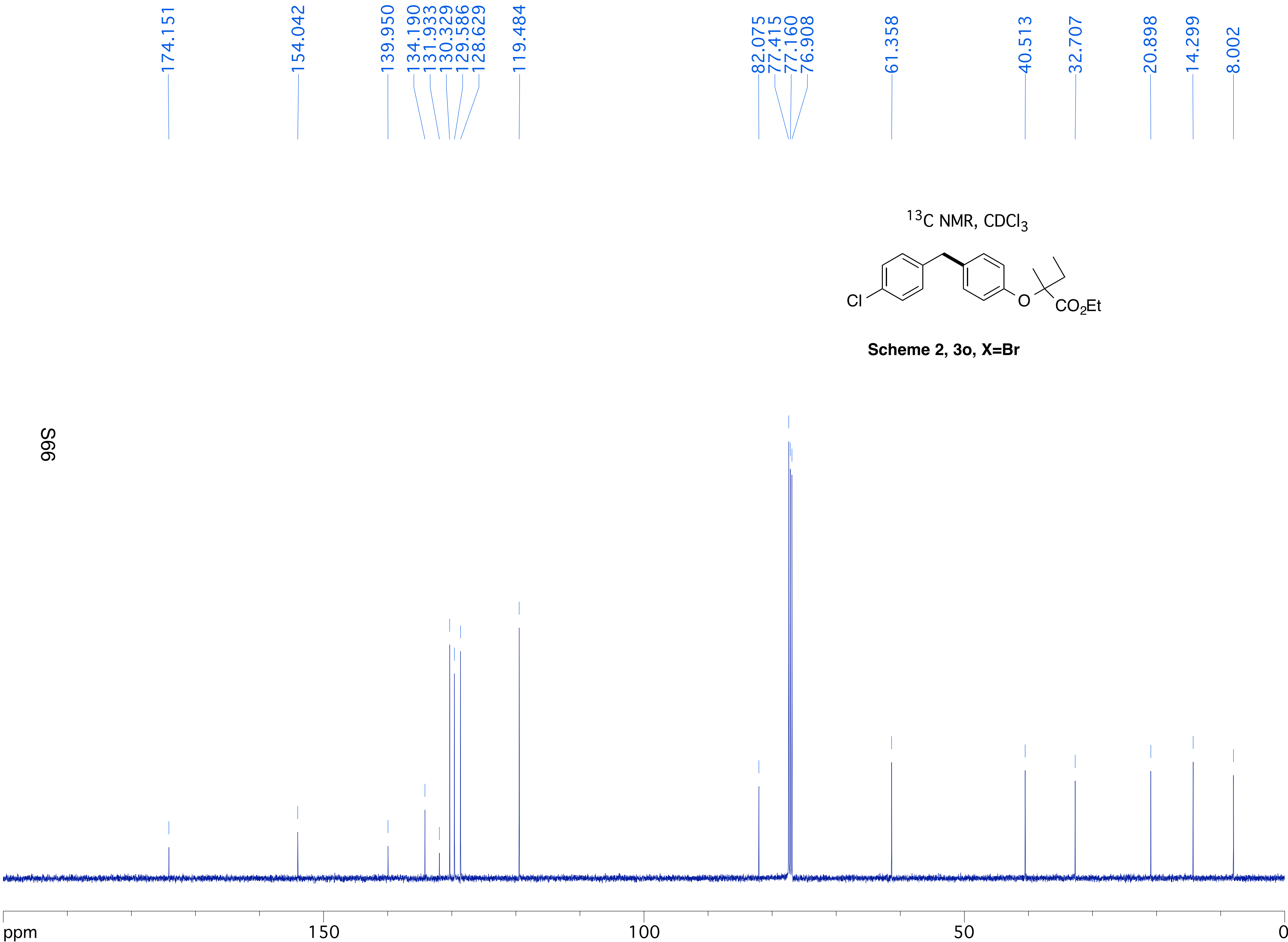
174.151
154.042
139.950
134.190
131.933
130.329
129.586
128.629
119.484
82.075
77.415
77.160
76.908
61.358
40.513
32.707
20.898
14.299
8.002
^{13}C NMR, $CDCl_3$
Cl
O
CO_2Et
Scheme 2, 3o, X=Br
ppm
150
100
50
0

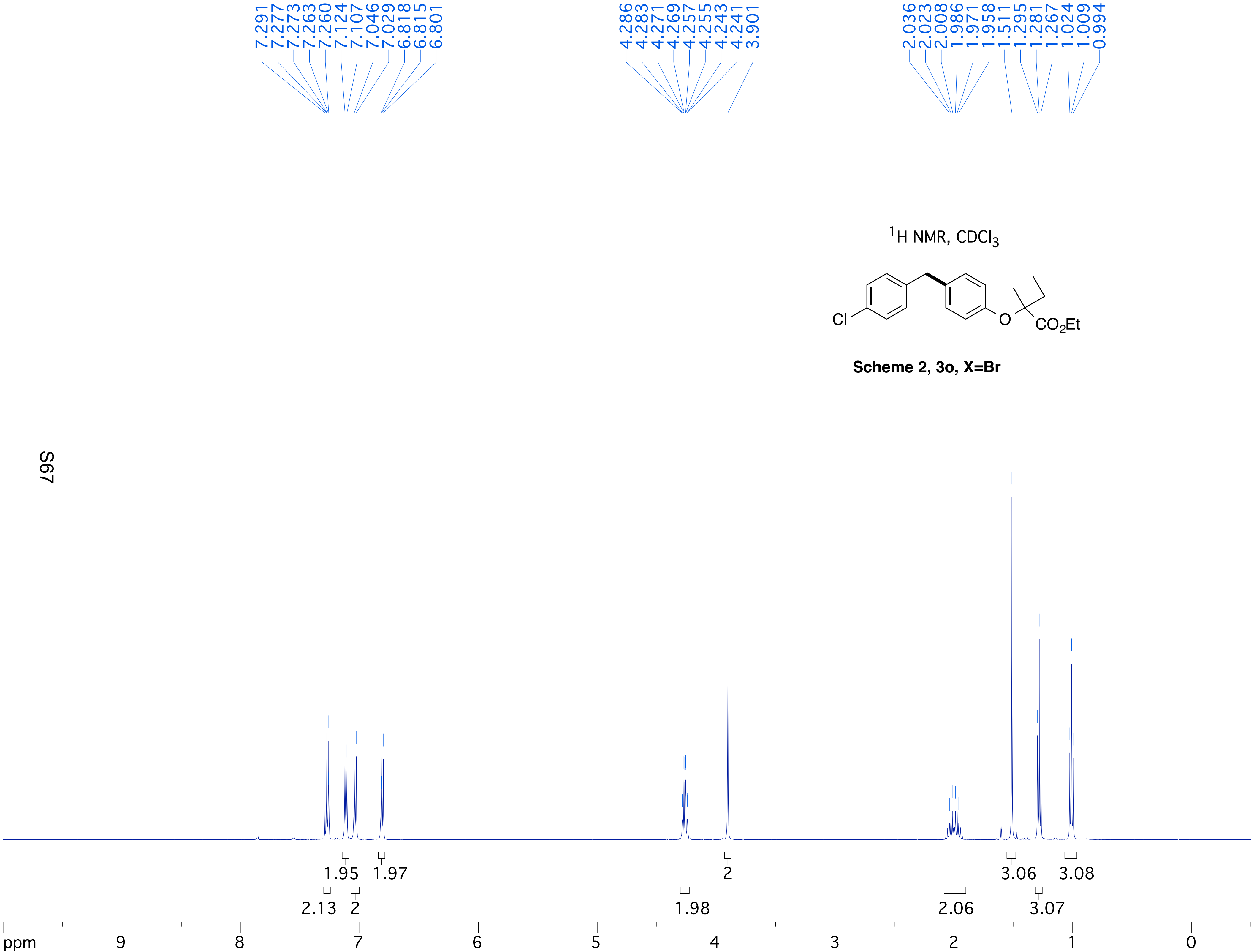
7.291
7.277
7.273
7.263
7.260
7.124
7.107
7.046
7.029
6.818
6.815
6.801
4.286
4.283
4.271
4.269
4.257
4.255
4.243
4.241
3.901
2.036
2.023
2.008
1.986
1.971
1.958
1.511
1.295
1.281
1.267
1.024
1.009
0.994
1H NMR, CDCl3
Cl
O
CO2Et
Scheme 2, 3o, X=Br
1.95
1.97
2
3.06
3.08
2.13
2
1.98
2.06
3.07
ppm
9
8
7
6
5
4
3
2
1
0

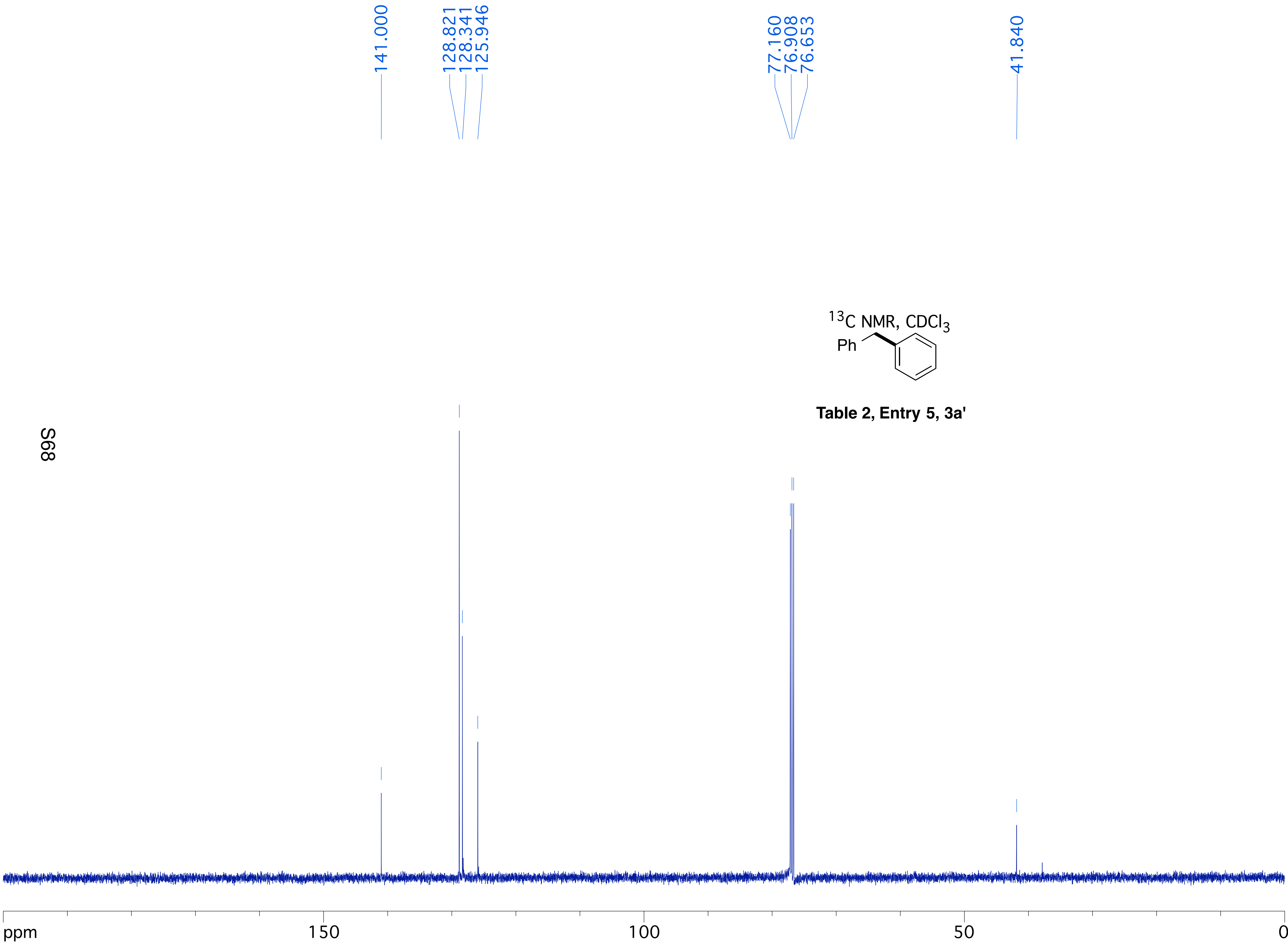
141.000
128.821
128.341
125.946
77.160
76.908
76.653
41.840
13C NMR, CDCl3
Ph
Table 2, Entry 5, 3a'
ppm
150
100
50
0

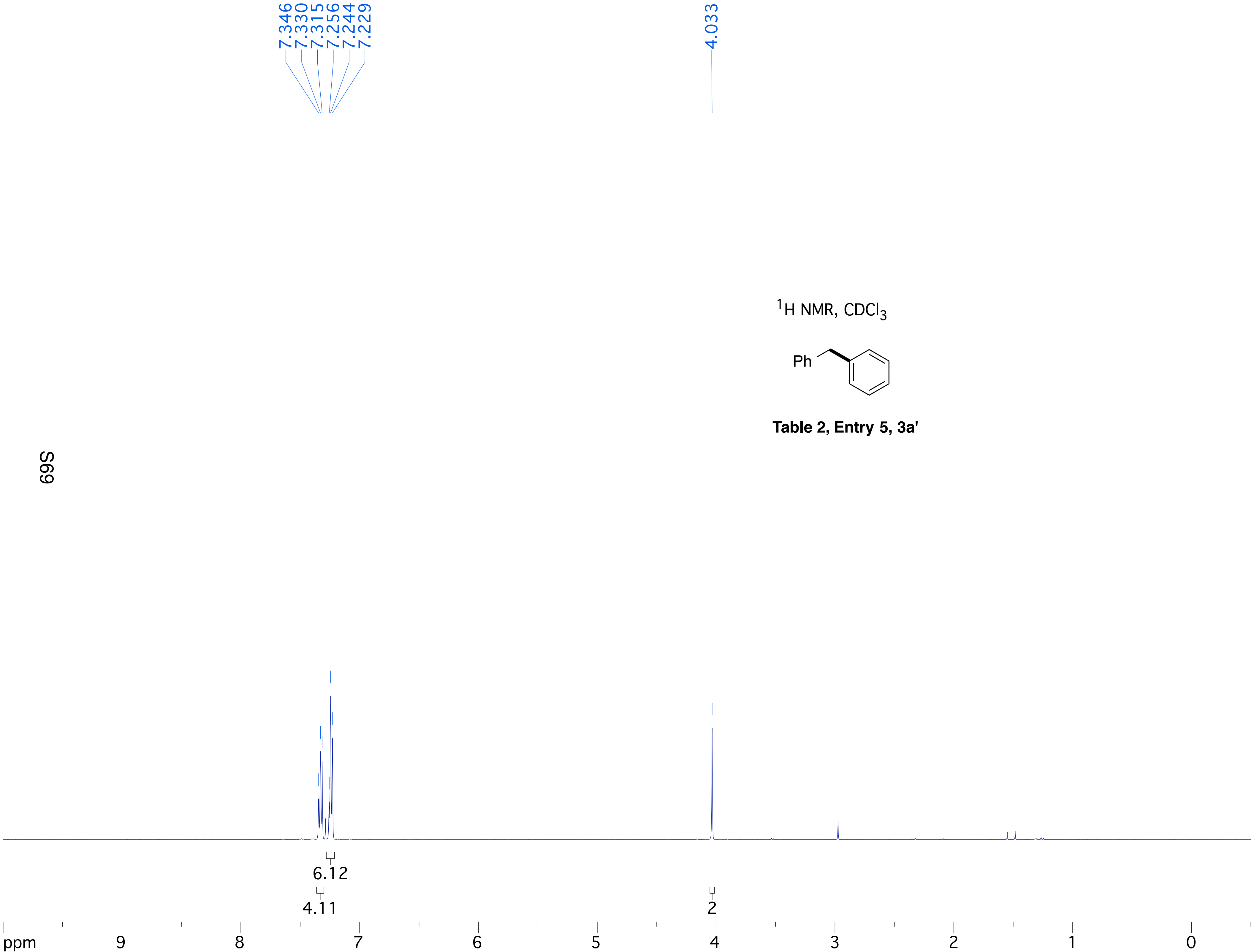

Table 2, Entry 5, 3a'

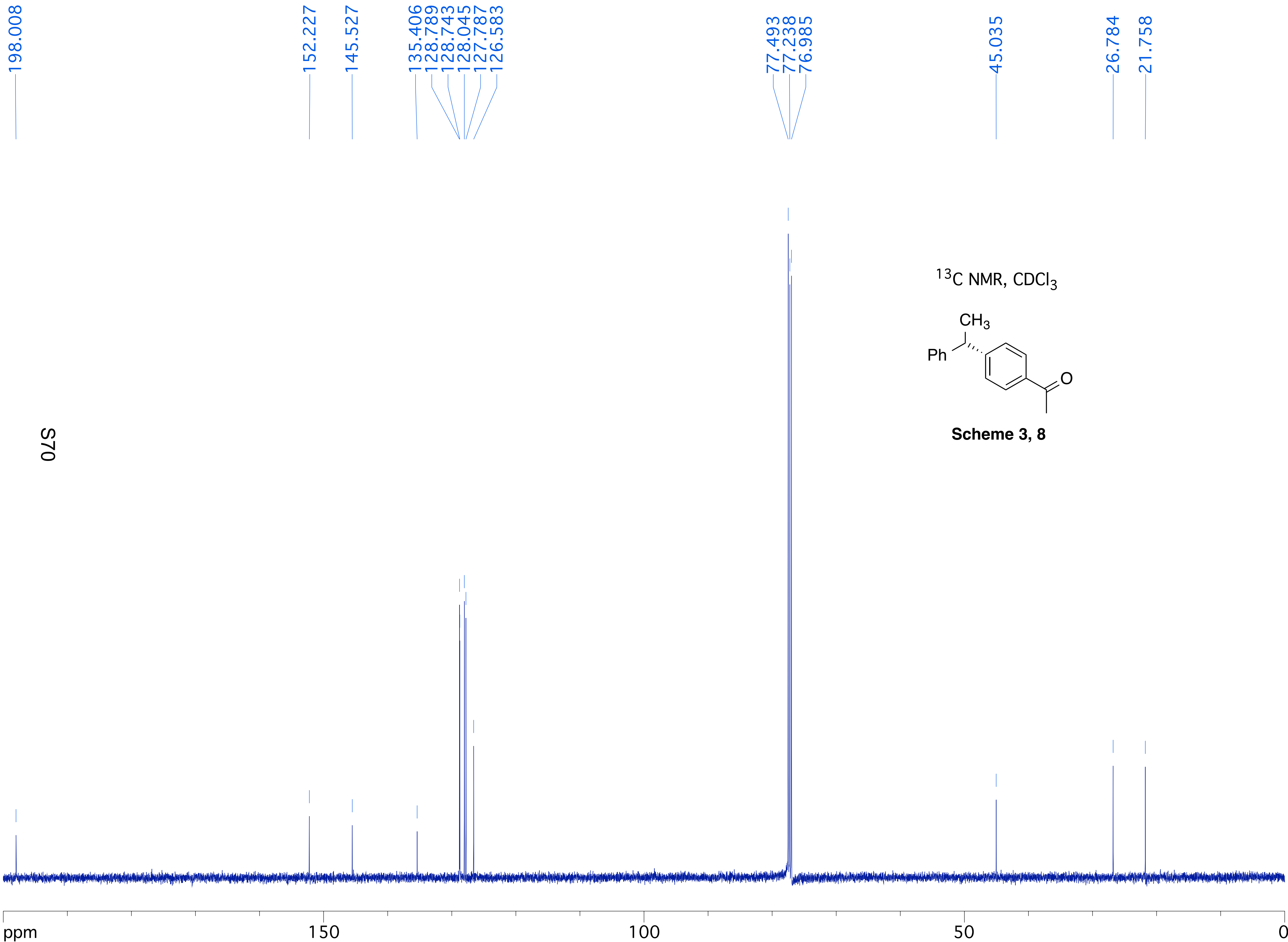
198.008
152.227
145.527
135.406
128.789
128.743
128.045
127.787
126.583
77.493
77.238
76.985
45.035
26.784
21.758
^{13}C NMR, $CDCl_3$
CH3
Ph
O
Scheme 3, 8
ppm
150
100
50
0

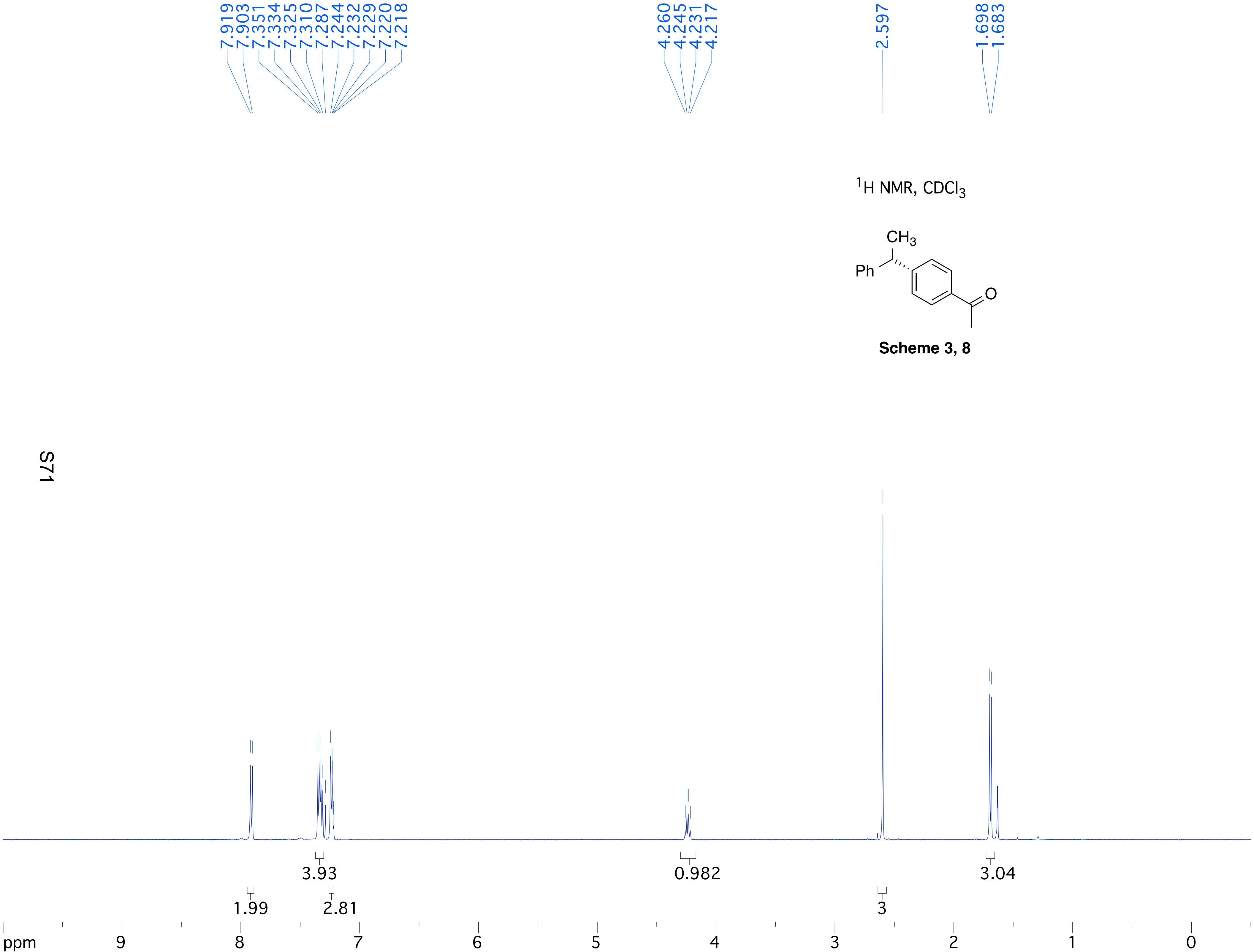

^1H NMR, $CDCl_3$

Scheme 3, 8
